# Decarbonylative ether dissection by iridium pincer complexes†

**DOI:** 10.1039/d0sc03736b

**Published:** 2020-09-24

**Authors:** Changho Yoo, Henry M. Dodge, Alexandra H. Farquhar, Kristen E. Gardner, Alexander J. M. Miller

**Affiliations:** Department of Chemistry, University of North Carolina at Chapel Hill Chapel Hill North Carolina 27599-3290 USA ajmm@email.unc.edu

## Abstract

A unique chain-rupturing transformation that converts an ether functionality into two hydrocarbyl units and carbon monoxide is reported, mediated by iridium(i) complexes supported by aminophenylphosphinite (NCOP) pincer ligands. The decarbonylation, which involves the cleavage of one C–C bond, one C–O bond, and two C–H bonds, along with formation of two new C–H bonds, was serendipitously discovered upon dehydrochlorination of an iridium(iii) complex containing an aza-18-crown-6 ether macrocycle. Intramolecular cleavage of macrocyclic and acyclic ethers was also found in analogous complexes featuring aza-15-crown-5 ether or bis(2-methoxyethyl)amino groups. Intermolecular decarbonylation of cyclic and linear ethers was observed when diethylaminophenylphosphinite iridium(i) dinitrogen or norbornene complexes were employed. Mechanistic studies reveal the nature of key intermediates along a pathway involving initial iridium(i)-mediated double C–H bond activation.

## Abstract 2

A unique chain-rupturing transformation that converts an ether functionality into two hydrocarbyl units and carbon monoxide is reported.

## Graphical abstract

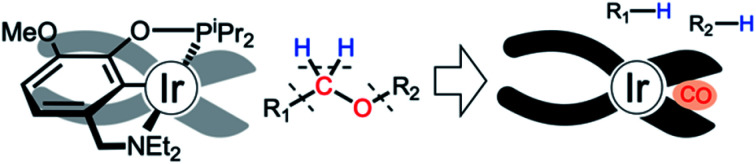

## Introduction

**Scheme 1:**
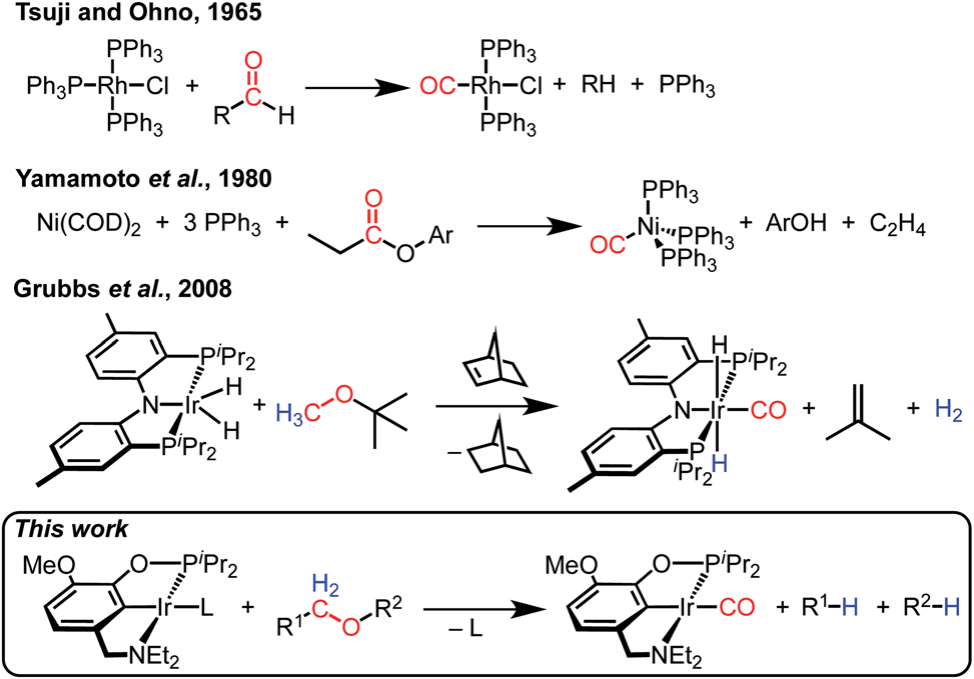
Leading examples of transition metal-mediated decarbonylation.

Decarbonylation reactions, which release carbon monoxide (CO) from organic compounds, are widely utilized in organic synthesis, biomass valorization, and on-demand carbon monoxide reagent generation.^1–7^ The emergence of catalytic decarbonylation methodologies can be traced back to seminal discoveries of stoichiometric transformations. In 1965, Tsuji and Ohno reported that a rhodium complex can effect the stoichiometric decarbonylation of aldehydes to form rhodium carbonyl complexes (Scheme 1).^8^ Subsequent development of catalytic decarbonylation reactions has enabled a range of synthetic methods that utilize aldehydes as precursors to alkanes and alkenes, as cross-coupling reaction partners, and as reagents in transfer hydroformylation.^1,3,4,9,10^ In 1980, Yamamoto and coworkers reported the stoichiometric decarbonylation of esters by nickel complexes, forming nickel carbonyl complexes and alcohols.^11^ This reaction has also subsequently been developed to achieve impressive catalytic transformations, including cross-coupling with ester precursors and biomass upgrading reactions.^1–3,5^ Decarbonylation reactions are dominated by organic carbonyl substrates; CO release from other organic compounds is rare (and often still proceeds *via* carbonyl intermediates, as in alcohol decarbonylation initiated by dehydrogenation to form an aldehyde).^6,7^

Despite the prevalence of ether functionalities in synthetic intermediates and in biomass, only one example of ether decarbonylation has been reported. Romero, Whited, and Grubbs found that a pincer iridium complex reacted with MeO^*t*^Bu to generate a carbonyl complex, isobutene, and H_2_ (Scheme 1).^12^ The stoichiometric reaction is initiated by double C–H bond activation of MeO^*t*^Bu to generate H_2_ and a carbene intermediate, (PNP)Ir=C(H)(O^*t*^Bu), that undergoes isobutene elimination.

Here we present a unique ether decarbonylation reaction that selectively dissects a wide range of ethers, cleaving two C–H bonds, one C–C bond, and one C–O bond, and forming two new C–H bonds to furnish CO and two saturated hydrocarbyl groups. Intramolecular decarbonylation of crown ether groups is described first, followed by extensions to intermolecular reactions. Mechanistic studies provide insight into key intermediates and implicate amine hemilability in controlling the reaction pathway.

## Results and discussion

### Intramolecular ether decarbonylation

**Fig. 1:**
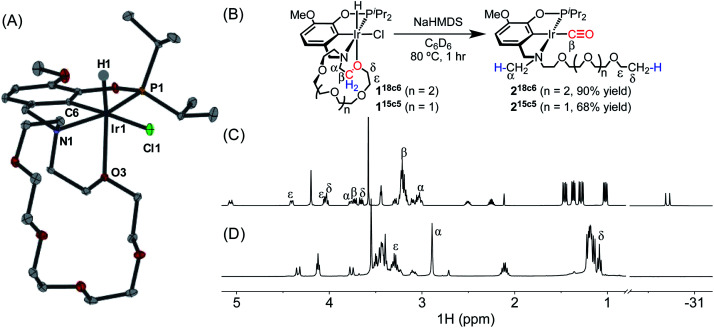
(A) Structural representation of **118c6** with ellipsoids drawn at 50% probability level. (B) Decarbonylation of **118c6** and **115c5**. (C) Partial ^1^H NMR spectrum of **118c6**. (D) Partial ^1^H NMR spectrum of **218c6**.

Preliminary evidence for decarbonylation reactivity was discovered serendipitously while attempting to prepare an iridium(i) complex with a pincer-crown ether ligand. The yellow iridium(iii) precursor (^MeO-18c6^NCOP)Ir(H)(Cl) (**118c6**, Fig. 1) was obtained from the reaction of the known pincer-crown ether ligand (^MeO-18c6^NCOP)H^13^ and [Ir(COD)Cl]_2_. All of the spectroscopic data for **118c6** aligned with expectations for a hydridochloride complex with a single crown ether oxygen donating to complete an octahedral coordination environment. The solid-state structure elucidated from an X-ray diffraction study confirmed the expected tetradentate (κ^4^) pincer ligand binding mode, with one crown ether oxygen bound *trans* to the hydride ligand (Fig. 1A). The bond distances and angles are essentially indistinguishable from the previously characterized 15-crown-5-containing variant.^14^

Base-promoted dehydrochlorination was attempted in pursuit of an iridium(i) complex, inspired by similar procedures developed for other pincer iridium complexes.^12,15–17^ Addition of NaHMDS to a yellow solution of **118c6** in C_6_D_6_ at ambient temperature resulted in an immediate color change to brown and the appearance of multiple species in the ^31^P NMR spectrum. After heating the mixture at 80 °C for 1 h, however, the solution turned yellow and only a single resonance was apparent in the ^31^P NMR spectrum, *δ* 171.32.

The absence of a hydride resonance in the ^1^H NMR spectrum and a *ca.* 30 ppm upfield shift of the phosphinite resonance relative to **118c6** (from *δ* 143.67 to *δ* 171.32) in the ^31^P NMR spectrum provided an early indication that the product was an iridium(i) complex.^13,18^ We initially vetted the spectroscopic data against the possibility of iridium(i) complexes with either (a) a tetradentate pincer-crown ether ligand containing one crown ether oxygen bound, (κ^4-MeO-18c6^NCOP)Ir, or (b) a tridentate pincer-crown ether ligand with N_2_ completing the square planar coordination sphere, (κ^3-MeO-18c6^NCOP)Ir(N_2_). However, neither of these structures could be explained by the NMR data. In particular, a distinctive triplet (*δ* 1.09) and singlet (*δ* 2.90) were present in the ^1^H NMR spectrum, while the crown ether region did not account for all of the expected protons (Fig. 1D). The new triplet and singlet were assigned as unexpected –OCH_2_C*H*_3_ and –NC*H*_3_ groups, respectively, using a combination of multidimensional NMR experiments, including ^1^H–^1^H COSY, ^1^H–^13^C HSQC and ^1^H–^13^C HMBC (Fig. S42–S44, ESI†). The presence of a carbonyl ligand provided an additional surprise, revealed by inspection of ^13^C NMR spectra (*δ* 198.75) and infrared (IR) spectra (*ν*_CO_ = 1931 cm^−1^). On the basis of the combined spectroscopic data, along with electrospray ionization mass spectrometry (ESI-MS) data, the product was assigned as an iridium(i) carbonyl with the ligand crown ether decarbonylated (**218c6**, Fig. 1B). **218c6** was isolated in 90% yield in a preparative-scale experiment.

To probe the generality of this reaction, other iridium complexes containing pendent ethers were examined. The known aza-15-crown-5 complex (^MeO-15c5^NCOP)Ir(H)(Cl) (**115c5**) was prepared as previously described.^19^ Dehydrochlorination of **115c5** with NaHMDS at 80 °C also resulted in intramolecular decarbonylation (Fig. 1), producing an iridium(i) carbonyl species with activated crown ether moiety (**215c5**), which was isolated in 68% yield and was spectroscopically almost identical to the 18-crown-6-derived variant.

**Fig. 2:**
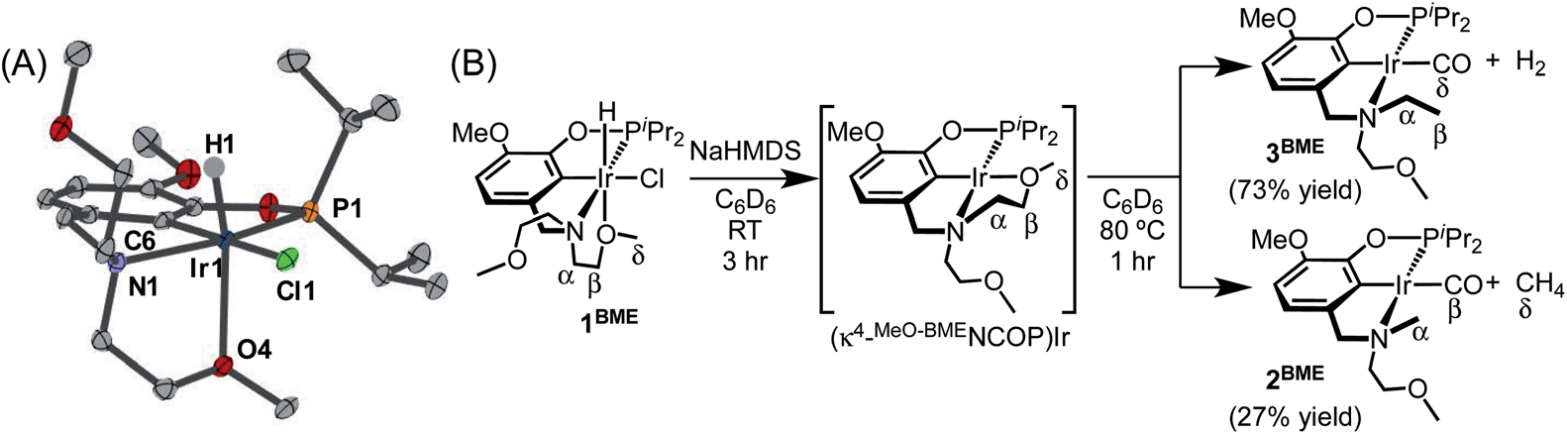
(A) Structural representation of **1BME** with ellipsoids drawn at 50% probability level. (B) Decarbonylation of **1BME**.

An acyclic ligand variant containing a bis(2-methoxyethyl)amine fragment was accessed by reductive amination of iso-vanillin followed by phosphination with ^*i*^Pr_2_PCl. Subsequent metallation with [Ir(COD)Cl]_2_ afforded (^MeO-BME^NCOP)Ir(H)(Cl) (**1BME**). The crystallographically determined solid-state structure of **1BME** shows the expected κ^4^ binding mode, with one methoxy group ligating iridium (Fig. 2A). The distances and angles around the iridium center are very similar to those of **118c6**, but an overlay of **118c6** and **1BME** reveals subtle shifts in the chloride ligand and isopropyl groups consistent with the macrocycle providing a more sterically crowded environment around the metal center (Fig. S69 in the ESI†).

In contrast to the perfectly selective decarbonylative macrocycle dissection in the pincer-crown ether complexes, treatment of **1BME** with base followed by heating at 80 °C produced two products in a 7 : 3 ratio (Fig. 2). The major species (**3BME**) contains an ethylamine, as characterized by a triplet (*δ* 1.30) and quartet (*δ* 3.17) that are correlated in ^1^H–^1^H COSY experiments. This reaction could be balanced by loss of H_2_, which was detected by analyzing headspace using gas chromatography (GC). Although peak overlaps prevented full characterization, the minor species (**2BME**) is tentatively assigned as the decarbonylation product containing a methylamine group. Both H_2_ and CH_4_ were observed in solution as byproducts in these sealed NMR tube experiments.

### Mechanistic studies

The new ether decarbonylation reaction ruptures the crown ether ring by cleavage of two C–H bonds, one C–C bond, and one C–O bond, as well as formation of two C–H bonds. The formerly macrocyclic aza-crown ether is thus transformed into a linear poly(ether) with a methylamine terminus bound to an iridium carbonyl. To our knowledge, the formation of two saturated hydrocarbyl end groups by ether decarbonylation is an unprecedented transformation.

Initial insight into the reaction mechanism can be gleaned from the identity of the product, which reflects the bonds that have been broken and formed. In both macrocyclic complexes **118c6** and **115c6**, the C_α_–C_β_ bond of the macrocycle (Fig. 1) is selectively activated (among multiple possible sites) to produce a methylamine-containing product. The ethylamine-containing product **3BME** (Fig. 2) would derive from decarbonylation of the methoxy end group and release of H_2_ as a byproduct, with the CO carbon derived from the δ position in **1BME**. The overall bond-breaking and bond-forming events involved in the formation of minor product **2BME** are analogous to the crown ether-containing reactions, with C_α_–C_β_ bond cleavage leading to the formation of CH_4_, which was present in ^1^H NMR spectra. The difference in products for macrocyclic and acyclic ethers is striking, with exclusive formation of *N*-methyl products for macrocyclic NCOP complexes and predominant formation of *N*-ethyl products for the acyclic variants. The differing selectivity could be due to the presence of a methoxy group only in the acyclic case, or perhaps due to the different steric profiles (*cf.* X-ray overlay in Fig. S69†).

Further mechanistic insight was gleaned from *in situ* monitoring to probe for reaction intermediates. Treating **118c6** with NaHMDS gave rise to four resonances in the hydride region of ^1^H NMR spectra, which persisted over the course of 10 h at room temperature. After heating at 80 °C for 1 h, all the hydride species disappeared, replaced by the carbonyl complex **218c6**. The complex with the smaller macrocycle, **115c5**, followed an essentially identical course. These results are consistent with initial C–H bond activation.

The reaction of acyclic variant **1BME** with NaHMDS in C_6_D_6_ was monitored over time in a sealed NMR tube at room temperature. After 5 h, **1BME** was converted to a single new species, which did not have any hydride resonances in the ^1^H NMR spectra. The ^1^H NMR spectrum reflects an asymmetric geometry consistent with one methoxy group bound to the iridium center, (κ^4-MeO-BME^NCOP)Ir (Fig. 2). The geminal protons in the methoxyethyl groups are diastereotopic, with the resonances for each CH_2_ pair separated by 0.03–0.61 ppm, consistent with a constrained geometry in close proximity to the metal center (Fig. S54–S58†).^20^ A strong correlation between the phosphorus and one (and only one) methyl of a methoxyethyl group in a ^1^H–^31^P HMBC experiment provides additional evidence for ether ligation (Fig. S57†). NOESY data revealed a slow chemical exchange process between two methoxyethyl groups (*T*_mix_ = 320 ms, 298 K, Fig. S58†), and dipole–dipole coupling between the isopropyl protons and the methoxy protons of one amine substituent. This intermediate was stable at room temperature for 2 days. Heating this intermediate at 80 °C for 1 h gave carbonyl complexes **2BME** and **3BME**.

The different intermediates detected in these experiments are consistent with initial formation of a reactive iridium(i) species that undergoes C–H bond activation prior to decarbonylation. The presence of multiple C–H bond activation products in initial spectra of reactions with pincer–crown ether complexes, which eventually funnel to a single decarbonylation product, suggests rapid equilibria between C–H activation products at 80 °C. We hypothesize that the geometric constraints in the macrocyclic ligand lead to faster C–H bond activation than the acyclic variant.

**Scheme 2:**
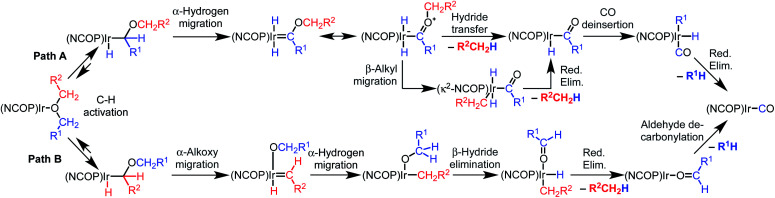
Possible mechanism for decarbonylation.

Based on an initial C–H bond activation, we considered two mechanisms for the decarbonylation reaction, shown in Scheme 2. Both mechanisms start with C–H bond activation to form an alkyl hydride intermediate. In the first mechanism (Path A), the alkyl intermediate undergoes α-hydrogen migration to generate an alkoxycarbene, followed by hydride transfer to the alkoxy group on the carbene to generate a free hydrocarbyl-containing organic product and an acyl iridium complex. Alkyl migration would then produce a carbonyl alkyl hydride complex that undergoes reductive elimination to furnish the final products. In the second mechanism (Path B), the initial alkyl intermediate undergoes α-alkoxy migration followed by β-hydride elimination to generate an aldehyde, which would then undergo decarbonylation.

**Scheme 3:**
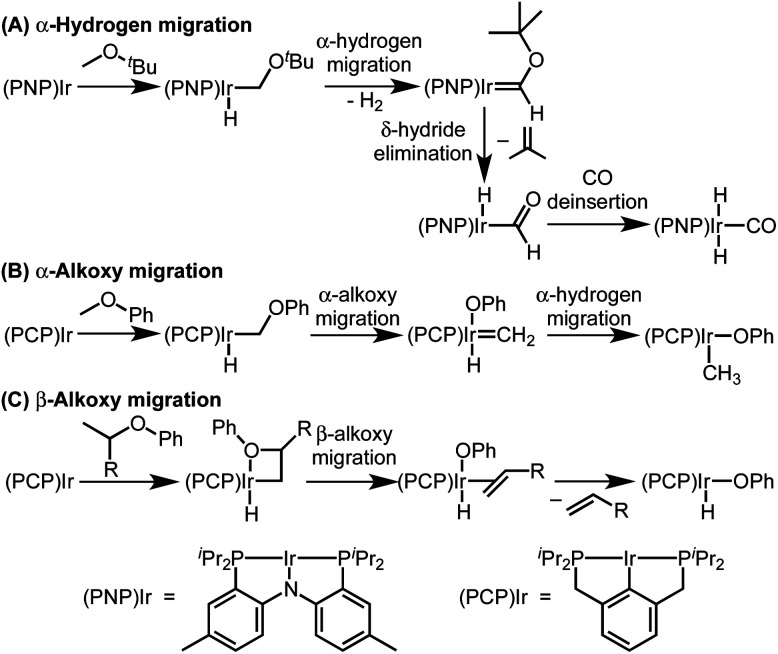
Literature examples of iridium(i)-mediated ether activation *via* C–H activation followed by α-hydrogen migration (A), α-alkoxy migration (B), or β-alkoxy migration (C).

The two possible pathways of Scheme 2 are considered based on the closest literature analogues that could be identified. The most relevant reaction sequence was reported by Grubbs and coworkers, Scheme 3A, where initial C–H bond activation of the methyl group of MeO^*t*^Bu is followed by α-hydrogen elimination to form a carbene.^12,21–28^ The (PNP)Ir system rapidly generated H_2_ after C–H activation and α-hydrogen elimination, leaving a coordinatively unsaturated alkoxycarbene, Ir=C(H)(O^*t*^Bu), that generated isobutene through δ-hydride elimination.^12^

Alkoxy migration also occurs in iridium-mediated net C–O oxidative addition reactions of ethers.^29–36^ As shown in Scheme 3B and C, the reactions developed by the Goldman group undergo initial C–H bond activation, but alkoxide elimination occurs (either α- or β-elimination depending on the site of C–H activation) preferentially to α-hydrogen elimination for the (PCP)Ir system.^28,34–40^ Although decarbonylation was not observed in the examples of Scheme 3B and C, these phenyl ether substrates could not undergo the β-hydride elimination as an alkyl ether would in Path B in Scheme 2.

The particular elimination process that follows formation of the alkyl intermediate seems to control the selectivity between decarbonylation, dehydrogenation, or C–O oxidative addition pathways. Additional studies to distinguish between the pathways of Scheme 2 are presented in intermolecular reactions below.

### Isolation of iridium(i) dinitrogen and norbornene complexes

With the goal of exploring intermolecular decarbonylation reactions, an analogous diethylamino–NCOP ligand was utilized in order to isolate iridium(i) species without ligand activation. Metallation of the known diethylamine-containing ligand, (^Et^NCOP)H,^41^ with [Ir(COD)Cl]_2_ was initially attempted, but this gave multiple species, presumably due to the undesired metallation *ortho* to the phosphinite. The new methoxy-substituted ligand (^MeO-Et^NCOP)H was prepared by a procedure similar that employed in the synthesis of (^MeO-BME^NCOP)H and metallated to form [(^MeO-Et^NCOP)Ir(H)(Cl)]_2_ (**1Et**). The hydride resonance (*δ* −39.01 in C_6_D_6_) in the ^1^H NMR spectrum is slightly downfield of other square pyramidal iridium hydridochloride complexes possessing a vacant coordination site *trans* to the hydride.^12,42–47^ Complex **1Et** is poorly soluble in CH_2_Cl_2_ and benzene, but highly soluble nature in coordinating solvents (THF and MeCN), which suggested that **1Et** might adopt a dimeric structure in the absence of coordinating solvents. An X-ray diffraction study of crystals of **1Et**, grown from CH_2_Cl_2_/pentane, revealed a diiridium complex with bridging chloride ligands each sitting *trans* to hydride.

**Fig. 3:**
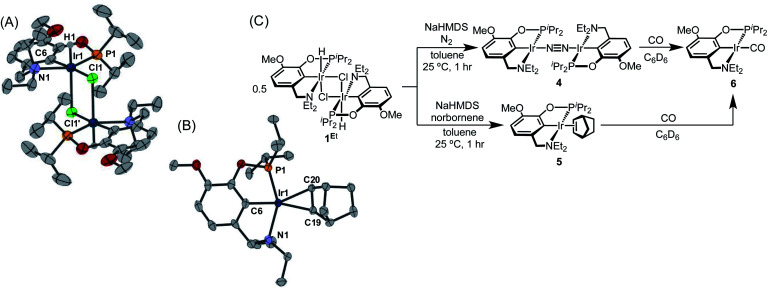
(A) Structural representation of **1Et** and (B) **5** with ellipsoids drawn at 50% probability level. (C) Synthesis of iridium(i) complexes.

Dehydrochlorination of **1Et** by NaHMDS under N_2_ cleanly generated a new iridium complex. The hydride resonance completely disappeared in ^1^H NMR spectra and the phosphinite resonance was shifted *ca.* 20 ppm upfield relative to **1Et** (from *δ* 147.44 to *δ* 163.68), indicating formation of the iridium(i) species. The same reaction under Ar resulted in formation of multiple unidentified hydride species, raising the possibility of N_2_ coordination. The ion peaks observed by ESI-MS revealed the N_2_-bridged dimer [(^MeO-Et^NCOP)Ir]_2_(μ-N_2_) (**4**, Fig. 3). The ^1^H NMR spectrum of **4** is consistent with *C*_2_-type symmetry and the presence of a bridging N_2_ ligand in the solid state was confirmed by resonance Raman spectroscopy (*ν*_NN_ = 2017 cm^−1^). No solution IR stretch corresponding to a terminal N_2_ complex was observed. The bridging N_2_ ligand in **4** is less activated compared to similar pincer iridium(i) N_2_ complex [(PCP)Ir]_2_(μ-N_2_) (1979 cm^−1^).^48^ Complex **4** was isolated in 56% yield and can be stored as a solid under N_2_, but underwent 60% decomposition after 15 days at room temperature in C_6_D_6_.

Seeking a more thermally stable iridium(i) synthon, dehydrochlorination of **1Et** was performed in the presence of norbornene. The norbornene adduct (^MeO-Et^NCOP)Ir(NBE) (**5**, Fig. 3) was isolated in 90% yield. The bound norbornene ligand was clearly indicated by NMR spectroscopic data and X-ray diffraction study (Fig. 3). Complex **5** is somewhat more stable than **4**, undergoing just 15% decomposition after 15 days at room temperature in C_6_D_6_. Both N_2_ and norbornene ligands can be replaced upon addition of CO to give (^MeO-Et^NCOP)Ir(CO) (**6**) quantitatively.

### Intermolecular ether decarbonylation

The reactivity of dinitrogen complex **4** with various ethers was explored by heating benzene solutions at 80 °C for 24 h in Teflon-sealed NMR tubes. Table 1 summarizes the results. In each case, carbonyl complex **6** was observed, with the yield varying as a function of ether identity and concentration. The yield of carbonyl complex **6** was measured by quantitative ^31^P NMR spectroscopy using triphenyl phosphate as an internal standard. The organic products were analyzed by NMR spectroscopy and headspace GC.

**Table 1:** Intermolecular ether decarbonylation by **4**

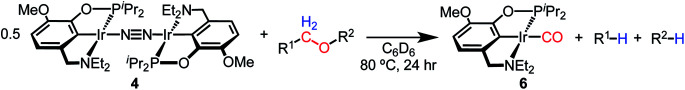				
Ether	Ether/Ir ratio^a^	**6** yield^c^^,^^d^	Organic product	Organic yield^e^
18-Crown-6	1	5%	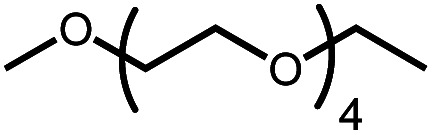	n.q.^f^
	10	42%		37%^g^
12-Crown-4	1	3%	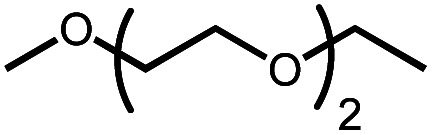	n.q.
	10	26%		23%^g^
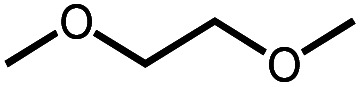	10	2%	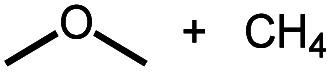	n.q.
	530^b^	10%		n.q.
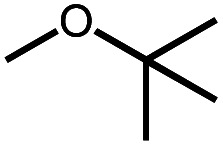	10	7%	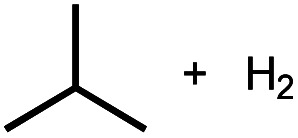	8%^h^
	100	18%		15%^h^
	450^b^	67%		67%^h^
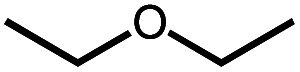	10	5%	Not detected	n.q.
	530^b^	15%		n.q.
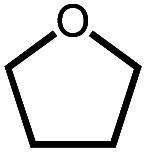	680^b^	2%	Not detected	n.q.
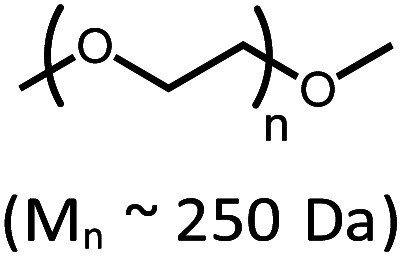	10	21%	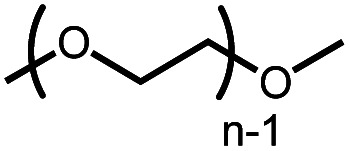	n.q.

a Equivalents of ether relative to one iridium center.

b The substrate was used as a solvent.

c Yield of **6** obtained from quantitative ^31^P NMR relative to **4** used.

d >99% of **4** was consumed for all cases.

e Yield of organic product relative to **4** used.

f Not quantified due to low yield of **6**.

g Yield obtained from quantitative ^13^C NMR.

h Yield of (HC(CH_3_)_3_) obtained from quantitative ^1^H NMR integration.

The activation of macrocyclic ethers resulted in formation of linear poly(ether) products and the corresponding Ir carbonyl complex **6**. Di- and tetra(ethylene glycol) ethyl methyl ether were obtained from the decarbonylation of 12-crown-4 and 18-crown-6, respectively. The yield of poly(ether) product was similar to that of (^MeO-Et^NCOP)Ir(CO), as expected.

Intermolecular reactions of linear ethers produced two saturated hydrocarbyl coproducts. Treating glyme with **4** produced carbonyl complex **6** along with methane and dimethyl ether. Grubbs and coworkers only reported ether decarbonylation of MeO^*t*^Bu, so this substrate is an important point of comparison. The prior study converted MeO^*t*^Bu to isobutene and a dihydridocarbonyl complex.^12^ In contrast, complex **4** reacts with MeO^*t*^Bu to produce isobutane, H_2_, and carbonyl complex **6**. Although low yields precluded comprehensive studies of other substrates, the MeO^*t*^Bu experiment effectively rules out α-alkoxide migration and suggests that Path B of Scheme 2 is not operative. An α-alkoxide migration would produce a *tert*-butoxide intermediate incapable of β-hydrogen elimination. The fact that the reaction still produces an iridium carbonyl product suggests that a pathway featuring α-hydrogen migration is likely operative, as shown in Path A in Scheme 2. The extreme steric congestion of a putative Ir^V^–^*t*^Bu complex that would be formed by alkyl migration suggests that the hydride transfer route of Path A may be more likely.

Even simple ethers like Et_2_O and THF can be decarbonylated by **4**, albeit in low yield (Table 1). Although double C–H bond activation of THF is commonly observed at iridium complexes,^25,26,37–40,49^ no stable alkoxycarbene species was observed in the THF reactions.

Considering current interest in polymer degradation and recycling, we also tested the poly(ether) substrate poly(ethylene glycol), PEG (Table 1). The reaction of **4** with methyl-terminated PEG (*M*_n_ ∼ 250 Da) resulted in 21% yield of iridium carbonyl **6**. The PEG sample employed was dominated by pentamer, hexamer and heptamer peaks observed by atmospheric pressure chemical ionization mass spectroscopy. No trimer or tetramer peaks were apparent initially. After treatment with complex **4**, however, new peaks for trimer and tetramer appeared in the mass spectrum, indicating successful decarbonylation (Fig. S62 in the ESI†).

The yield of the carbonyl complex generally increased with increasing concentration of substrate (Table 1). We hypothesized that the higher concentrations of substrate promoted productive reactivity, outcompeting background degradation of the complex (complete decomposition observed after heating at 80 °C for 6 h in the absence of substrate). Considering that the poor stability of **4** under the reaction conditions was likely a primary contributor to the low yields for intermolecular reactions, we explored different iridium precursors.

In an attempt to improve the yield of decarbonylation, we tested intermolecular reactions between ethers and the norbornene complex (^MeO-Et^NCOP)Ir(NBE) (**5**). We hypothesized that the improved thermal stability of norbornene adduct **5** relative to **4** (60% decomposition *vs.* complete decomposition in 6 h at 80 °C) would lead to higher yields of the desired products. The results are summarized in Table 2. While complex **5** decarbonylates the macrocycles 18-crown-6 and 12-crown-4 in a similar fashion to **4**, higher yields of carbonyl complex **6** were observed during glyme and diethyl ether activation.

**Table 2:** Intermolecular ether decarbonylation using Ir(i) pincer complexes

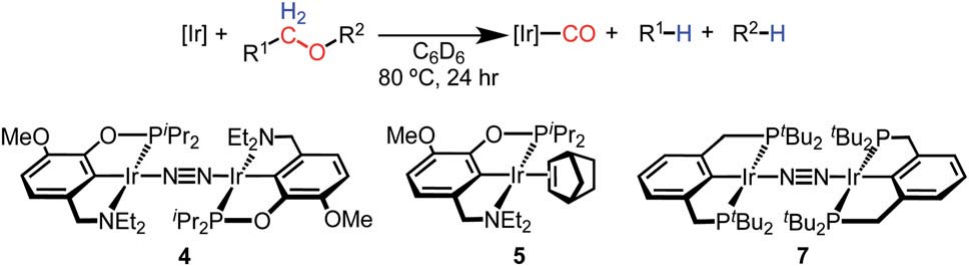				
Ir	Substrate	Conversion of SM^d^	Yield of carbonyl^c^	Yield of organic^e^
**4**	18-Crown-6 (10 eq.^a^)	>99%	42%	37%
	12-Crown-4 (10 eq.^a^)	>99%	26%	23%
	Glyme (530 eq.^a^^,^^b^)	>99%	10%	—
	Et_2_O (530 eq.^a^^,^^b^)	>99%	15%	—
**5**	18-Crown-6 (10 eq.^a^)	>99%	42%	49%
	12-Crown-4 (10 eq.^a^)	>99%	27%	23%
	Glyme (530 eq.^a^^,^^b^)	>99%	39%	—
	Et_2_O (530 eq.^a^^,^^b^)	>99%	34%	—
**7**	18-Crown-6 (10 eq.^a^)	35%	12%	Trace
	12-Crown-4 (10 eq.^a^)	67%	3%	Trace
	Glyme (530 eq.^a^^,^^b^)	76%	11%	—
	Et_2_O (530 eq.^a^^,^^b^)	<1%	<1%	—

a Amount of ether relative to one iridium center.

b The substrate was used as a solvent.

c Yield of (L)Ir(CO) obtained from quantitative ^31^P NMR relative to **4** used.

d Consumption of the iridium compound from quantitative ^31^P NMR relative to the initial amount of the iridium compound used.

e Yield obtained from quantitative ^13^C NMR.

A complex with a pincer ligand of renowned stability, [(PCP)Ir]_2_(μ-N_2_) (**7**),^48^ was also examined. (PCP)Ir platforms exhibit exceptional stability at high temperatures, as established in studies of alkane dehydrogenation,^50^ and we observed no significant decomposition after 5 days at 80 °C in the presence of Et_2_O. Furthermore, (PCP)Ir complexes facilitate net C–O oxidative addition of a range of substrates^34–36^—although there are no reports of ether decarbonylation with this system. Surprisingly, reactions of complex **7** with 18-crown-6, 12-crown-4, and glyme produced (PCP)Ir(CO) in poor yield (Table 2). There was no detectable conversion with Et_2_O. Even in cases where (PCP)Ir(CO) was formed, only trace amounts of decarbonylated organic product (<1% yield) were detected. The diphosphine-based pincer complex is thus essentially unable to carry out the chemistry observed for the aminophosphinite-based complexes, despite excellent stability.

The distinct reactivity of (NCOP)Ir and (PCP)Ir complexes suggests a possible role of amine hemilability in determining the selectivity of ether decarbonylation. In the intramolecular reactions, amine dissociation seems inevitable in order to accommodate appropriate conformations for C–H bond activation and subsequent elimination reactions. In intermolecular reactions, amine dissociation could facilitate alkyl migration (lower route of Path A in Scheme 2) or provide flexibility needed for the bulky alkoxy(alkyl)carbene complex to achieve the appropriate conformation for hydride transfer (upper route of Path A in Scheme 2). However, influences of steric (isopropyl/ethyl *vs. tert*-butyl) or electronic differences of the pincer ligands cannot be completely ruled out.^51^

The studies comparing different iridium precursors provide some insight into the low yields of the intermolecular reactions. With complexes **4** and **5**, the yield is limited by background decomposition of the iridium complexes. With complex **7**, stability is achieved but the desired reaction is not observed. These results guide research towards new ligand motifs that include hemilabile amine donors with improved stability.

**Scheme 4:**
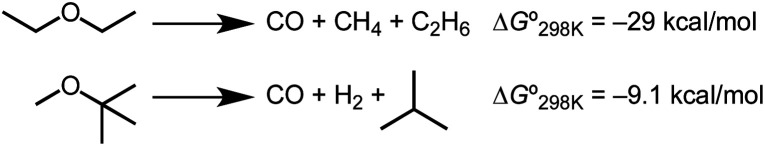
Thermodynamics of ether decarbonylation.^53–56^

The formation of stable carbonyl adducts represents another limitation that must be overcome to achieve catalysis. Importantly, the stabilization conferred by CO binding to iridium is not required to drive the decarbonylation reactions. The ether decarbonylation reactions of Scheme 4 are estimated to be exergonic under standard conditions (Tables S1 and S2, ESI†). Methods to promote CO discoordination^52^ or tandem reactions that consume CO^6,7,10^ could be explored in future work aimed at achieving catalytic turnover. Considering the broad utility of decarbonylation methods based on aldehydes, esters, and alcohols, new catalytic ether decarbonylation reactions could find utility in distinct biomass conversion schemes, in cross-coupling of ethers, and other applications.^1–7^

## Conclusions

The decarbonylative cleavage of ethers into CO and hydrocarbyl fragments is mediated by iridium(i) pincer complexes. The reaction features an extraordinary number of bond-breaking (two C–H bonds, one C–C bond, and one C–O bond) and bond-forming (two C–H bonds) events.

Intramolecular ether activation was discovered serendipitously while studying pincer-crown ether complexes. These reactions proceed in excellent yield and high selectivity, with a change in the site of decarbonylation observed for acyclic pendent ethers relative to the macrocyclic variants. Using a diethylamine-based pincer ligand enabled an intermolecular variant of the reaction, where the pincer ligand remains intact while reacting with free ethers.

Mechanistic studies revealed initial C–H bond activation by an iridium(i) species. The efficient decarbonylation of MeO^*t*^Bu rules out the α-alkoxy migration pathway and suggests the α-hydrogen migration is the operative pathway. The conversion with a PCP iridium(i) complex was much lower than that of NCOP complexes, confirming the importance of ligand design in conferring the desired reactivity and suggesting a possible role of amine hemilability.

Collectively, these results raise hopes of future development of a suitable ligand that can promote the desired reaction while maintaining suitable thermal stability to release the CO ligand and achieve turnover.

## Conflicts of interest

There are no conflicts to declare.

## Supplementary Material

SC-011-D0SC03736B-s001

SC-011-D0SC03736B-s002
