# Supplementary material for: Decarbonylative ether dissection by iridium pincer complexes
Source: Chem Sci. 2020 Sep 24;11(44):12130–8. doi: 10.1039/d0sc03736b (PMC8162749; doi:10.1039/d0sc03736b)
Supplement: SC-011-D0SC03736B-s001 [file SC-011-D0SC03736B-s001.pdf]

Electronic Supplementary Material (ESI) for Chemical Science.
This journal is © The Royal Society of Chemistry 2020

Electronic Supplementary Information

Decarbonylative Ether Dissection by Iridium Pincer Complexes

Changho Yoo, Henry M. Dodge, Alexandra H. Farquhar, Kristen E. Gardner, and Alexander J. M. Miller*

[†] *Department of Chemistry, University of North Carolina at Chapel Hill, Chapel Hill, North Carolina 27599–3290, United States*

*Corresponding Author Email Address: ajmm@email.unc.edu

Table of Contents

I. Experimental Section **S2**
II. NMR Spectra of New Compounds **S13**
III. 2-Dimensional NMR Spectra **S26**
IV. Yield Determination for Intermolecular Decarbonylation **S38**
V. Infrared and Resonance Raman Spectra **S41**
VI. Thermodynamics of Ether Decarbonylation **S43**
VII. Crystallographic Details **S44**
VIII. References **S59**

I. Experimental Section

General Considerations.

All manipulation was carried out using standard Schlenk or glovebox techniques under a N_2 atmosphere, except as noted. Organic solvents were dried and degassed with argon using a Pure Process Technology solvent system and stored over 3 Å molecular sieves. Under standard glovebox operating conditions, pentane, diethyl ether, benzene, toluene, and tetrahydrofuran were used without purging, such that traces of those solvents were present in the atmosphere and in the solvent bottles. ^{1}H, ^{31}P{^{1}H}, and ^{13}C{^{1}H} spectra were recorded on 400, 500, or 600 MHz spectrometers at 298 K. NMR solvents were purchased from Cambridge Isotopes Laboratories, Inc. Benzene-d_6 (C_6D_6) was freeze−pump−thaw degassed three times before drying by passage through a small column of activated alumina and storage over 3 Å molecular sieves. ^{1}H and ^{13}C NMR chemical shifts are reported in ppm relative to residual solvent resonances. ^{31}P NMR chemical shifts are reported versus phosphoric acid (0 ppm) using absolute referencing to the ^{1}H resonance of the residual solvent.[1] The compounds ($^{MeO\text{-}18c6}$NCOP)H,[2] ($^{MeO\text{-}15c5}$NCOP)H,[3] $[Ir(COD)Cl]_2$[4] and ($^{MeO\text{-}15c5}$NCOP)Ir(H)(Cl) (**1^{15c5}**)[5] were synthesized according to the literature procedures. Infrared (IR) spectroscopy was carried out with a Thermo Scientific Nicolet iS5 FT-IR. High-resolution mass spectrometry (HRMS) was carried out at the University of North Carolina at Chapel Hill Department of Chemistry Mass Spectrometry Core Laboratory on a Thermo Scientific Q Exactive HF-X mass spectrometer. Uncorrected Raman spectra were collected by using a Renishaw inVia Raman microscope equipped with a CCD detector and 1800 lines/mm grating. Laser excitation at 633 nm was passed to a Leica 2700M microscope outfitted with a 50X N-Plan Leica objective. CCD exposure time was 10–30 s. Elemental analyses were performed by Robertson Microlit Laboratories (Ledgewood, NJ).

Synthesis of ($^{MeO\text{-}18c6}$NCOP)Ir(H)(Cl) (1^{18c6}).

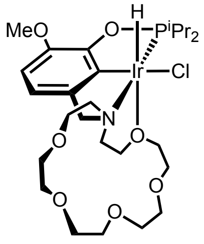

An orange mixture of ($^{MeO\text{-}18c6}$NCOP)H (580 mg, 1.12 mmol) and $[Ir(COD)Cl]_2$ (378 mg, 0.562 mmol) in 10 mL toluene was stirred at 80 °C overnight. The resulting yellow solution was cooled to room temperature and the volatiles were removed under vacuum. The residue was washed with ether and dried under vacuum to give a light yellow powder of ($^{MeO\text{-}18c6}$NCOP)Ir(H)(Cl) (**1^{18c6}**) (756 mg, 91% yield). Crystals suitable for X-ray diffraction were grown by layering a concentrated toluene solution of **1^{18c6}** with pentane. **^{1}H NMR** (600 MHz, C_6D_6) δ 6.58 (d, *J* = 8.0 Hz, 1H, Ar-*H*), 6.46 (d, *J* = 8.0 Hz, 1H, Ar-*H*), 5.06 (dt, *J* = 15.6, 5.1 Hz, 1H, crown-C*H*$_2$), 4.40 (ddd, *J* = 12.1, 5.4, 2.5 Hz, 1H, crown-C*H*$_2$), 4.20 (d, *J* = 1.9 Hz, 2H, ArC*H*$_2$N), 4.08 – 3.97 (m, 2H, crown-C*H*$_2$), 3.79 – 3.69 (m, 2H, crown-C*H*$_2$), 3.66 (dd, *J* = 13.6, 6.4 Hz, 1H, crown-C*H*$_2$), 3.58 (s, 3H, Ar-OC*H*$_3$), 3.44 (t, *J* = 4.6 Hz, 2H, crown-C*H*$_2$), 3.29 (ddd, *J* = 10.9, 7.1, 1.8 Hz, 1H, crown-C*H*$_2$), 3.26 – 3.14 (m, 10H, crown-C*H*$_2$), 3.10 (ddd, *J* = 11.1, 5.3, 1.7 Hz, 1H, crown-C*H*$_2$), 3.08 – 2.97 (m, 3H, crown-C*H*$_2$), 2.56 – 2.45 (m, 1H, C*H*(CH$_3$)$_2$), 2.30 – 2.20 (m, 1H, C*H*(CH$_3$)$_2$), 1.46 (dd, *J* = 16.6, 7.5 Hz, 3H, CH(C*H*$_3$)$_2$), 1.37 (dd, *J* = 13.5, 7.0 Hz, 3H, CH(C*H*$_3$)$_2$), 1.28 (dd, *J* = 18.8, 6.9 Hz, 3H, CH(C*H*$_3$)$_2$), 1.02 (dd, *J* = 15.3, 6.9 Hz, 3H, CH(C*H*$_3$)$_2$), –30.71 (d, *J* = 26.2 Hz, 1H, Ir-*H*). **^{13}C{^{1}H} NMR** (151 MHz, C_6D_6)) δ 152.06 (d, *J* = 4.4 Hz, *C*$_{Ar}$), 143.59 (d, *J* = 12.1 Hz, *C*$_{Ar}$), 140.60 (d, *J* = 2.9 Hz, *C*$_{Ar}$), 138.62 (d, *J* = 4.9 Hz, *C*$_{Ar}$), 114.48 (*C*$_{Ar}$), 107.93 (*C*$_{Ar}$), 72.78 (Ar*C*H$_2$N), 72.36 (crown-*C*H$_2$), 72.22 (crown-*C*H$_2$), 71.89 (crown-*C*H$_2$), 71.14 (crown-*C*H$_2$), 70.89 (crown-*C*H$_2$), 70.74 (crown-*C*H$_2$), 70.59 (crown-*C*H$_2$), 70.00 (crown-*C*H$_2$), 69.41 (crown-*C*H$_2$), 68.14 (crown-*C*H$_2$), 61.63 (crown-*C*H$_2$), 59.82 (crown-*C*H$_2$), 55.92 (Ar-O*C*H$_3$), 31.70 (d, *J* = 32.4 Hz, CH(CH$_3$)$_2$), 30.21 (d, *J* = 38.1 Hz, *C*H(CH$_3$)$_2$), 17.93 (d, *J* = 7.3 Hz, CH(*C*H$_3$)$_2$), 17.76 (CH(*C*H$_3$)$_2$), 17.24 (d, *J* = 4.0 Hz, CH(*C*H$_3$)$_2$), 16.71 (d, *J* = 3.0 Hz, CH(*C*H$_3$)$_2$). **^{31}P{^{1}H} NMR** (162 MHz, C_6D_6) δ 143.67. **HRMS** (ESI$^+$) m/z: $[M - Cl^-]^+$ calcd 708.2641; found 708.2639. **Anal. Calcd** for $C_{26}H_{46}ClIrNO_7P$: C, 42.01; H, 6.24; N, 1.88. Found: C, 42.28; H, 6.24; N, 1.80.

Decarbonylation of 1^{18c6} to form 2^{18c6}.

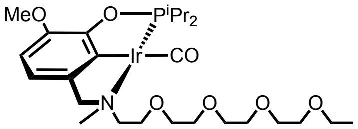

To a stirring suspension of ($^{MeO-18c6}$NCOP)Ir(H)(Cl) ($\mathbf{1^{18c6}}$, 50 mg, 0.067 mmol) in 5 mL toluene was added NaHMDS (13 mg, 0.071 mmol), resulting in an immediate color change from yellow to orange. The reaction mixture was stirred at 80°C overnight. The resulting yellow solution was filtered through short alumina plug and the filtrate was dried under vacuum. The residue was washed with pentane to give an orange oil of $\mathbf{2^{18c6}}$ (43 mg, 90% yield). **^{1}H NMR** (600 MHz, C_6D_6) δ 6.74 (d, *J* = 7.9 Hz, 1H, Ar-*H*), 6.53 (d, *J* = 7.8 Hz, 1H, Ar-*H*), 4.34 (d, *J* = 14.1 Hz, 1H, Ar-C*H*HN), 4.12 (t, *J* = 5.5 Hz, 2H, NCH_2CH_2O-), 3.77 (d, *J* = 14.2 Hz, 1H, Ar-CH*H*N), 3.55 (s, 3H, Ar-OCH_3), 3.53 – 3.22 (m, 13H, -OC_2H_4O-, -OCH_2CH_3 and NC*H*HCH$_2$O-), 3.13 – 3.06 (m, 1H, NCH*H*CH$_2$O-), 2.90 (s, 3H, NCH_3), 2.11 (h, *J* = 7.0 Hz, 2H, $CH(CH_3)_2$), 1.18 (td, *J* = 18.4, 17.1, 6.9 Hz, 12H, $CH(CH_3)_2$), 1.09 (t, *J* = 7.0 Hz, 3H, -OCH_2CH_3). **^{13}C{^{1}H} NMR** (151 MHz, C_6D_6) δ 198.75 (Ir-*C*O), 169.34 (d, *J* = 7.5 Hz, C_{Ar}), 154.23 (d, *J* = 7.1 Hz, C_{Ar}), 145.93 (d, *J* = 2.5 Hz, C_{Ar}), 143.68 (d, *J* = 13.6 Hz, C_{Ar}), 115.53 (C_{Ar}), 112.43 (C_{Ar}), 74.59 (ArCH_2N), 71.18 (-OC_2H_4O-), 71.14 (-OC_2H_4O-), 71.07 (-OC_2H_4O-), 71.01 (-OC_2H_4O-), 70.80 (-OC_2H_4O-), 70.68 (-OC_2H_4O-), 70.44 (-OC_2H_4O-), 66.62 (NCH_2CH_2O-), 65.96 (NCH_2CH_2O-), 56.24 (Ar-OCH_3), 53.53 (d, *J* = 2.8 Hz, NCH_3), 31.70 ($CH(CH_3)_2$), 31.45 ($CH(CH_3)_2$), 18.57 (d, *J* = 5.1 Hz, $CH(CH_3)_2$), 18.46 (d, *J* = 4.9 Hz, $CH(CH_3)_2$), 17.83 ($CH(CH_3)_2$), 17.71 ($CH(CH_3)_2$), 15.55 (-OCH_2CH_3). **^{31}P{^{1}H} NMR** (162 MHz, C_6D_6) δ 171.32. **IR** (THF, cm^{-1}): *ν*(CO) 1931. **HRMS** (ESI^+) m/z: $[M + H]^+$ calcd for $C_{26}H_{46}IrNO_7P$ 708.2641; found 708.2612. **Anal. Calcd** for $C_{26}H_{45}IrNO_7P$: C, 44.18; H, 6.42; N, 1.98. Found: C, 43.68; H, 6.15; N, 2.13.

Decarbonylation of 1^{15c5} to form 2^{15c5}.

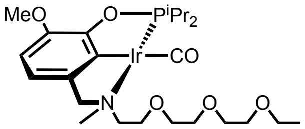

To a stirring suspension of ($^{MeO-15c5}$NCOP)Ir(H)(Cl) ($\mathbf{1^{15c5}}$, 50 mg, 0.072 mmol) in 5 mL toluene was added NaHMDS (13 mg, 0.071 mmol) resulting in an immediate color change from yellow to orange. The reaction mixture was stirred at 80°C overnight. The resulting yellow solution was filtered through short alumina plug and the filtrate was dried under vacuum. The residue was

washed with pentane to give an orange oil of **2**[15c5] (32 mg, 68% yield). **^{1}H NMR** (600 MHz, C_6D_6) δ 6.74 (d, *J* = 7.9 Hz, 1H, Ar-*H*), 6.52 (d, *J* = 7.9 Hz, 1H, Ar-*H*), 4.33 (d, *J* = 14.2 Hz, 1H, ArC*H*HN), 4.13 (t, *J* = 5.4 Hz, 2H, NCH_2CH_2O-), 3.75 (d, *J* = 14.1 Hz, 1H, ArCH*H*N), 3.54 (s, 3H, Ar-OCH_3), 3.50 – 3.38 (m, 4H, -OC_2H_4O- and -OCH_2CH_3), 3.35 – 3.19 (m, 3H, -OC_2H_4O- and NC*H*HCH_2O-), 3.12 – 3.05 (m, 1H, NCH*H*CH_2O-), 2.89 (s, 3H, NCH_3), 2.11 (h, *J* = 6.9 Hz, 2H, C*H*$(CH_3)_2$), 1.23 – 1.14 (m, 12H, $CH(CH_3)_2$), 1.09 (t, *J* = 6.9 Hz, 3H, -OCH_2CH_3). **^{13}C{^{1}H} NMR** (151 MHz, C_6D_6) δ 198.76 (Ir-*C*O), 169.33 (d, *J* = 7.7 Hz, C_{Ar}), 154.21 (d, *J* = 7.2 Hz, C_{Ar}), 145.89 (d, *J* = 2.8 Hz, C_{Ar}), 143.67 (d, *J* = 13.7 Hz, C_{Ar}), 115.52 (C_{Ar}), 112.31 (C_{Ar}), 74.57 (ArCH_2N), 71.20 (-OC_2H_4O-), 71.04 (-OC_2H_4O-), 70.79 (-OC_2H_4O-), 70.70 (-OC_2H_4O-), 70.39 (-OC_2H_4O-), 66.63 (NCH_2CH_2O-), 65.93 (NCH_2CH_2O-), 56.16 (Ar-OCH_3), 53.53 (d, *J* = 3.0 Hz, NCH_3), 31.67 ($CH(CH_3)_2$), 31.41 ($CH(CH_3)_2$), 18.54 (d, *J* = 4.8 Hz, $CH(CH_3)_2$), 18.44 (d, *J* = 5.2 Hz, $CH(CH_3)_2$), 17.81 ($CH(CH_3)_2$), 17.68 ($CH(CH_3)_2$), 15.55 (-OCH_2CH_3). **^{31}P{^{1}H} NMR** (162 MHz, C_6D_6) δ 171.31. **IR** (THF, cm^{-1}): *v*(CO) 1930. **HRMS** (ESI^+) m/z: $[M + H]^+$ calcd for $C_{24}H_{41}IrNO_6P$ 664.2379; found 664.2325.

Synthesis of 5-((bis(2-methoxyethyl)amino)methyl)-2-methoxy phenol.

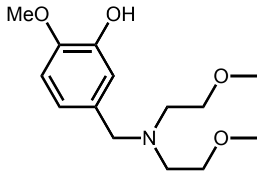

This compound was synthesized according to the literature procedure for the synthesis of 5-(aza-18-crown-6 methyl)-2-methoxy phenol.[2] To a stirring solution of 3-hydroxy-4-methoxybenzaldehyde (2.00 g, 13.1 mmol) in 100 mL of dry THF was added bis(2-methoxyethyl) amine (1.75 g, 13.2 mmol). The mixture was stirred for 30 min at room temperature and then sodium triacetoxyborohydride (3.92 g, 18.5 mmol) was added portion-wise over 1 hr. The reaction mixture was stirred for 2 days, and then quenched with 100 mL of aqueous saturated $NaHCO_3$ at room temperature. The mixture was transferred to a separatory funnel and extracted with CH_2Cl_2 (3 × 50 mL). The solvent of combined extracts was removed under vacuum to yield a white powder (931 mg, 26% yield), which was used in the next step without purification. **^{1}H NMR** (600 MHz, C_6D_6) δ 7.29 (d, *J* = 2.1 Hz, 1H, Ar-*H*), 6.90 (dd, *J* = 8.1, 2.0 Hz, 1H, Ar-*H*), 6.48 (d, *J* = 8.1 Hz, 1H, Ar-*H*), 5.61 (s, 1H, Ar-O*H*), 3.59 (s, 2H, ArCH_2N), 3.36 (t, *J* = 6.1 Hz, 4H, NCH_2CH_2OMe), 3.17 (s, 3H, Ar-OCH_3), 3.08 (s, 6H, $NCH_2CH_2OCH_3$), 2.77 (t, *J* = 6.1 Hz, 4H, NCH_2CH_2OMe).

$^{13}C\{^1H\}$ NMR (151 MHz, C_6D_6) δ 146.51 (Ar-*C*), 146.00 (Ar-*C*), 134.12 (Ar-*C*), 120.17 (Ar-*C*), 115.67 (Ar-*C*), 110.63 (Ar-*C*), 72.09 ($NCH_2CH_2OCH_3$), 59.92 ($ArCH_2N$), 58.50 ($Ar\text{-}OCH_3$), 55.36 ($NCH_2CH_2OCH_3$), 54.22 ($NCH_2CH_2OCH_3$). **HRMS** (ESI^+) m/z: $[M + H]^+$ calcd 270.1705; found 270.1697.

Synthesis of ($^{MeO\text{-}BME}$NCOP)H.

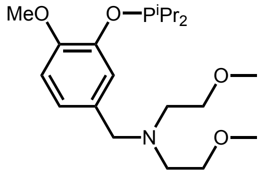

A flask was charged with 5-((bis(2-methoxyethyl)amino)methyl)-2-methoxy phenol (717 mg, 2.66 mmol) and 20 mL THF. To the clear, colorless stirred solution, $^iPr_2PCl$ (407 mg, 2.66 mmol) and NEt_3 (296 mg, 2.9 mmol) were sequentially added to the reaction mixture, resulting in a precipitate of a white solid. The reaction mixture was stirred overnight at room temperature. The volatiles were removed under vacuum leaving a mixture of an oil and white solid. The oil was dissolved in toluene and filtered through short plug of alumina. The toluene was removed under vacuum to yield a colorless oil of ($^{MeO\text{-}BME}$NCOP)H (779 mg, 56% yield). **^1H NMR** (600 MHz, C_6D_6) δ 7.65 (t, J = 2.4 Hz, 1H, Ar-*H*), 6.97 (dd, J = 8.1, 2.0 Hz, 1H, Ar-*H*), 6.61 (d, J = 8.2 Hz, 1H, Ar-*H*), 3.62 (s, 2H, $ArCH_2N$), 3.42 – 3.37 (m, 7H, NCH_2CH_2OMe and $Ar\text{-}OCH_3$), 3.11 (s, 6H, $NCH_2CH_2OCH_3$), 2.79 (t, J = 6.1 Hz, 4H, NCH_2CH_2OMe), 1.89 (heptd, J = 7.1, 2.6 Hz, 2H, $CH(CH_3)_2$), 1.29 (dd, J = 10.4, 7.0 Hz, 6H, $CH(CH_3)_2$), 1.07 (dd, J = 15.5, 7.2 Hz, 6H, $CH(CH_3)_2$). **$^{13}C\{^1H\}$ NMR** (151 MHz, C_6D_6) δ 150.15 (Ar-*C*), 149.18 (d, J = 8.2 Hz, Ar-*C*), 133.39 (Ar-*C*), 122.27 (Ar-*C*), 120.27 (d, J = 15.5 Hz, Ar-*C*), 112.49 (Ar-*C*), 72.17 ($NCH_2CH_2OCH_3$), 59.84 ($ArCH_2N$), 58.55 ($Ar\text{-}OCH_3$), 55.58 ($NCH_2CH_2OCH_3$), 54.25 ($NCH_2CH_2OCH_3$), 28.87 (d, J = 18.9 Hz, $CH(CH_3)_2$), 18.00 (d, J = 20.5 Hz, $CH(CH_3)_2$), 17.32 (d, J = 9.0 Hz, $CH(CH_3)_2$). **$^{31}P\{^1H\}$ NMR** (162 MHz, C_6D_6) δ 152.82. **HRMS** (ESI^+) m/z: $[M + H]^+$ calcd 386.2460; found 386.2450.

Synthesis of ($^{MeO-BME}$NCOP)Ir(H)(Cl) (1^{BME}).

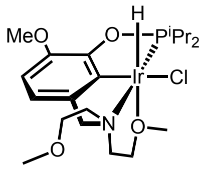

An orange mixture of ($^{MeO-BME}$NCOP)H (230 mg, 0.596 mmol) and [Ir(COD)Cl]$_2$ (200 mg, 0.298 mmol) in 10 mL toluene was stirred at 80 °C for overnight. The resulting red solution was cooled to room temperature and the volatiles were removed under vacuum. The residue was washed with ether and dried under vacuum to give a light yellow powder of **1^{BME}** (237 mg, 65% yield). Crystals suitable for X-ray diffraction were grown by layering a concentrated toluene solution of **1^{BME}** with pentane. **^{1}H NMR** (400 MHz, C_6D_6) δ 6.55 (d, *J* = 8.0 Hz, 1H, Ar-*H*), 6.44 (d, *J* = 8.1 Hz, 1H, Ar-*H*), 4.69 (d, *J* = 15.0 Hz, 1H, ArC*H*$_2$N), 3.91 (ddd, *J* = 10.8, 7.0, 3.5 Hz, 1H, NCH$_2$C*H*$_2$OMe), 3.74 (ddd, *J* = 13.6, 6.2, 3.5 Hz, 1H, NC*H*$_2$CH$_2$OMe), 3.67 – 3.54 (m, 5H, ArC*H*$_2$N, NCH$_2$C*H*$_2$OMe and Ar-OC*H*$_3$), 3.35 (ddt, *J* = 13.9, 7.3, 3.7 Hz, 1H, NC*H*$_2$CH$_2$OMe), 3.18 (s, 3H, NCH$_2$CH$_2$OC*H*$_3$), 3.00 (s, 5H, NCH$_2$C*H*$_2$OMe and NCH$_2$CH$_2$OC*H*$_3$), 2.63 – 2.54 (m, 1H, NC*H*$_2$CH$_2$OMe), 2.54 – 2.42 (m, 1H, C*H*(CH$_3$)$_2$), 2.27 – 2.10 (m, 2H, NC*H*$_2$CH$_2$OMe and C*H*(CH$_3$)$_2$), 1.40 (dd, *J* = 16.8, 7.5 Hz, 3H, CH(C*H*$_3$)$_2$), 1.31 (dd, *J* = 13.7, 7.0 Hz, 3H, CH(C*H*$_3$)$_2$), 1.21 (dd, *J* = 18.8, 6.9 Hz, 3H, CH(C*H*$_3$)$_2$), 0.96 (dd, *J* = 15.4, 7.0 Hz, 3H, CH(C*H*$_3$)$_2$), –30.49 (d, *J* = 26.3 Hz, 1H, Ir-H). **^{13}C{^{1}H} NMR** (151 MHz, C_6D_6) δ 152.11 (d, *J* = 4.4 Hz, Ar-*C*), 143.65 (d, *J* = 12.1 Hz, Ar-*C*), 139.52 (d, *J* = 3.2 Hz, Ar-*C*), 138.01 (t, *J* = 3.9 Hz, Ar-*C*), 114.57 (Ar-*C*), 108.21 (Ar-*C*), 72.83 (NCH$_2$*C*H$_2$OMe), 71.79 (NCH$_2$*C*H$_2$OMe), 71.31 (Ar*C*H$_2$N), 61.23 (NCH$_2$CH$_2$O*C*H$_3$), 60.91 (N*C*H$_2$CH$_2$OMe), 60.51 (N*C*H$_2$CH$_2$OMe), 58.45 (NCH$_2$CH$_2$O*C*H$_3$), 56.00 (Ar-O*C*H$_3$), 31.63 (d, *J* = 33.3 Hz, *C*H(CH$_3$)$_2$), 30.08 (d, *J* = 38.6 Hz, *C*H(CH$_3$)$_2$), 18.05 (d, *J* = 7.3 Hz, CH(*C*H$_3$)$_2$), 17.73 (CH(*C*H$_3$)$_2$), 17.08 (d, *J* = 4.2 Hz, CH(*C*H$_3$)$_2$), 16.56 (d, *J* = 3.3 Hz, CH(*C*H$_3$)$_2$). **^{31}P{^{1}H} NMR** (162 MHz, C_6D_6) δ 144.02 (d, *J* = 13.9 Hz). **HRMS** (ESI$^+$) m/z: [M – Cl$^-$]$^+$ calcd 578.2011; found 578.2009. **Anal. Calcd** for $C_{20}H_{36}ClIrNO_4P$: C, 39.18; H, 5.92; N, 2.28. Found: C, 39.25; H, 5.72; N, 2.17.

Decarbonylation of 1^{BME}.

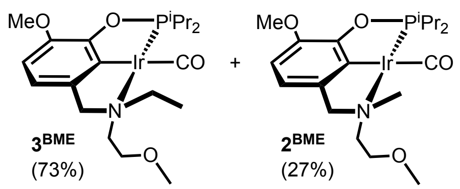

NaHMDS (9.0 mg, 0.049 mmol) was added to a solution of ($^{MeO\text{-}BME}$NCOP)Ir(H)(Cl) (**1^{BME}**, 30 mg, 0.049 mmol) in 10 mL toluene resulting in an immediate color change from yellow to orange. The reaction mixture was stirred at 80 °C for 3 hr. The solution was filtered through short plug of alumina and the filtrate was dried under vacuum. The formation of two carbonyl species **3^{BME}** and **2^{BME}** in 7:3 ratio was observed by ^{31}P NMR (δ 171.35 and 171.55). Separation of **3^{BME}** from **2^{BME}** was not successful; This product was not isolated. Characterization for **3^{BME}**; **^{1}H NMR** (600 MHz, C_6D_6) δ 6.74 (d, *J* = 8.0 Hz, 1H), 6.51 (d, *J* = 8.0 Hz, 1H), 4.33 (s, 2H), 4.18 – 4.10 (m, 1H), 4.01 – 3.94 (m, 1H), 3.55 – 3.45 (m, 4H), 3.36 – 3.26 (m, 1H), 3.20 – 3.07 (m, 2H), 3.02 (s, 3H), 2.21 – 2.02 (m, 2H), 1.30 (t, *J* = 7.1 Hz, 3H), 1.24 – 1.12 (m, 12H). **^{13}C{^{1}H} NMR** (101 MHz, C_6D_6) δ δ 198.13 (d, *J* = 2.7 Hz, Ir-*C*O), 168.60 (d, *J* = 7.7 Hz, Ar-*C*), 154.00 (d, *J* = 7.4 Hz, Ar-*C*), 146.26 (d, *J* = 2.5 Hz, Ar-*C*), 143.56 (d, *J* = 13.7 Hz, Ar-*C*), 115.47 (Ar-*C*), 112.31 (Ar-*C*), 73.06 (Ar*C*H_2N), 73.00 (NCH_2*C*H_2OMe), 65.05 (N*C*$H_2$$CH_2$OMe), 58.57 (N$CH_2$$CH_2$O*C*$H_3$), 56.12 (Ar-O*C*$H_3$), 55.62 (N*C*$H_2$$CH_3$) 31.48 (d, *J* = 37.9 Hz, *C*H(CH_3)$_2$), 18.46 (d, *J* = 5.0 Hz, CH(*C*H_3)$_2$), 17.72 (CH(*C*H_3)$_2$), 13.69 (NCH_2*C*H_3).**^{31}P{^{1}H} NMR** (243 MHz, C_6D_6) δ 171.35. **IR** (THF, cm^{-1}): *v*(CO) 1930. **HRMS** (ESI$^+$) m/z: [M]$^+$ calcd 575.1776; found 575.2012.

Characterization of the (κ^{4}-$^{MeO\text{-}BME}$NCOP)Ir.

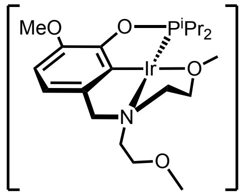

To a stirring solution of ($^{MeO\text{-}BME}$NCOP)Ir(H)(Cl) (**1^{BME}**, 10 mg, 0.016 mmol) in 0.5 mL C_6D_6 was added NaHMDS (3.0 mg, 0.016 mmol), resulting in an immediate color change from yellow to orange. The reaction mixture was transferred to an Teflon-sealed NMR tube and allowed to stand at room temperature for 5 h, during which time an intermediate (κ^{4}-$^{MeO\text{-}BME}$NCOP)Ir formed. (κ^{4}-$^{MeO\text{-}BME}$NCOP)Ir was stable at room temperature for ~2 d, during which time some **2^{BME}** and **3^{BME}**

were slowly generated. Spectroscopic data for (κ^{4}-$^{MeO\text{-}BME}$NCOP)Ir follows. **^{1}H NMR** (600 MHz, C_6D_6) δ 6.76 (d, *J* = 7.8 Hz, 1H, Ar-*H*), 6.58 (d, *J* = 7.9 Hz, 1H, Ar-*H*), 4.76 (d, *J* = 15.1 Hz, 1H, ArC*H*HN), 3.78 (dd, *J* = 15.2, 2.9 Hz, 1H, ArC*H*HN), 3.67 – 3.60 (m, 4H, Ar-OCH_3 and NCH$_2$CH_2OMe), 3.26 (ddd, *J* = 10.4, 6.2, 2.8 Hz, 1H, NCH$_2$CH_2OMe), 3.19 (ddd, *J* = 13.8, 5.8, 2.6 Hz, 1H, NCH_2CH$_2$OMe), 2.95 – 2.86 (m, 4H, NCH$_2$CH$_2$OCH_3 and NCH_2CH$_2$OMe), 2.85 – 2.76 (m, 2H, NCH$_2$CH_2OMe), 2.74 (s, 3H, NCH$_2$CH$_2$OCH_3), 2.62 (ddd, *J* = 13.2, 9.4, 3.3 Hz, 1H, NCH_2CH$_2$OMe), 2.49 – 2.39 (m, 1H, C*H*(CH$_3$)$_3$), 2.30 – 2.21 (m, 1H, C*H*(CH$_3$)$_3$), 2.02 (dt, *J* = 13.3, 2.6 Hz, 1H, NCH_2CH$_2$OMe), 1.25 (dd, *J* = 12.8, 7.0 Hz, 3H, CH(CH_3)$_3$), 1.09 (dd, *J* = 17.2, 7.5 Hz, 3H, CH(CH_3)$_3$), 1.00 (dd, *J* = 14.9, 6.9 Hz, 3H, CH(CH_3)$_3$), 0.93 (dd, *J* = 18.1, 6.9 Hz, 3H, CH(CH_3)$_3$). **^{13}C{^{1}H} NMR** (151 MHz, C_6D_6) δ 159.79 (d, *J* = 4.9 Hz, Ar-*C*), 150.77 (d, *J* = 4.8 Hz, Ar-*C*), 143.62 (d, *J* = 12.5 Hz, Ar-*C*), 142.46 (d, *J* = 3.0 Hz, Ar-*C*), 114.15 (Ar-*C*), 108.99 (Ar-*C*), 74.13 (Ar*C*H$_2$N), 72.52 (NCH$_2$*C*H$_2$OMe), 71.36 (NCH$_2$*C*H$_2$OMe), 62.74 (N*C*H$_2$CH$_2$OMe), 62.20 (NCH$_2$CH$_2$O*C*H$_3$), 61.07 (N*C*H$_2$CH$_2$OMe), 58.30 (NCH$_2$CH$_2$O*C*H$_3$), 56.11 (Ar-O*C*H$_3$), 31.76 (d, *J* = 26.4 Hz, *C*H(CH$_3$)$_3$), 31.52 (d, *J* = 34.1 Hz, *C*H(CH$_3$)$_3$), 18.44 (CH(*C*H$_3$)$_3$), 18.34 (d, *J* = 6.4 Hz, CH(*C*H$_3$)$_3$), 18.09 (d, *J* = 6.7 Hz, CH(*C*H$_3$)$_3$), 16.93 (d, *J* = 4.7 Hz, CH(*C*H$_3$)$_3$). **^{31}P{^{1}H} NMR** (243 MHz, C_6D_6) δ 144.46. **HRMS** (ESI$^+$) m/z: [M + H]$^+$ calcd 578.2011; found 578.1994.

Synthesis of 5-((diethylamino)methyl)-2-methoxy phenol.

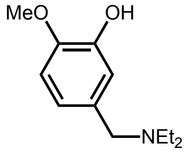

This compound was synthesized according to the literature procedure for the synthesis of 5-(aza-18-crown-6 methyl)-2-methoxy phenol.[2] To a solution of 3-hydroxy-4-methoxybenzaldehyde (2.000 g, 13.14 mmol) in 100 mL of dry THF was added diethylamine (1.5 mL, 1.1 g, 14 mmol) using a syringe. The mixture was stirred for 30 min at room temperature and then sodium triacetoxyborohydride (3.92 g, 18.5 mmol) was added portion-wise over 1 hr. The reaction mixture was stirred for 2 days, and then quenched with 100 mL of aqueous saturated $NaHCO_3$ at room temperature. The mixture was transferred to a separatory funnel and extracted with CH_2Cl_2 (3 × 50 mL). The solvent of extracts was removed under vacuum to yield a white powder (2.402 g, 87% yield), which was used in the next step without purification. **^{1}H NMR** (600 MHz, C_6D_6) δ 7.22 (d,

J = 2.1 Hz, 1H, Ar-*H*), 6.89 (dd, *J* = 8.2, 2.1 Hz, 1H, Ar-*H*), 6.53 (d, *J* = 8.2 Hz, 1H, Ar-*H*), 6.40 (s, 1H, Ar-O*H*), 3.43 (s, 2H, ArCH_2N), 3.27 (s, 3H, Ar-OCH_3), 2.44 (q, *J* = 7.1 Hz, 4H, NCH_2CH$_3$), 0.94 (t, *J* = 7.1 Hz, 6H, NCH$_2$CH_3). **^{13}C{^{1}H} NMR** (151 MHz, C_6D_6) δ 146.74 (Ar-*C*), 146.45 (Ar-*C*), 133.07 (Ar-*C*), 120.41 (Ar-*C*), 116.40 (Ar-*C*), 111.05 (Ar-*C*), 57.49 (Ar*C*H$_2$N), 55.48 (Ar-O*C*H$_3$), 46.68 (N*C*H$_2$CH$_3$), 11.73 (NCH$_2$*C*H$_3$). **HRMS** (ESI$^+$) m/z: [M + H]$^+$ calcd 210.1494; found 210.1489.

Synthesis of ($^{\text{MeO-Et}}$NCOP)H.

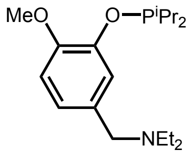

A flask was charged with 5-((diethylamino)methyl)-2-methoxy phenol (2.402 g, 11.48 mmol) and 20 mL THF. To the clear, colorless stirred solution, ^{i}Pr$_2$PCl (1.752 g, 11.48 mmol) and NEt$_3$ (1.28 g, 12.7 mmol) were sequentially added to a reaction mixture, resulting in a precipitate of a white solid. The reaction mixture was stirred overnight at room temperature. The volatiles were removed under vacuum leaving a mixture of an oil and white solid. The oil was extracted with toluene and filtered through short plug of alumina. The toluene was removed under vacuum to yield a colorless oil of ($^{\text{MeO-Et}}$NCOP)H (2.368 g, 63% yield). **^{1}H NMR** (400 MHz, C_6D_6) δ 7.65 (s, 1H, Ar-*H*), 6.99 (d, *J* = 8.2 Hz, 1H, Ar-*H*), 6.64 (d, *J* = 8.2 Hz, 1H, Ar-*H*), 3.46 (s, 2H, ArCH_2N), 3.42 (s, 3H, Ar-OCH_3), 2.45 (q, *J* = 7.1 Hz, 4H, NCH_2CH$_3$), 1.96 – 1.81 (m, 2H, C*H*(CH$_3$)$_2$), 1.29 (dd, *J* = 10.5, 6.9 Hz, 6H, CH(CH_3)$_2$), 1.07 (dd, *J* = 15.5, 7.3 Hz, 6H, CH(CH_3)$_2$), 0.98 (t, *J* = 7.1 Hz, 6H, NCH$_2$CH_3). **^{13}C{^{1}H} NMR** (151 MHz, C_6D_6) δ 150.09 (Ar-*C*), 149.23 (d, *J* = 8.2 Hz, Ar-*C*), 133.65 (Ar-*C*), 122.19 (Ar-*C*), 120.29 (d, *J* = 15.6 Hz, Ar-*C*), 112.68 (Ar-*C*), 57.77 (Ar*C*H$_2$N), 55.69 (Ar-O*C*H$_3$), 47.05 (N*C*H$_2$CH$_3$), 28.90 (d, *J* = 19.0 Hz, *C*H(CH$_3$)$_2$), 18.01 (d, *J* = 20.7 Hz, CH(*C*H$_3$)$_2$), 17.33 (d, *J* = 8.7 Hz, CH(*C*H$_3$)$_2$), 12.39 (NCH$_2$*C*H$_3$). **^{31}P{^{1}H} NMR** (162 MHz, C_6D_6) δ 152.77. **HRMS** (ESI$^+$) m/z: [M + H]$^+$ calcd 326.2249; found 326.2241.

Synthesis of ($^{MeO-Et}$NCOP)Ir(H)(Cl) (1^{Et}).

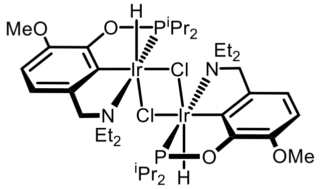

An orange mixture of ($^{MeO-Et}$NCOP)H (519 mg, 1.59 mmol) and $[Ir(COD)Cl]_2$ (536 mg, 0.797 mmol) in 10 mL toluene was stirred at 80 °C overnight. The solution was cooled to room temperature resulting in a precipitation of yellow solid. The solid was gathered on a frit, washed with ether and dried under vacuum to give yellow powder of **1^{Et}** (537 mg, 61% yield). Crystals suitable for X-ray diffraction were grown by slow evaporation of CH_2Cl_2/pentane solution of **1^{Et}**. **^{1}H NMR** (500 MHz, C_6D_6) δ 6.58 (d, *J* = 8.0 Hz, 1H, Ar-*H*), 6.47 (d, *J* = 8.1 Hz, 1H, Ar-*H*), 4.04 (d, *J* = 15.4 Hz, 1H, ArC*H*$_2$N), 3.58 – 3.38 (m, 5H, Ar-OC*H*$_3$, ArC*H*$_2$N and NC*H*$_2$CH$_3$), 3.35 – 3.23 (m, 1H, NC*H*$_2$CH$_3$), 3.10 – 2.99 (m, 1H, NC*H*$_2$CH$_3$), 2.85 – 2.73 (m, 1H, NC*H*$_2$CH$_3$), 2.47 – 2.36 (m, 1H, C*H*(CH$_3$)$_2$), 2.03 – 1.90 (m, 1H, C*H*(CH$_3$)$_2$), 1.29 (dd, *J* = 17.5, 7.1 Hz, 3H, CH(C*H*$_3$)$_2$), 1.13 (dd, *J* = 14.6, 7.1 Hz, 3H, CH(C*H*$_3$)$_2$), 1.09 – 0.99 (m, 6H, CH(C*H*$_3$)$_2$ and NCH$_2$C*H*$_3$), 0.93 (dd, *J* = 15.8, 7.0 Hz, 3H, CH(C*H*$_3$)$_2$), 0.68 (t, *J* = 7.3 Hz, 3H, NCH$_2$C*H*$_3$), –39.01 (d, *J* = 24.3 Hz, 1H). **^{13}C{^{1}H} NMR** (151 MHz, C_6D_6) δ 152.94 (Ar-*C*), 143.66 (d, *J* = 12.1 Hz, Ar-*C*), 141.36 (Ar-*C*), 137.00 (Ar-*C*), 114.89 (Ar-*C*), 109.03 (Ar-*C*), 67.25 (N*C*H$_2$CH$_3$), 65.93 (N*C*H$_2$CH$_3$), 56.01 (Ar*C*H$_2$N), 54.93 (N*C*H$_2$CH$_3$), 30.57 (d, *J* = 34.7 Hz, *C*H(CH$_3$)$_2$), 30.12 (d, *J* = 38.7 Hz, *C*H(CH$_3$)$_2$), 17.37 (d, *J* = 3.3 Hz, CH(*C*H$_3$)$_2$), 17.32 (CH(*C*H$_3$)$_2$), 17.27 (d, *J* = 2.3 Hz, CH(*C*H$_3$)$_2$), 17.16 (CH(*C*H$_3$)$_2$), 11.76 (NCH$_2$*C*H$_3$), 11.43 (NCH$_2$*C*H$_3$). **^{31}P{^{1}H} NMR** (202 MHz, C_6D_6) δ 147.44. **HRMS** (ESI$^+$) m/z: $[M - Cl^- + CH_3CN]^+$ calcd 559.2065; found 559.2062. **Anal. Calcd** for $C_{18}H_{32}ClIrNO_2P$: C, 39.09; H, 5.83; N, 2.53. Found: C, 38.58; H, 5.81; N, 2.42.

Synthesis of {($^{MeO-Et}$NCOP)Ir}$_2$(N_2) (4).

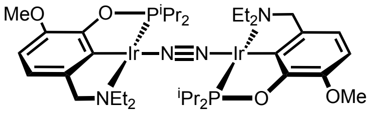

To a stirring suspension of ($^{Me-OEt}$NCOP)Ir(H)(Cl) (**1^{Et}**, 250 mg, 0.452 mmol) in 10 mL benzene was added NaHMDS (83 mg, 0.45 mmol), resulting in an immediate color change from yellow to deep red. The reaction mixture was stirred for 1 h at room temperature. The precipitate was filtered off and the filtrate was removed under vacuum. The residue was washed with pentane to give red

powder of **4** (134 mg, 56% yield). **^{1}H NMR** (500 MHz, C_6D_6) δ 6.74 (d, *J* = 8.0 Hz, 1H, Ar-*H*), 6.57 (d, *J* = 8.0 Hz, 1H, Ar-*H*), 3.85 (s, 2H, ArC*H*$_2$N), 3.64 (s, 3H, Ar-OC*H*$_3$), 3.01 – 2.89 (m, 4H, NC*H*$_2$CH$_3$), 2.24 – 2.15 (m, 2H, C*H*(CH$_3$)$_2$), 1.38 (t, *J* = 7.1 Hz, 6H, NCH$_2$C*H*$_3$), 1.32 (dd, *J* = 14.1, 6.9 Hz, 6H, CH(C*H*$_3$)$_2$), 1.26 (dd, *J* = 17.2, 7.1 Hz, 6H, CH(C*H*$_3$)$_2$). **^{13}C{^{1}H} NMR** (151 MHz, C_6D_6) δ 154.90 (Ar-*C*), 154.89 (d, *J* = 15.0 Hz, Ar-*C*), 144.96 (Ar-*C*), 142.84 (d, *J* = 12.4 Hz, Ar-*C*), 114.70 (Ar-*C*), 110.28 (Ar-*C*), 68.07 (N*C*H$_2$CH$_3$), 56.87 (Ar*C*H$_2$N), 56.14 (Ar-O*C*H$_3$), 31.55 (d, *J* = 34.9 Hz, *C*H(CH$_3$)$_2$), 18.89 (d, *J* = 6.0 Hz, CH(*C*H$_3$)$_2$), 17.95 (CH(*C*H$_3$)$_2$), 12.80 (NCH$_2$*C*H$_3$). **^{31}P{^{1}H} NMR** (202 MHz, C_6D_6) δ 163.68. **rRaman** (solid, 298K, 633 nm excitation, cm^{-1}): 2017. **HRMS** (ESI$^+$) m/z: [M]$^+$ calcd 1060.3481; found 1060.3590.

Synthesis of ($^{\text{MeO-Et}}$NCOP)Ir(norbornene) (5).

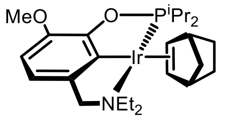

To a stirring suspension of ($^{\text{Me-OEt}}$NCOP)Ir(H)(Cl) (**1^{Et}**, 100 mg, 0.181 mmol) in 10 mL benzene was added norbornene (30 mg, 0.32 mmol). To this reaction mixture was added NaHMDS (34 mg, 0.19 mmol), resulting in an immediate color change from yellow to orange. The reaction mixture was stirred for 1 h at room temperature. A precipitate was filtered off and the filtrate was dried under vacuum. The residue was washed with cold pentane (–35 °C) to give orange powder of **5** (99 mg, 90%). **^{1}H NMR** (600 MHz, C_6D_6) δ 6.78 (d, *J* = 7.9 Hz, 1H, Ar-*H*), 6.70 (d, *J* = 7.9 Hz, 1H, Ar-*H*), 3.83 (s, 2H, ArC*H*$_2$N), 3.65 (s, 3H, Ar-OC*H*$_3$), 3.16 – 3.06 (m, 2H, norbornene), 3.03 – 2.91 (m, 4H, NC*H*$_2$CH$_3$), 2.70 (s, 2H, norbornene), 2.53 – 2.40 (m, 2H, *C*H(CH$_3$)$_2$), 1.59 (d, *J* = 6.9 Hz, 2H, norbornene), 1.42 (d, *J* = 8.3 Hz, 1H, norbornene), 1.22 – 1.14 (m, 12H, CH(C*H*$_3$)$_2$), 1.09 (t, *J* = 7.1 Hz, 6H, NCH$_2$C*H*$_3$), 0.35 (d, *J* = 8.2 Hz, 1H, norbornene). **^{13}C{^{1}H} NMR** (151 MHz, C_6D_6) δ 164.06 (d, *J* = 8.8 Hz, Ar-*C*), 150.20 (d, *J* = 10.8 Hz, Ar-*C*), 144.04 (d, *J* = 15.9 Hz, Ar-*C*), 141.23 (Ar-*C*), 114.43 (Ar-*C*), 108.02 (Ar-*C*), 70.13 (N*C*H$_2$CH$_3$), 62.68 (d, *J* = 2.5 Hz, norbornene), 57.08 (Ar*C*H$_2$N), 56.43 (Ar-O*C*H$_3$), 45.13 (norbornene), 40.14 (norbornene), 30.66 (d, *J* = 38.1 Hz, *C*H(CH$_3$)$_2$), 29.48 (norbornene), 18.79 (d, *J* = 2.6 Hz, CH(*C*H$_3$)$_2$), 17.42 (d, *J* = 1.7 Hz, CH(*C*H$_3$)$_2$), 12.35 (norbornene). **^{31}P{^{1}H} NMR** (243 MHz, C_6D_6) δ 157.88. **HRMS** (ESI$^+$) m/z: [M – norbornene + MeCN + H]$^+$ calcd 559.2065; found 559.2062.

II. NMR Spectra of New Compounds

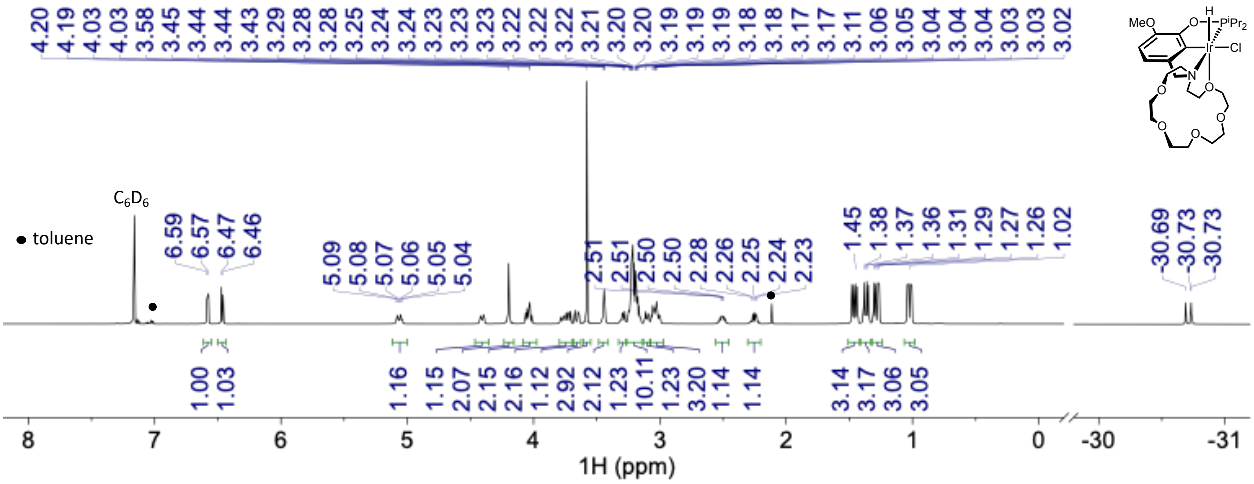

Figure S1. ^{1}H NMR spectrum of $\mathbf{1}^{\mathbf{18c6}}$ (600 MHz, C_6D_6).

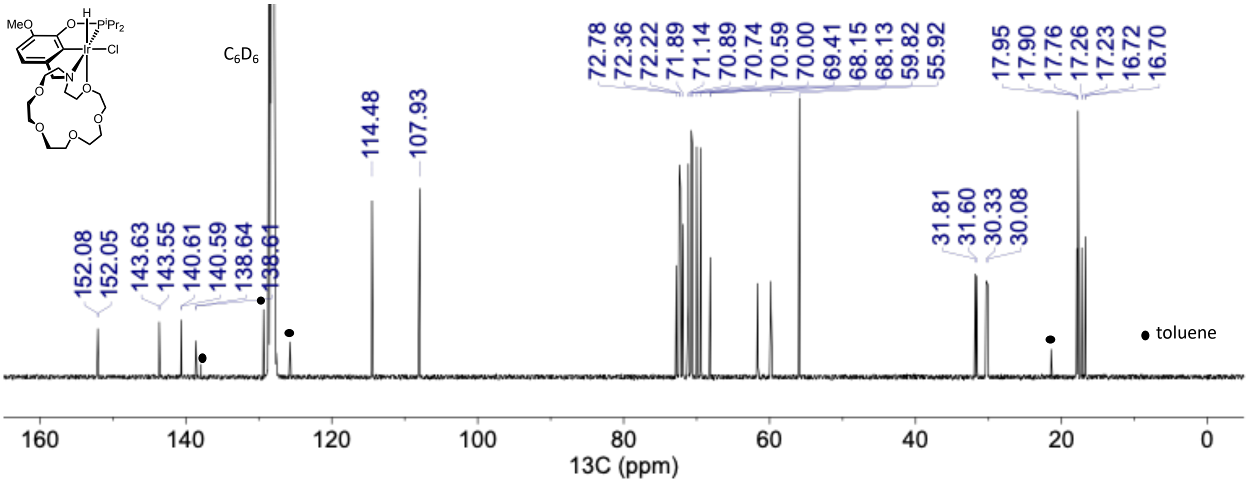

Figure S2. $^{13}C\{^{1}H\}$ NMR spectrum of $\mathbf{1}^{\mathbf{18c6}}$ (151 MHz, C_6D_6).

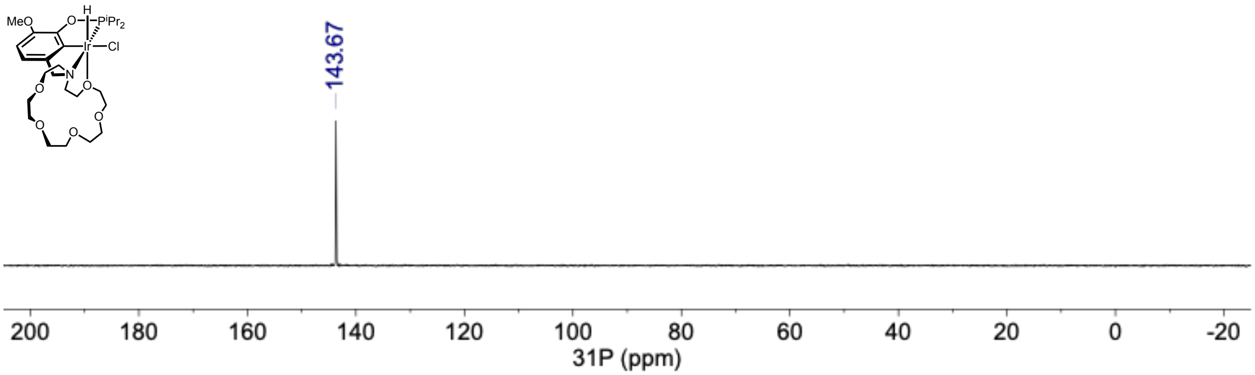

Figure S3. $^{31}P\{^{1}H\}$ NMR spectrum of $\mathbf{1}^{\mathbf{18c6}}$ (162 MHz, C_6D_6).

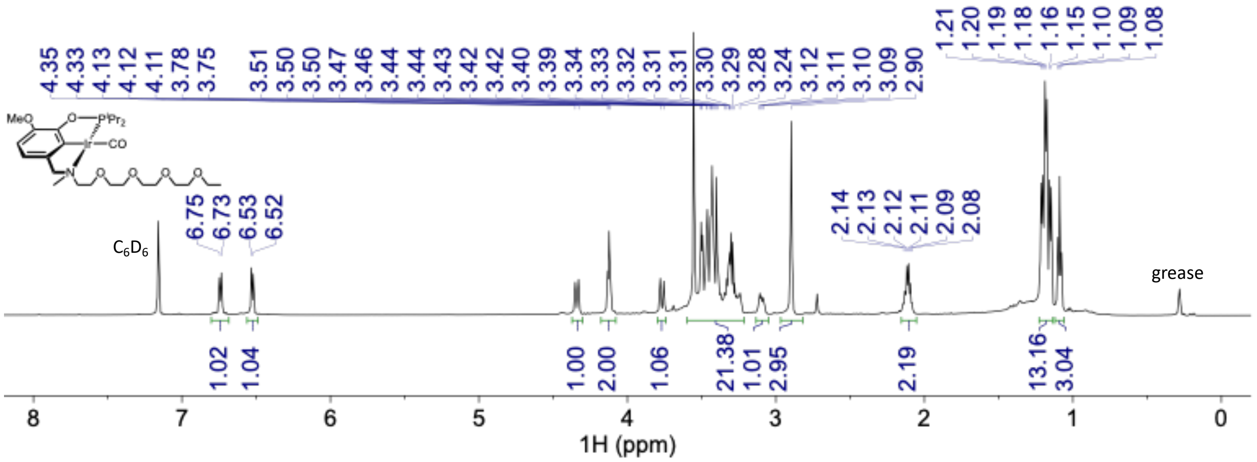

Figure S4. ^{1}H NMR spectrum of $\mathbf{2^{18c6}}$ (600 MHz, C_6D_6).

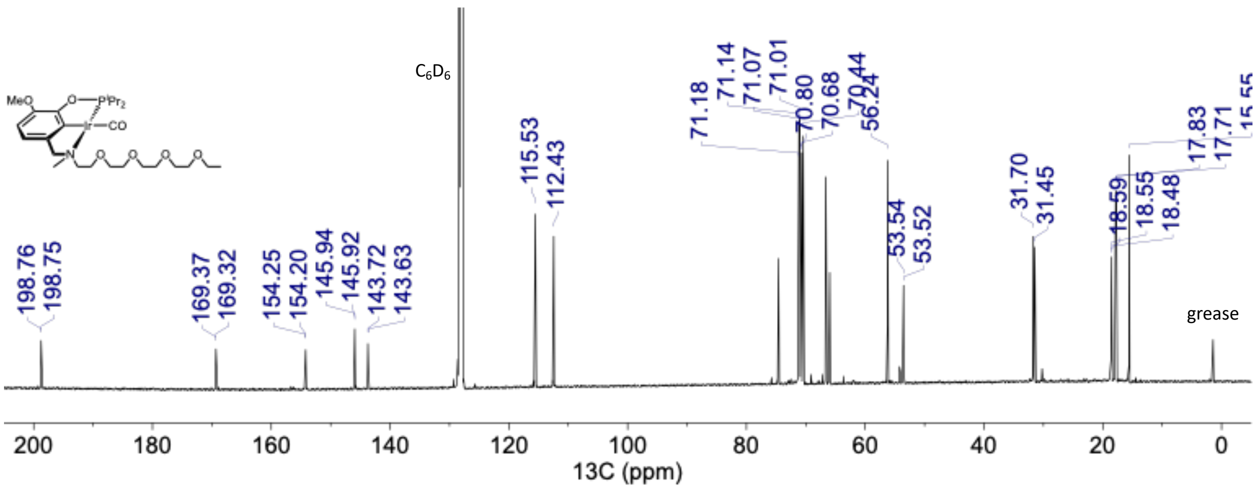

Figure S5. $^{13}C\{^{1}H\}$ NMR spectrum of $\mathbf{2^{18c6}}$ (151 MHz, C_6D_6).

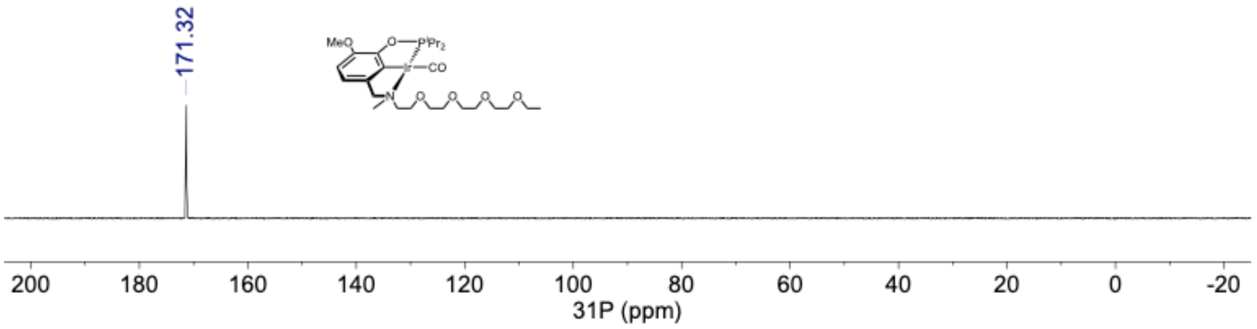

Figure S6. $^{31}P\{^{1}H\}$ NMR spectrum of $\mathbf{2^{18c6}}$ (162 MHz, C_6D_6).

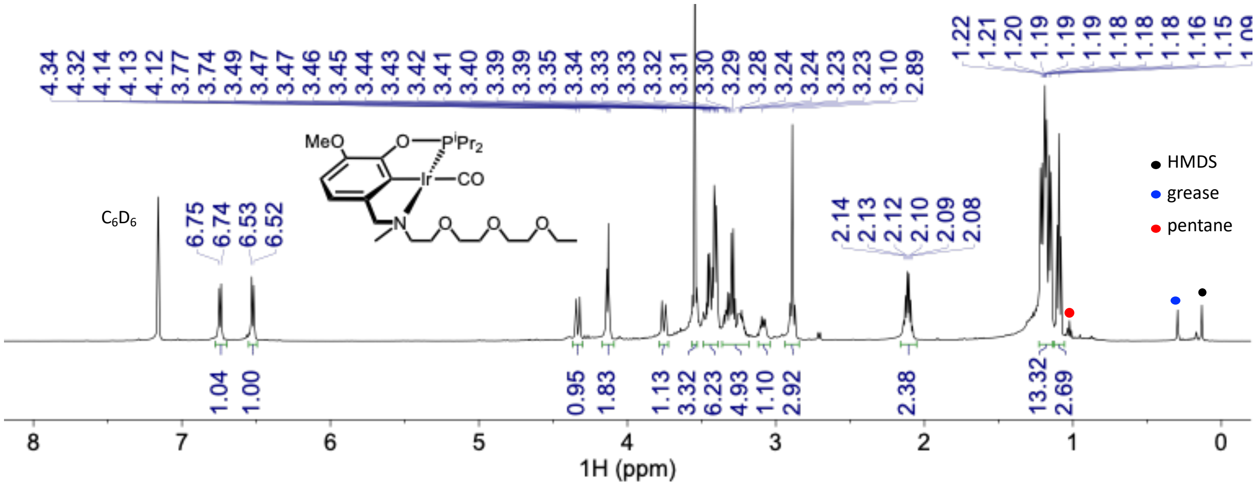

Figure S7. ^{1}H NMR spectrum of $\mathbf{2^{15c5}}$ (600 MHz, C_6D_6).

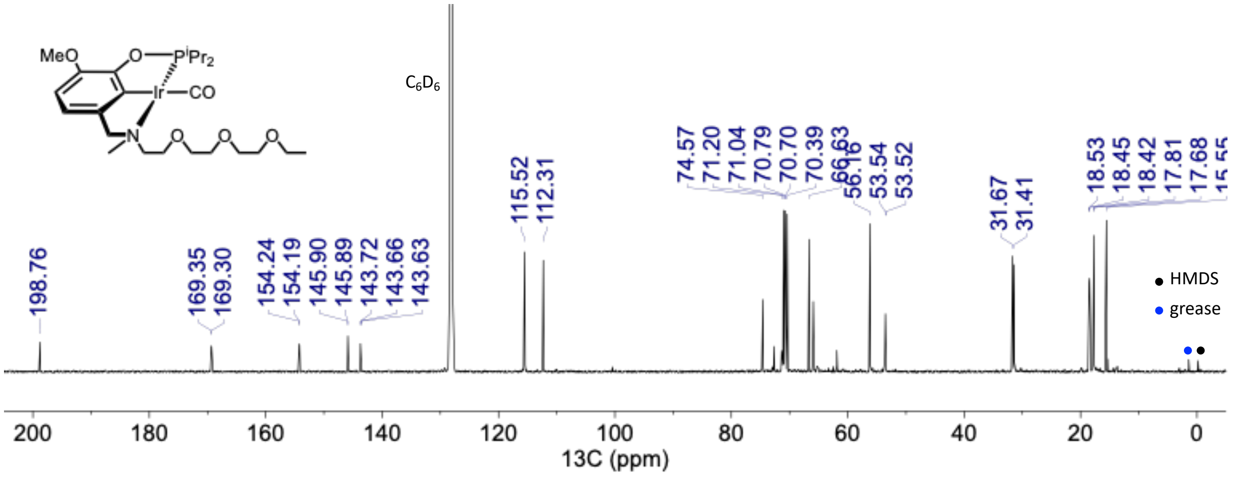

Figure S8. $^{13}C\{^{1}H\}$ NMR spectrum of $\mathbf{2^{15c5}}$ (151 MHz, C_6D_6).

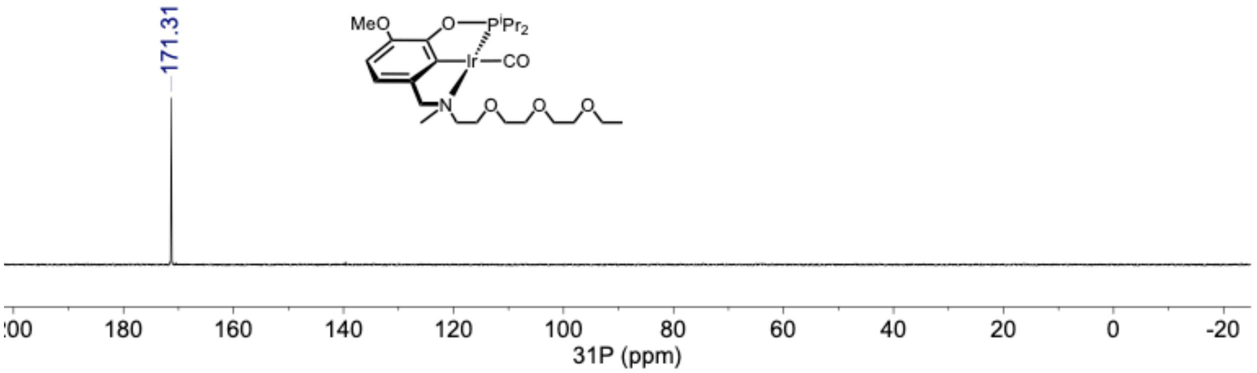

Figure S9. $^{31}P\{^{1}H\}$ NMR spectrum of $\mathbf{2^{15c5}}$ (162 MHz, C_6D_6).

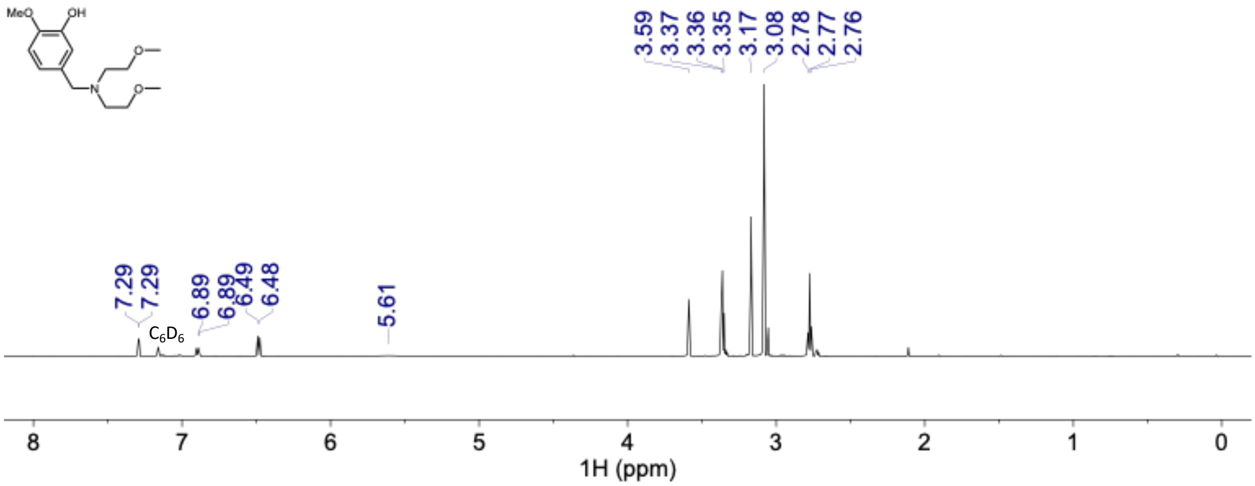

Figure S10. ^{1}H NMR spectrum of 5-((bis(2-methoxyethyl)amino)methyl)-2-methoxy phenol (600 MHz, C_6D_6).

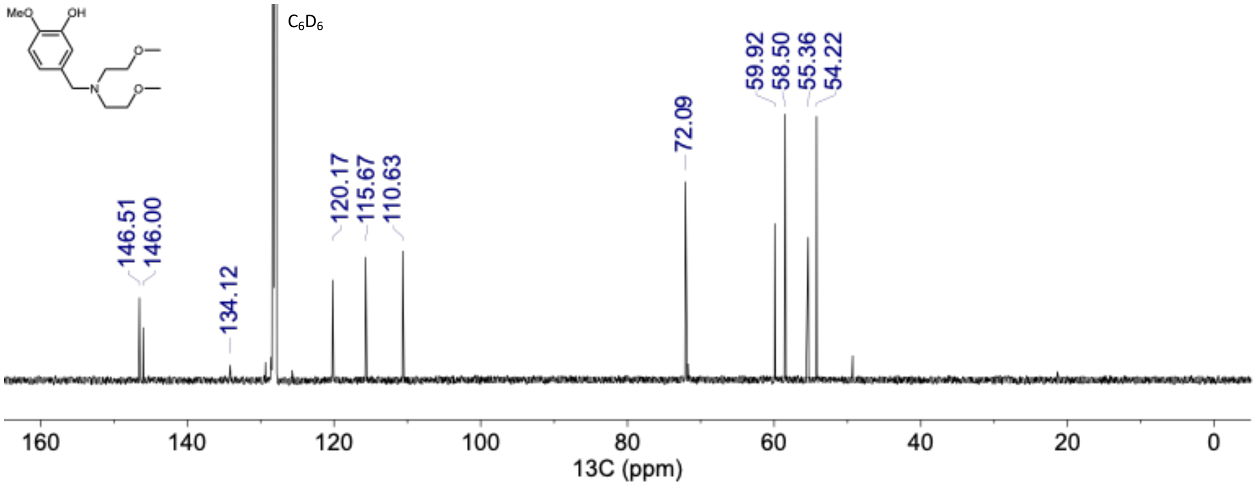

Figure S11. $^{13}C\{^{1}H\}$ NMR spectrum of 5-((bis(2-methoxyethyl)amino)methyl)-2-methoxy phenol (151 MHz, C_6D_6).

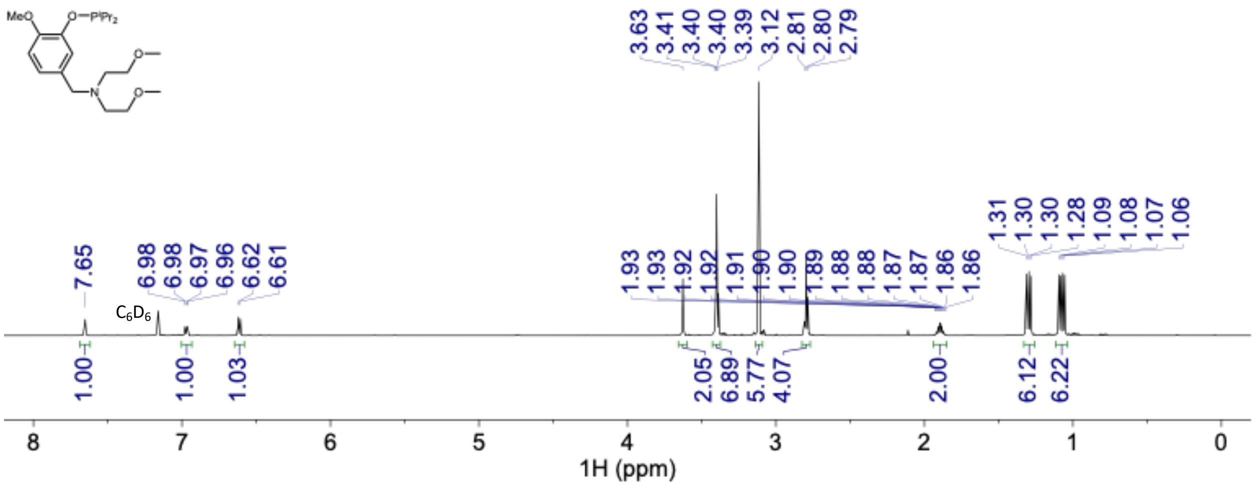

Figure S12. ^{1}H NMR spectrum of ($^{MeO\text{-}BME}$NCOP)H (600 MHz, C_6D_6).

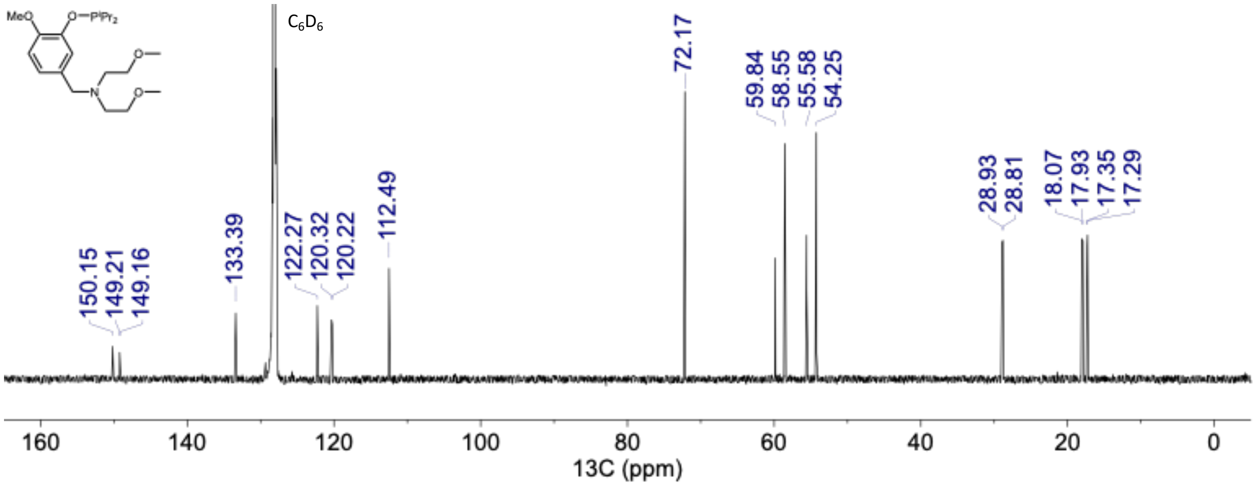

Figure S13. ^{13}C{^{1}H} NMR spectrum of ($^{MeO\text{-}BME}$NCOP)H (151 MHz, C_6D_6).

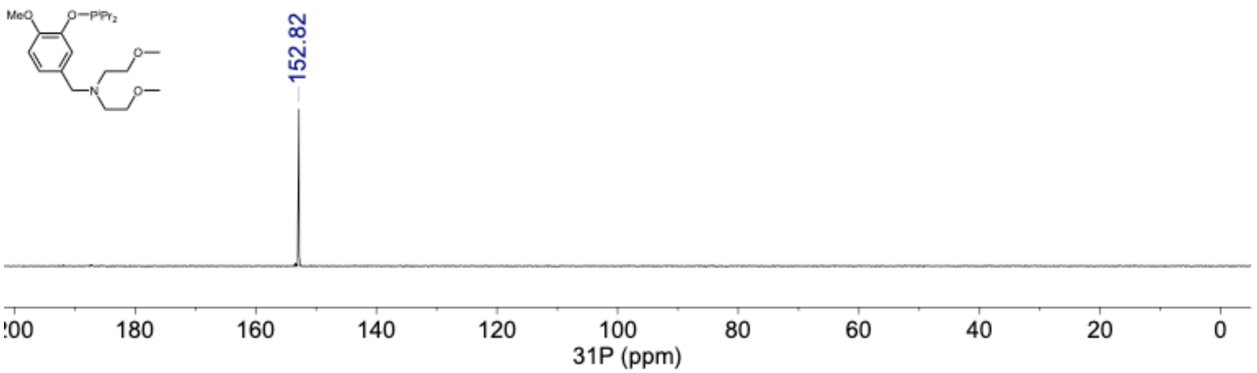

Figure S14. ^{31}P{^{1}H} NMR spectrum of ($^{MeO\text{-}BME}$NCOP)H (162 MHz, C_6D_6).

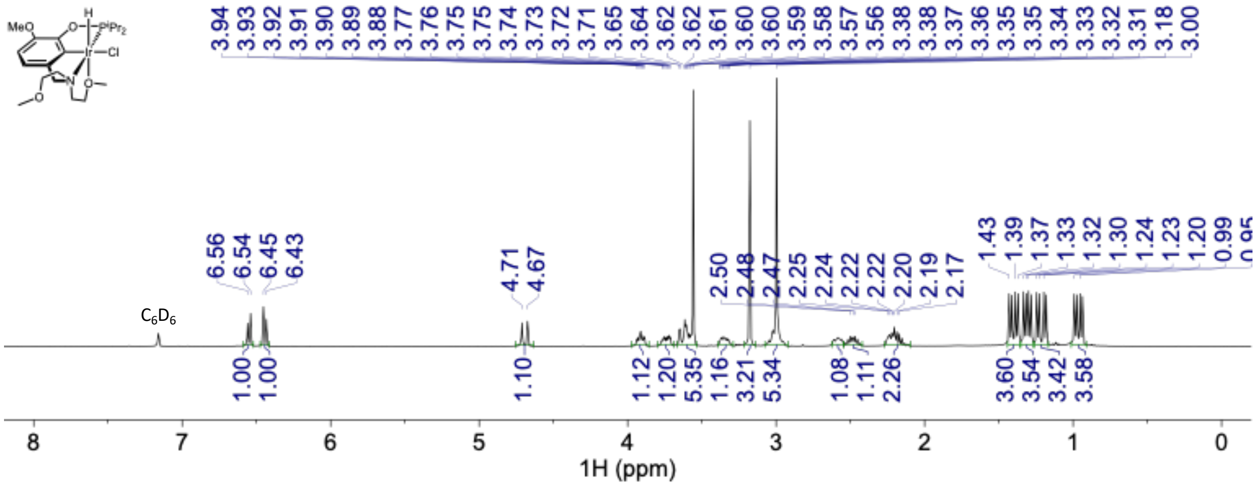

Figure S15. ^{1}H NMR spectrum of $\mathbf{1^{BME}}$ (600 MHz, C_6D_6).

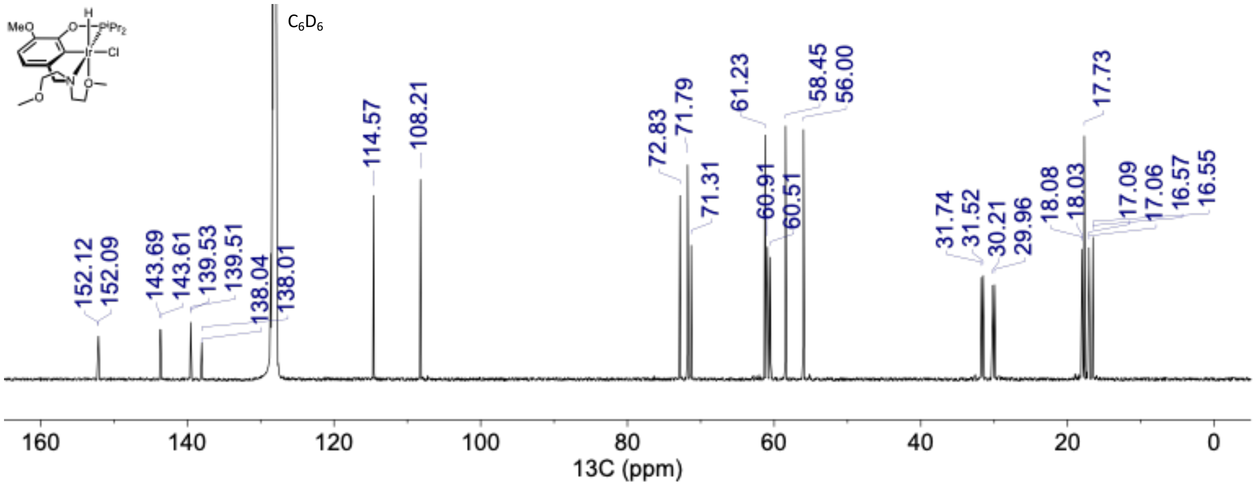

Figure S16. ^{13}C{^{1}H} NMR spectrum of $\mathbf{1^{BME}}$ (151 MHz, C_6D_6).

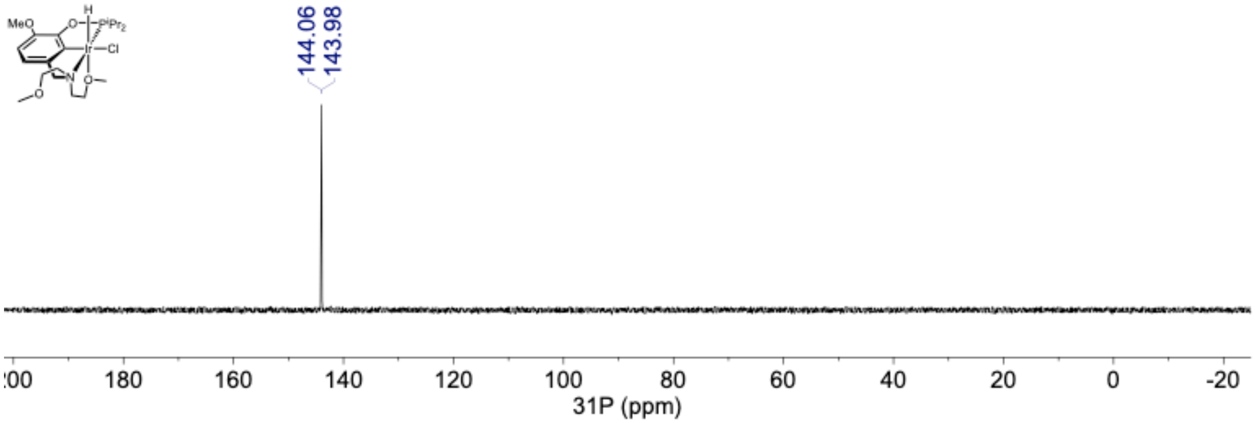

Figure S17. ^{31}P{^{1}H} NMR spectrum of $\mathbf{1^{BME}}$ (162 MHz, C_6D_6).

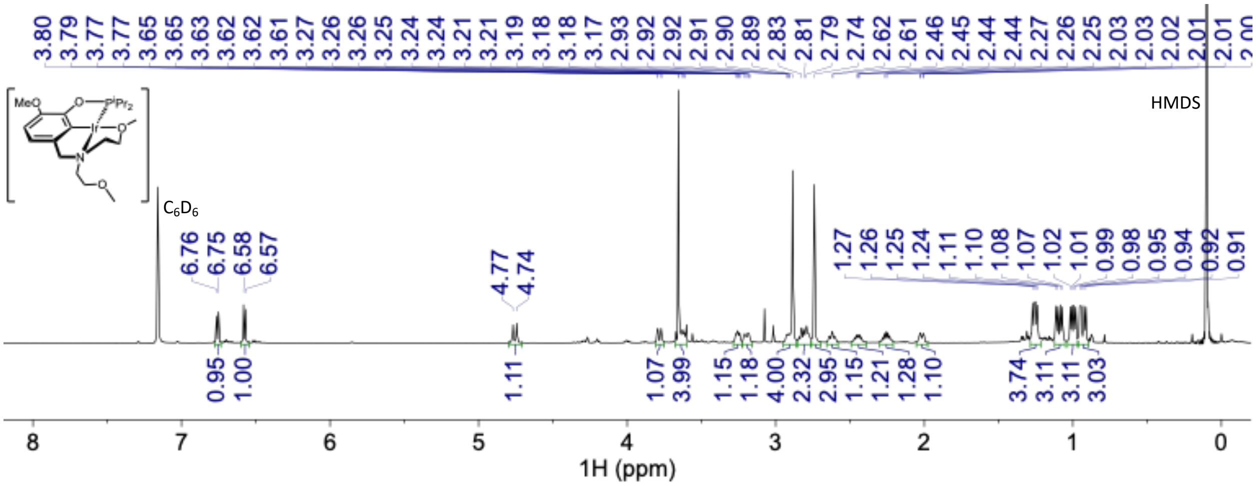

Figure S18. ^{1}H NMR spectrum of (κ^{4}-$^{MeO\text{-}BME}$NCOP)Ir (600 MHz, C_6D_6).

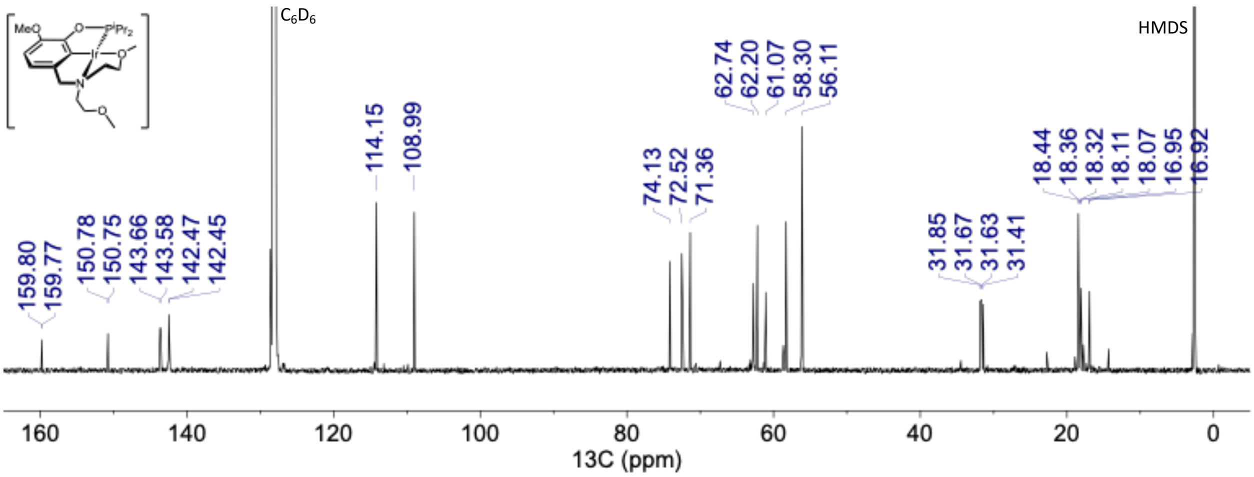

Figure S19. $^{13}C\{^{1}H\}$ NMR spectrum of (κ^{4}-$^{MeO\text{-}BME}$NCOP)Ir (151 MHz, C_6D_6).

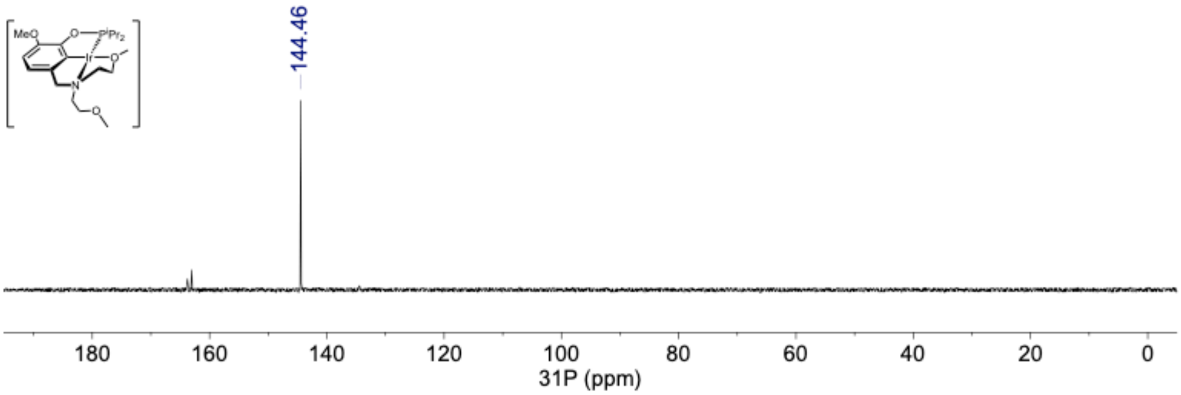

Figure S20. $^{31}P\{^{1}H\}$ NMR spectrum of (κ^{4}-$^{MeO\text{-}BME}$NCOP)Ir (243 MHz, C_6D_6).

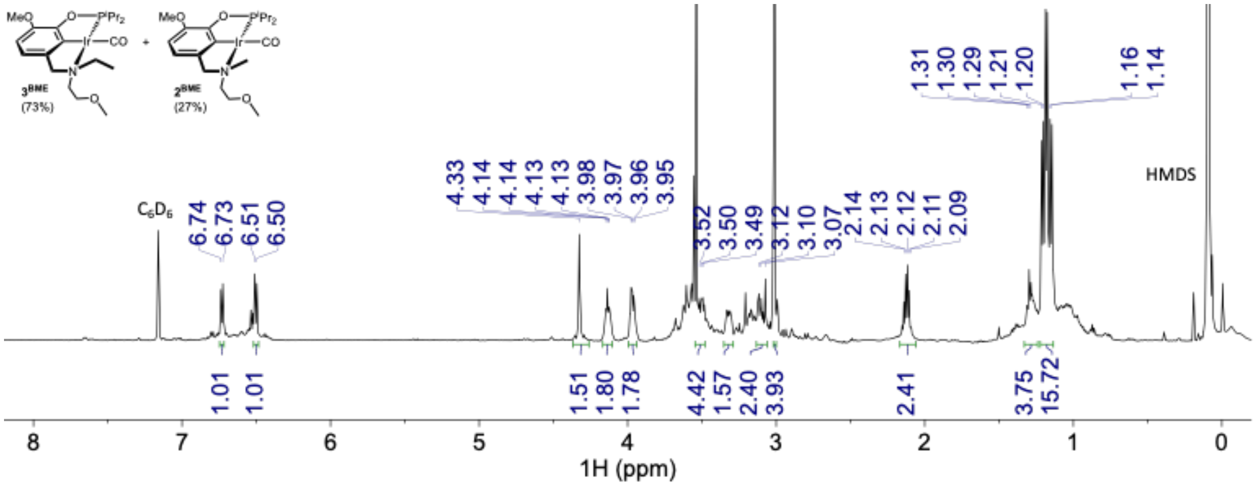

Figure S21. ^{1}H NMR spectrum of the mixture of $\mathbf{3^{BME}}$ and $\mathbf{2^{BME}}$ (600 MHz, C_6D_6). Only the major product $\mathbf{3^{BME}}$ was assigned.

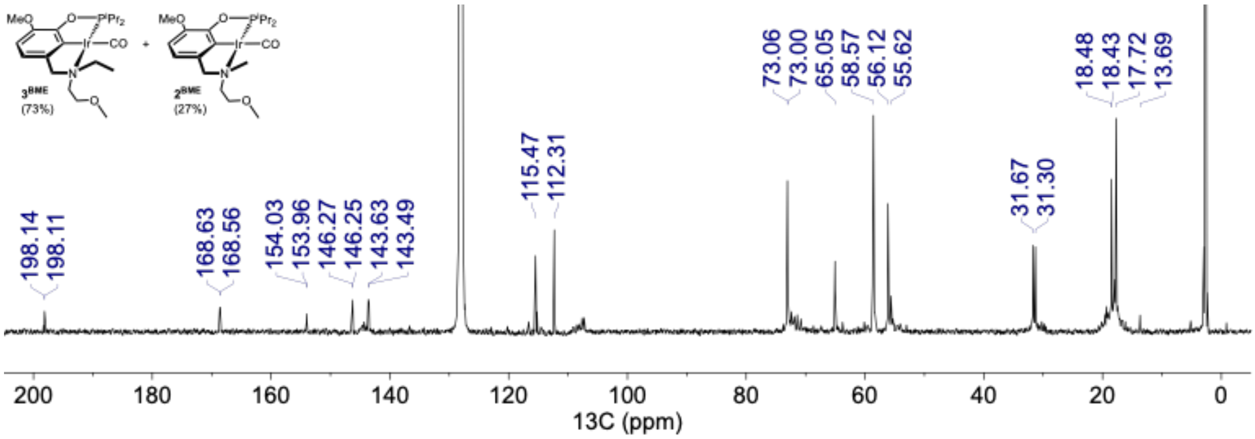

Figure S22. $^{13}C\{^{1}H\}$ NMR spectrum of the mixture of $\mathbf{3^{BME}}$ and $\mathbf{2^{BME}}$ (101 MHz, C_6D_6). Only the major product $\mathbf{3^{BME}}$ was assigned.

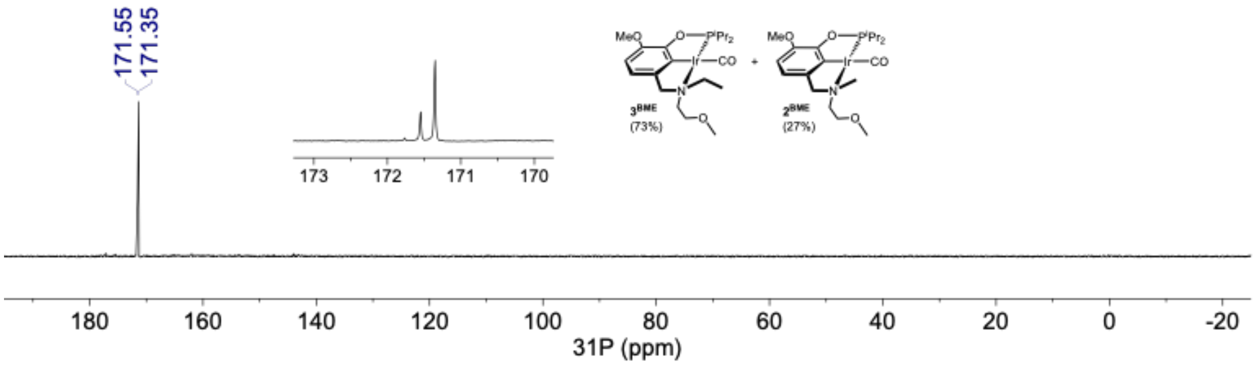

Figure S23. $^{31}P\{^{1}H\}$ NMR spectrum of the mixture of $\mathbf{3^{BME}}$ and $\mathbf{2^{BME}}$ (162 MHz, C_6D_6).

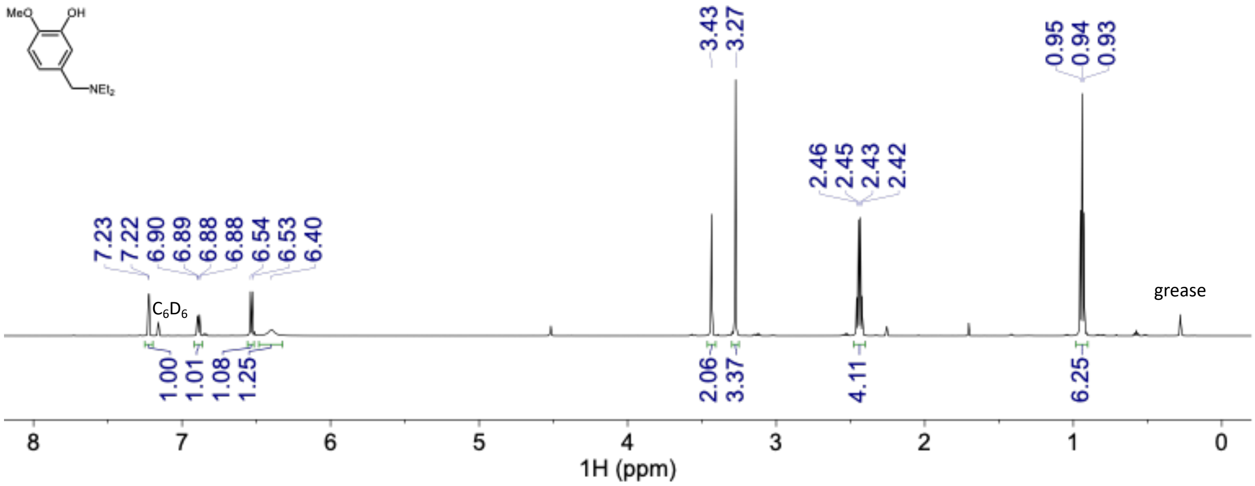

Figure S24. ^{1}H NMR spectrum of 5-((diethylamino)methyl)-2-methoxy phenol (600 MHz, C_6D_6).

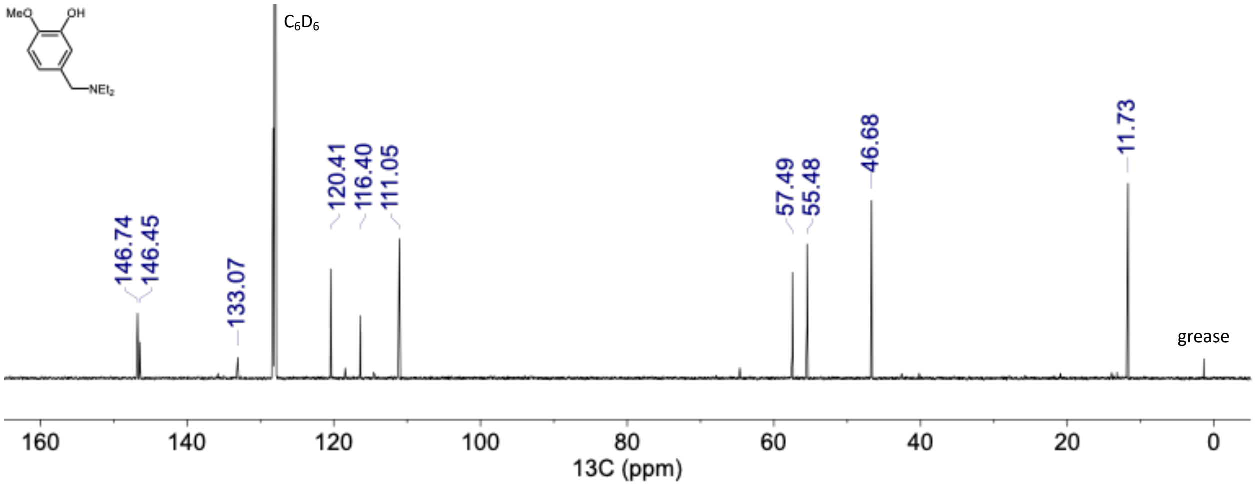

Figure S25. $^{13}C\{^{1}H\}$ NMR spectrum of 5-((diethylamino)methyl)-2-methoxy phenol (151 MHz, C_6D_6).

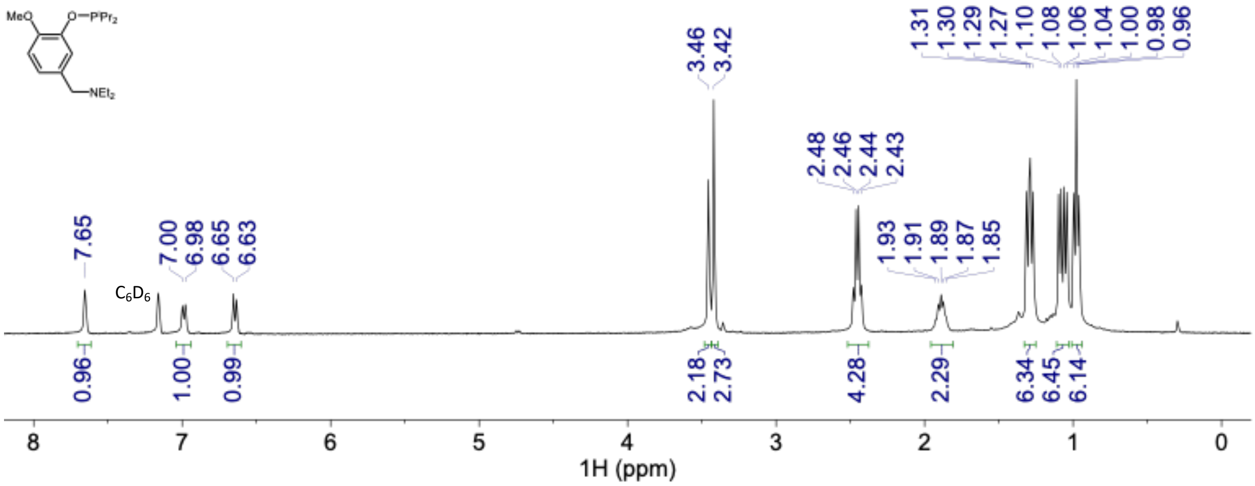

Figure S26. ^{1}H NMR spectrum of ($^{\text{MeO-Et}}$NCOP)H (400 MHz, C_6D_6).

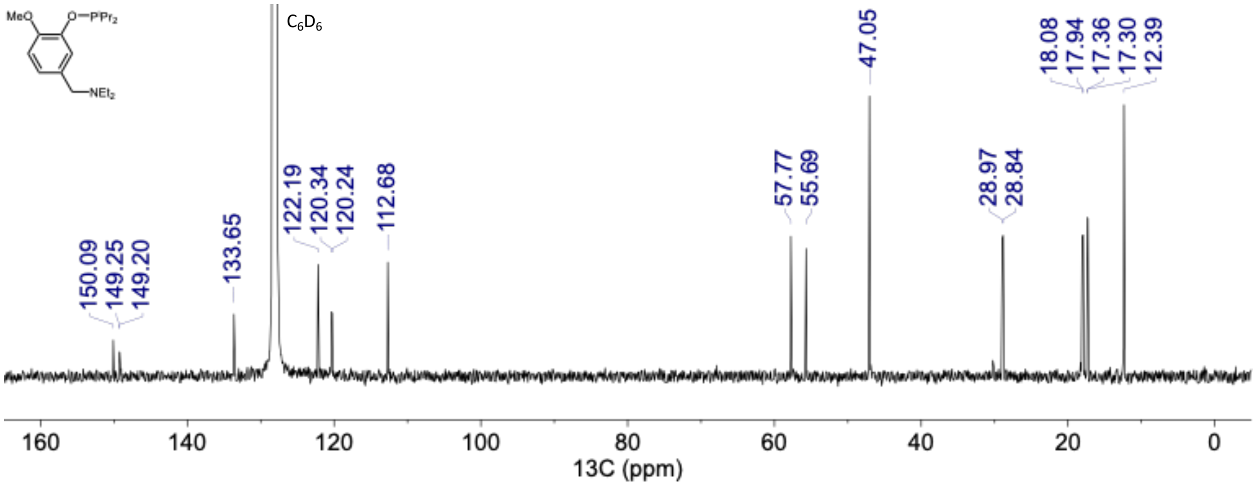

Figure S27. ^{13}C{^{1}H} NMR spectrum of ($^{\text{MeO-Et}}$NCOP)H (151 MHz, C_6D_6).

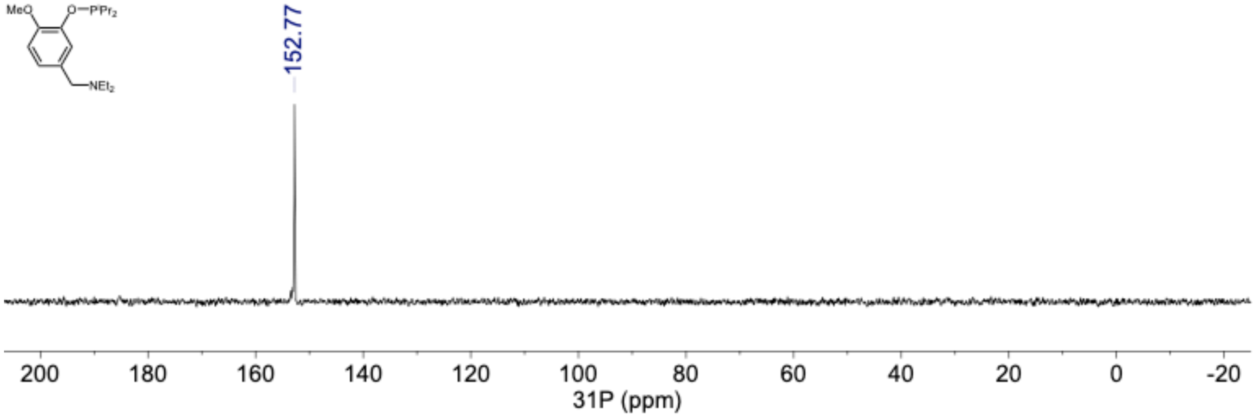

Figure S28. ^{31}P{^{1}H} NMR spectrum of ($^{\text{MeO-Et}}$NCOP)H (162 MHz, C_6D_6).

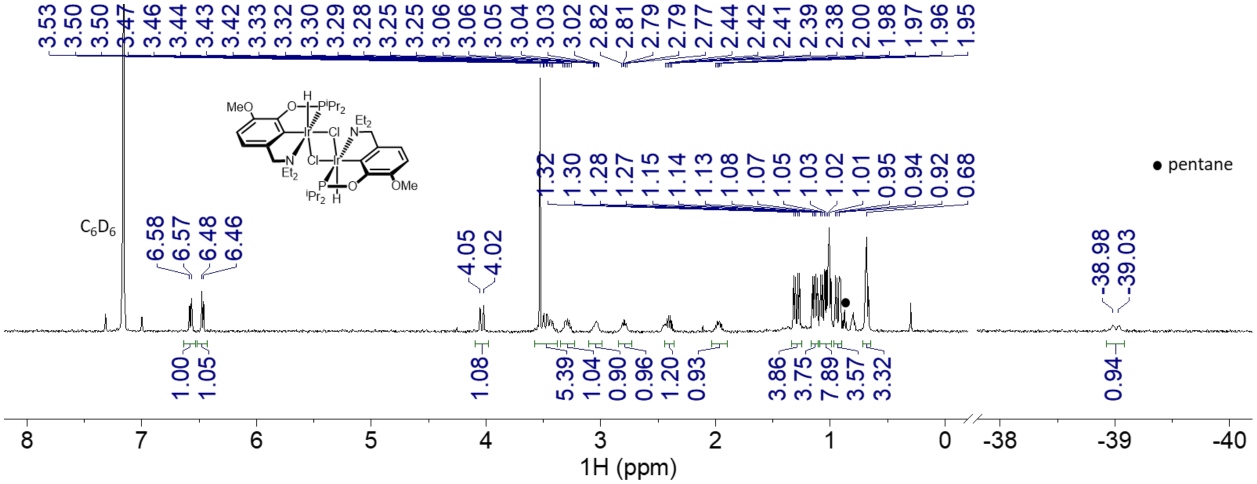

Figure S29. ^{1}H NMR spectrum of **1^{Et}** (500 MHz, C_6D_6).

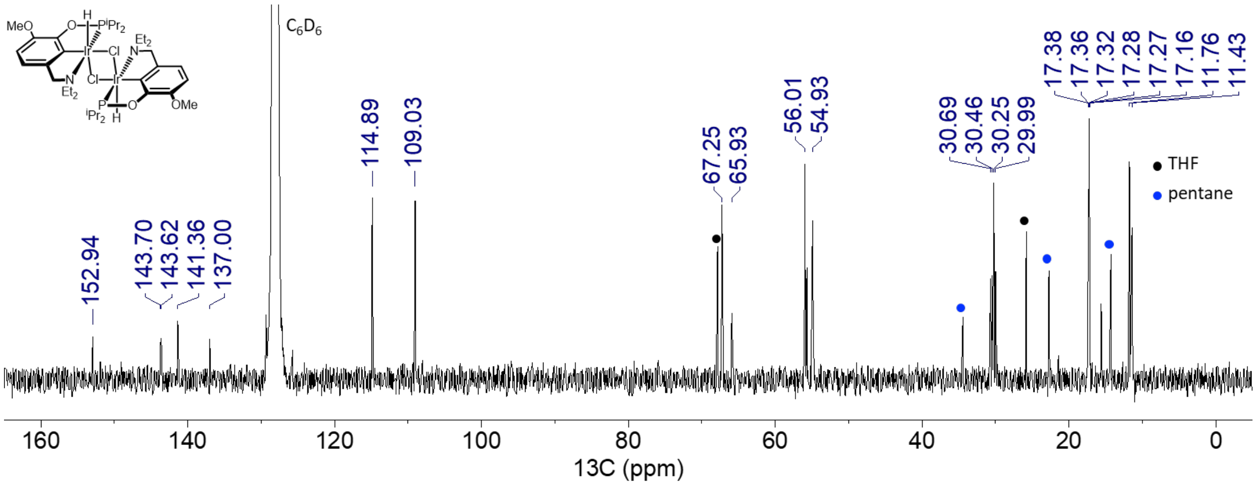

Figure S30. ^{13}C{^{1}H} NMR spectrum of **1^{Et}** (151 MHz, C_6D_6).

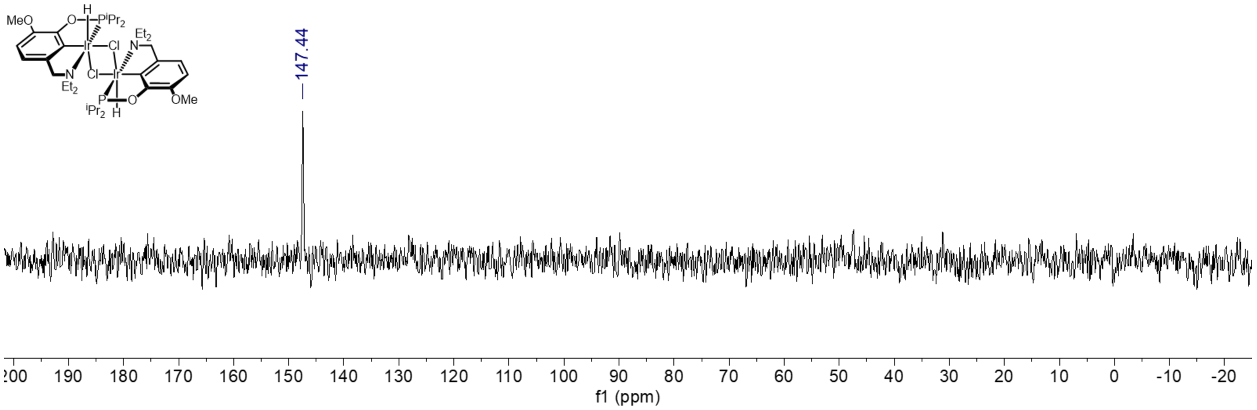

Figure S31. ^{31}P{^{1}H} NMR spectrum of **1^{Et}** (202 MHz, C_6D_6).

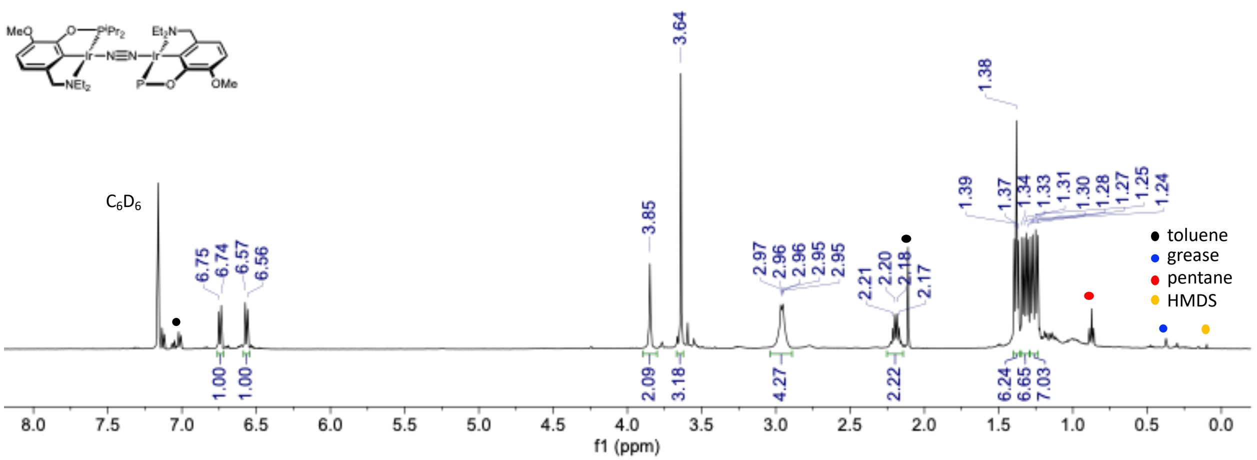

Figure S32. ^{1}H NMR spectrum of **4** (500 MHz, C_6D_6).

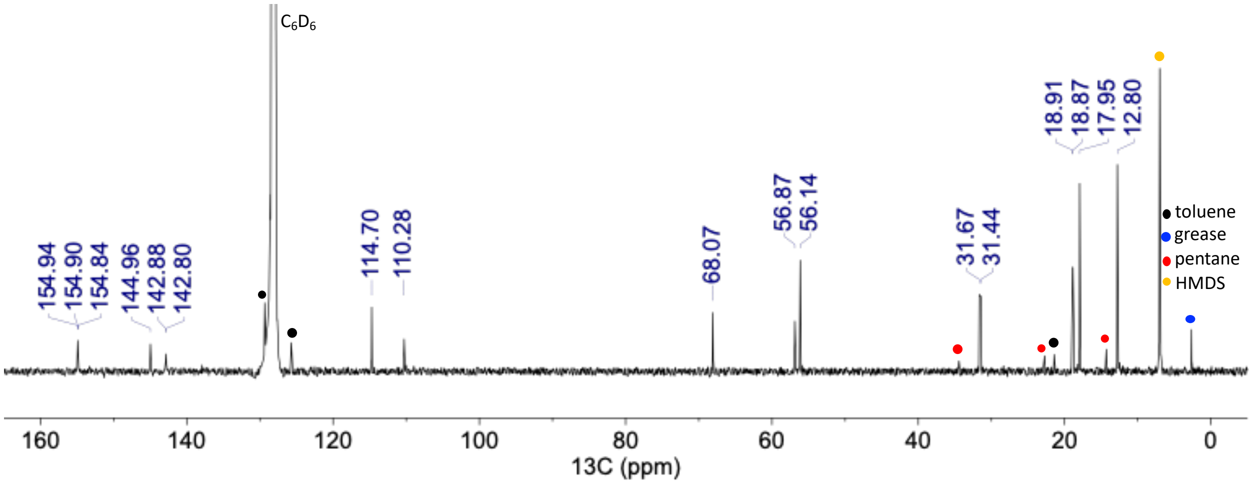

Figure S33. $^{13}C\{^{1}H\}$ NMR spectrum of **4** (151 MHz, C_6D_6).

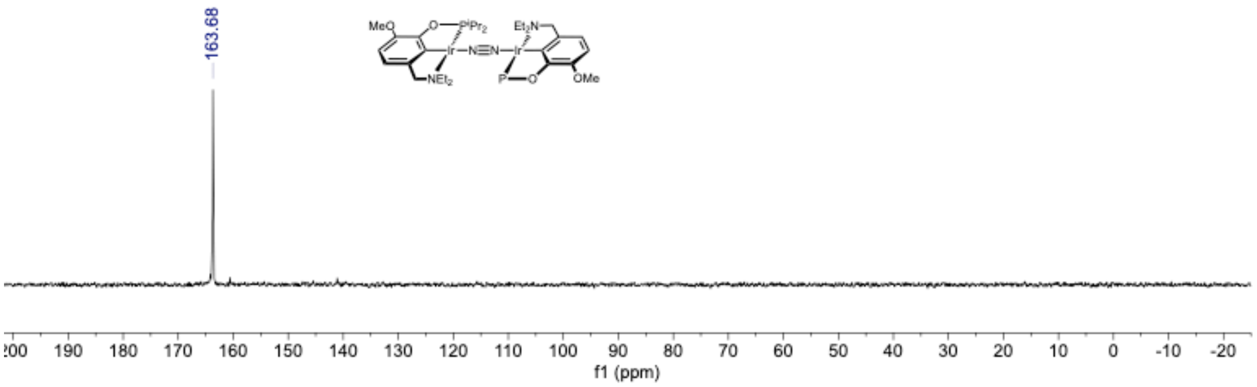

Figure S34. $^{31}P\{^{1}H\}$ NMR spectrum of **4** (202 MHz, C_6D_6).

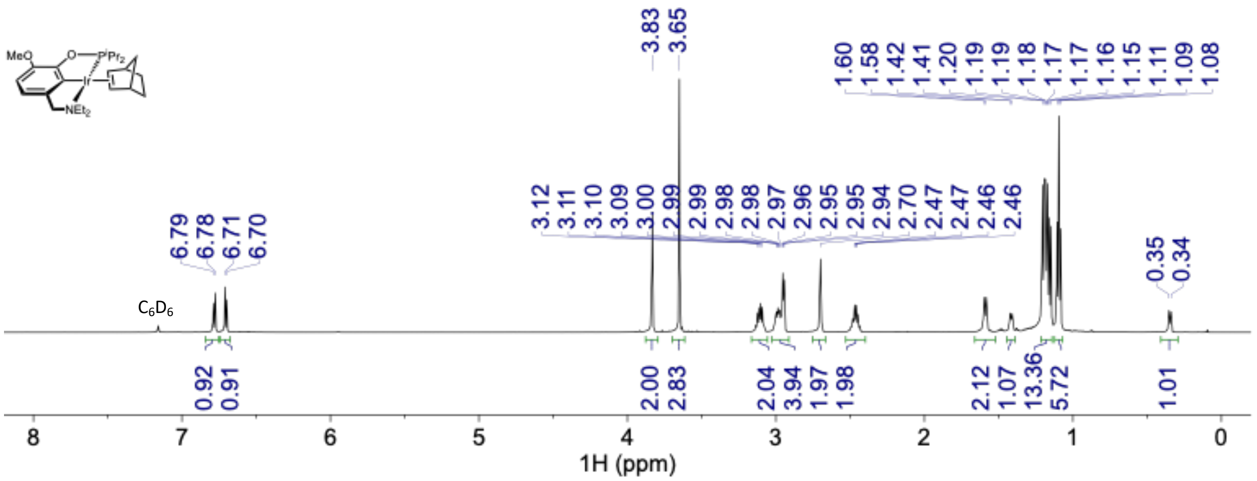

Figure S35. ^{1}H NMR spectrum of **5** (600 MHz, C_6D_6).

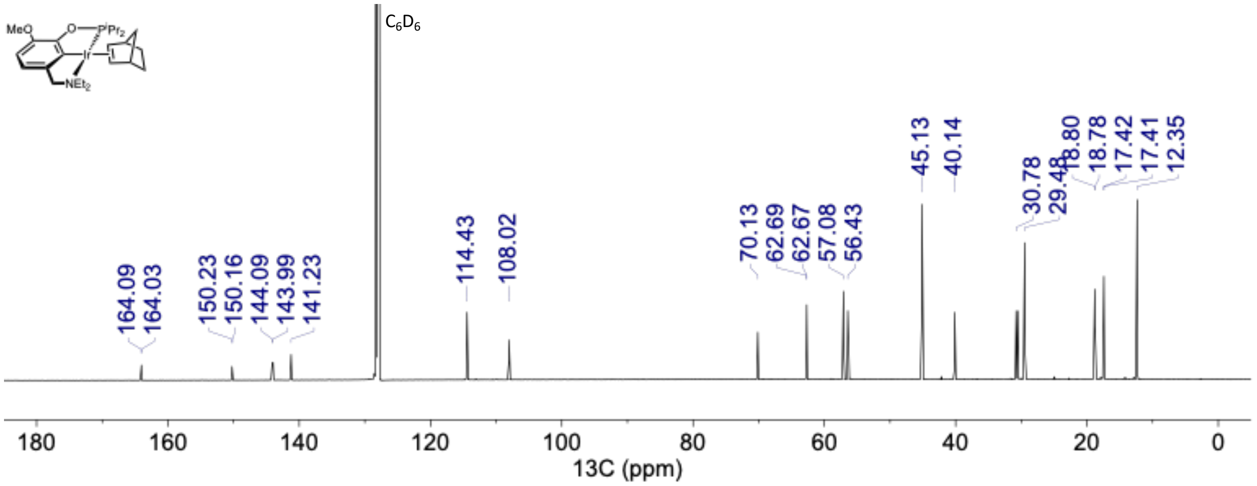

Figure S36. $^{13}C\{^{1}H\}$ NMR spectrum of the mixture of **5** (151 MHz, C_6D_6).

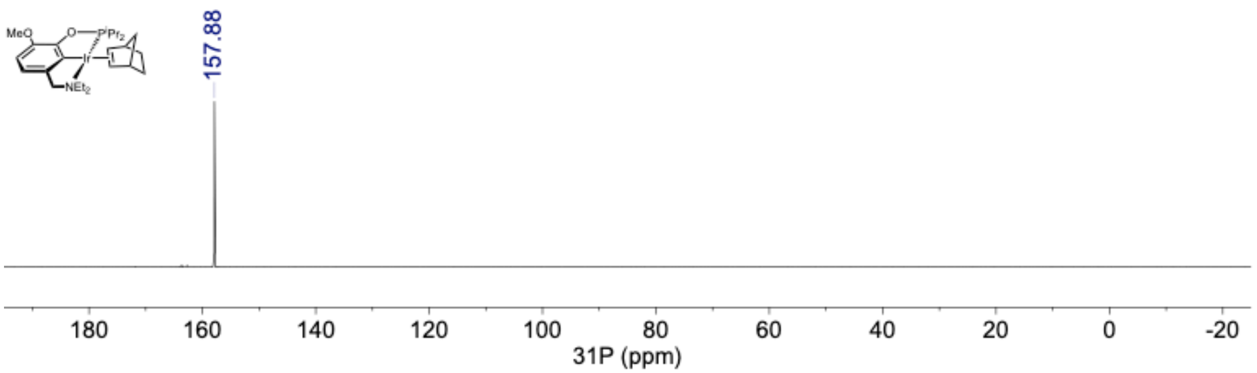

Figure S37. $^{31}P\{^{1}H\}$ NMR spectrum of the mixture of **5** (243 MHz, C_6D_6).

III. 2-Dimensional NMR Spectra

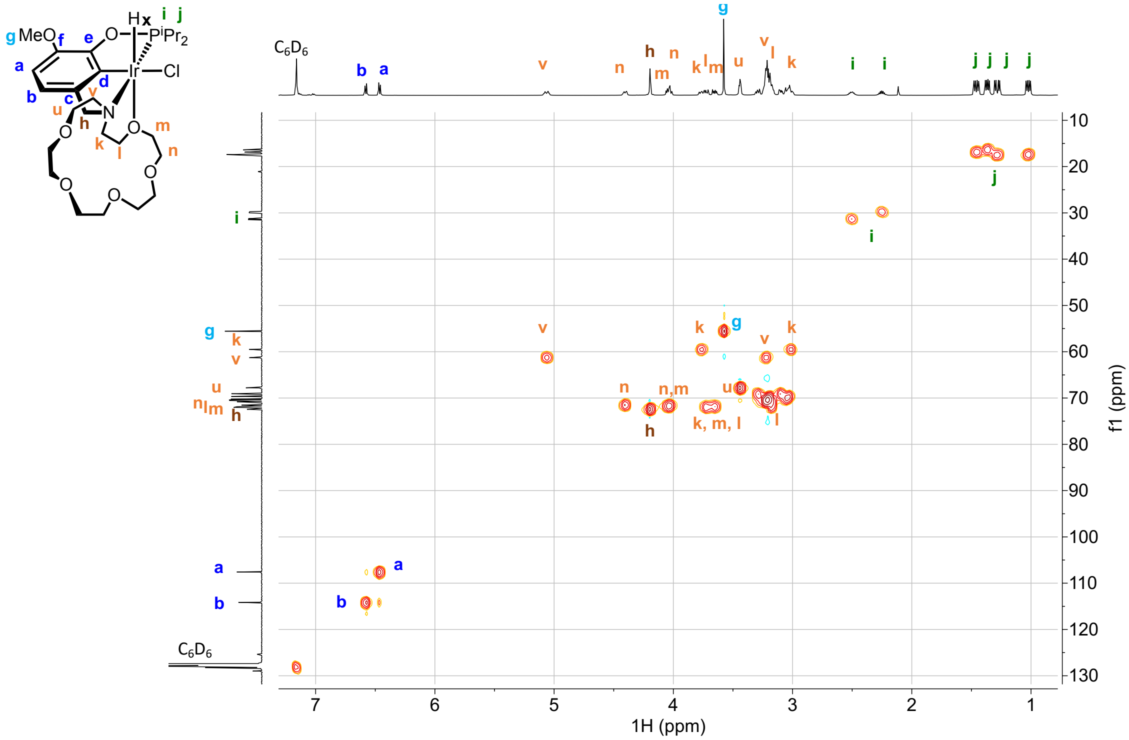

Figure S38. ^{1}H-^{13}C HSQC NMR spectrum of **1^{18c6}** (600 MHz, C_6D_6).

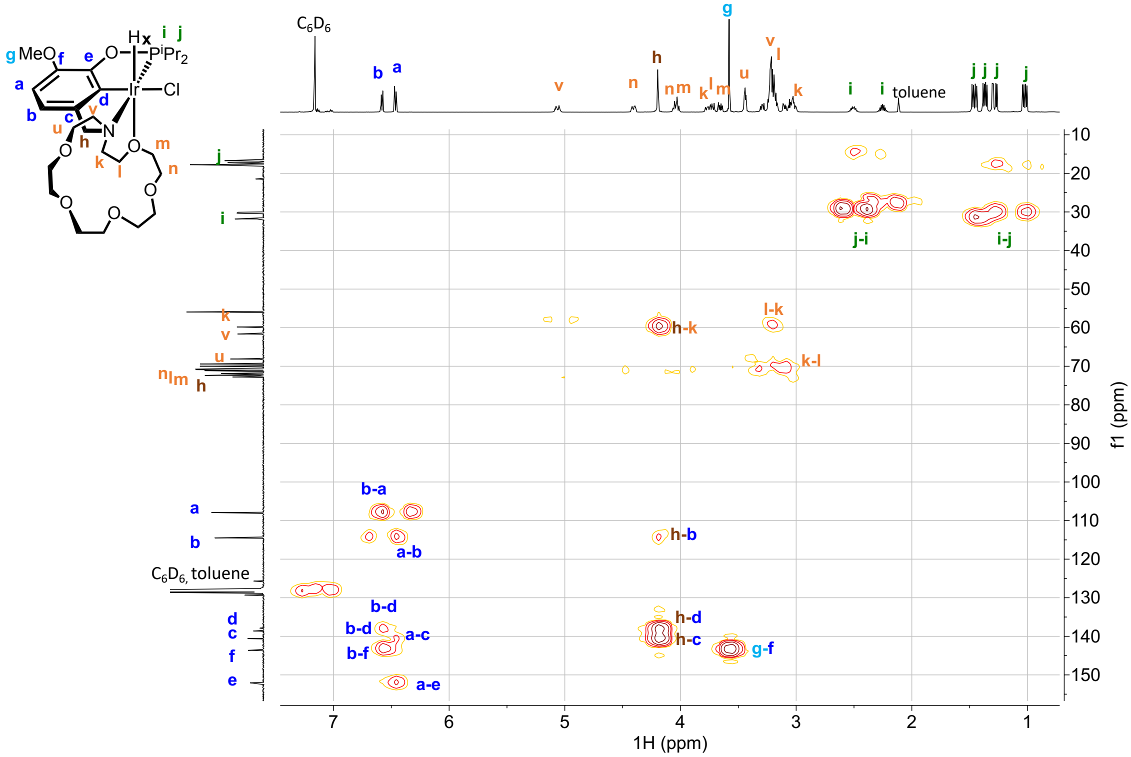

Figure S39. ^{1}H-^{13}C HMBC NMR spectrum of **1^{18c6}** (600 MHz, C_6D_6).

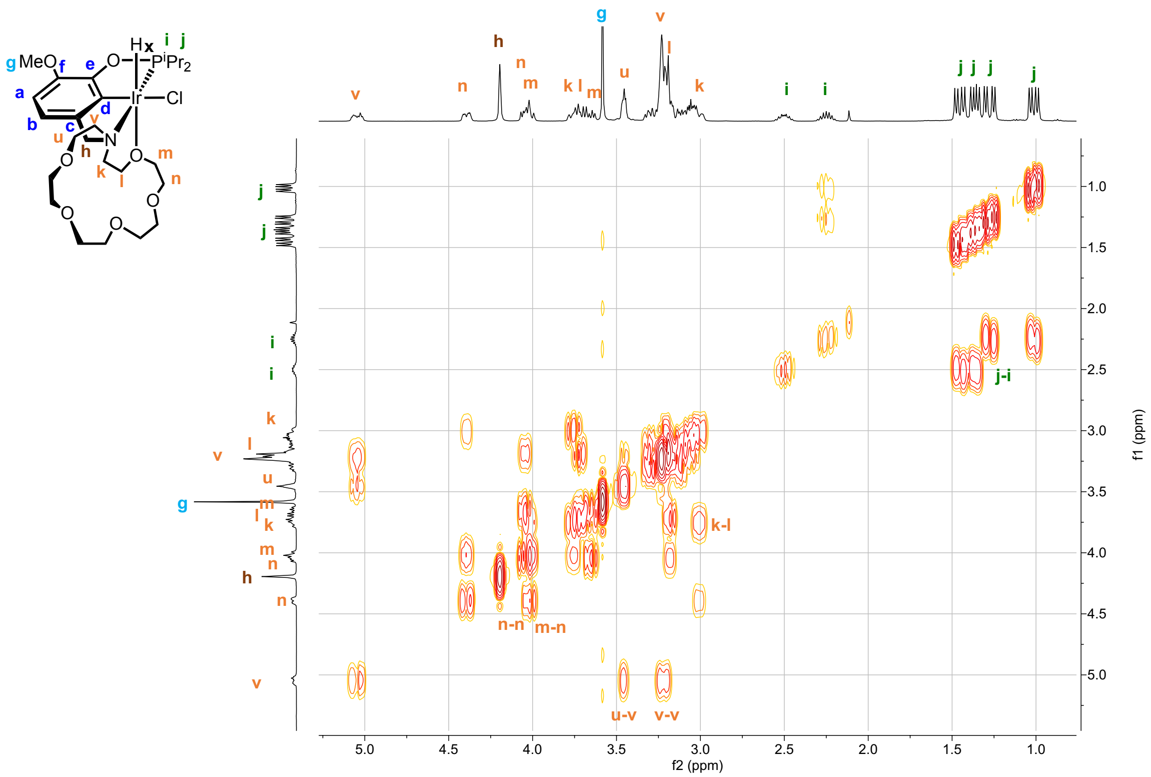

Figure S40. ^{1}H-^{1}H COSY NMR spectrum of $\mathbf{1^{18c6}}$ (600 MHz, C_6D_6).

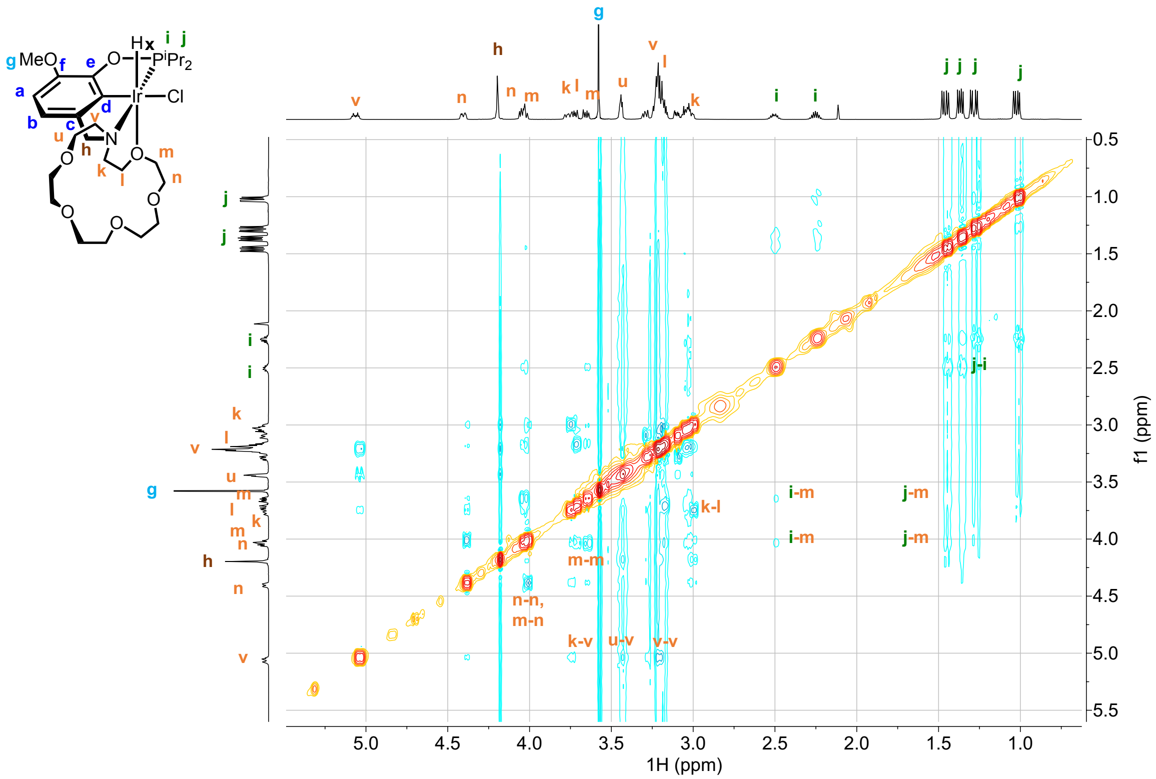

Figure S41. ^{1}H-^{1}H NOESY NMR spectrum of $\mathbf{1^{18c6}}$ (600 MHz, C_6D_6).

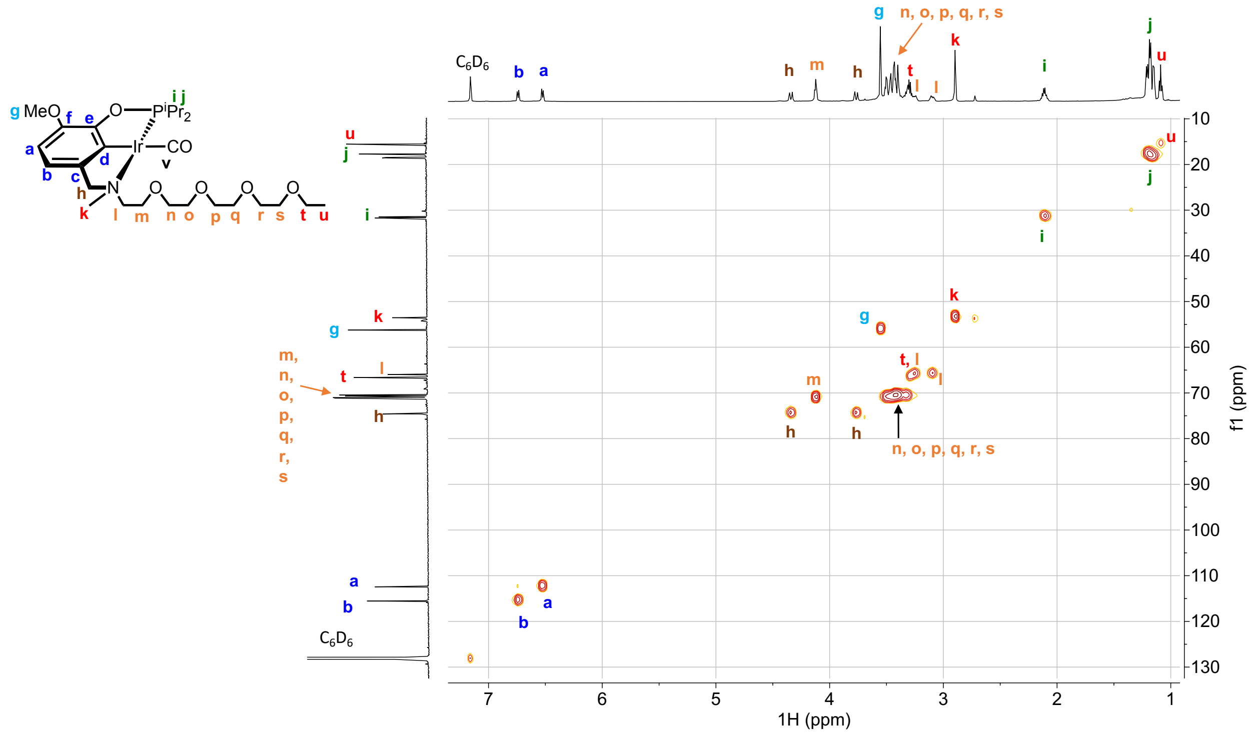

Figure S42. ^{1}H-^{13}C HSQC NMR spectrum of $\mathbf{2^{18c6}}$ (600 MHz, C_6D_6).

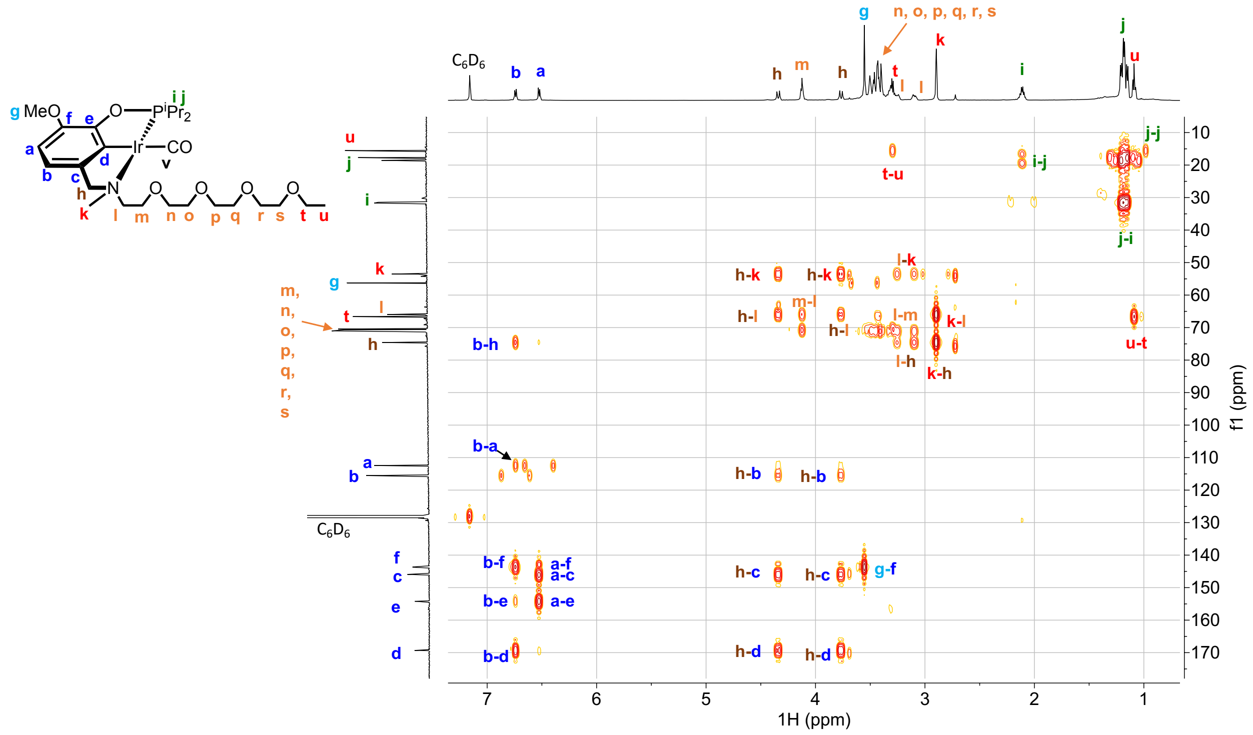

Figure S43. ^{1}H-^{13}C HMBC NMR spectrum of $\mathbf{2^{18c6}}$ (600 MHz, C_6D_6).

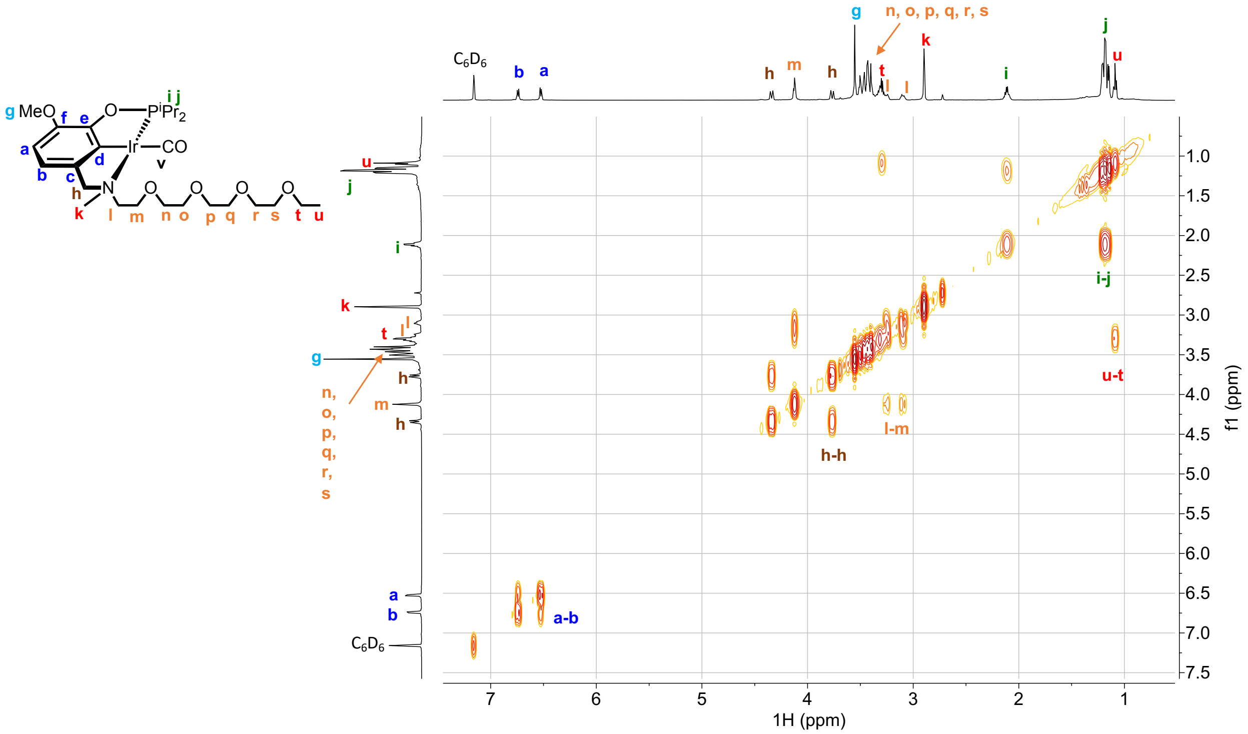

Figure S44. ^{1}H-^{1}H COSY NMR spectrum of $\mathbf{2}^{\mathbf{18c6}}$ (600 MHz, C_6D_6).

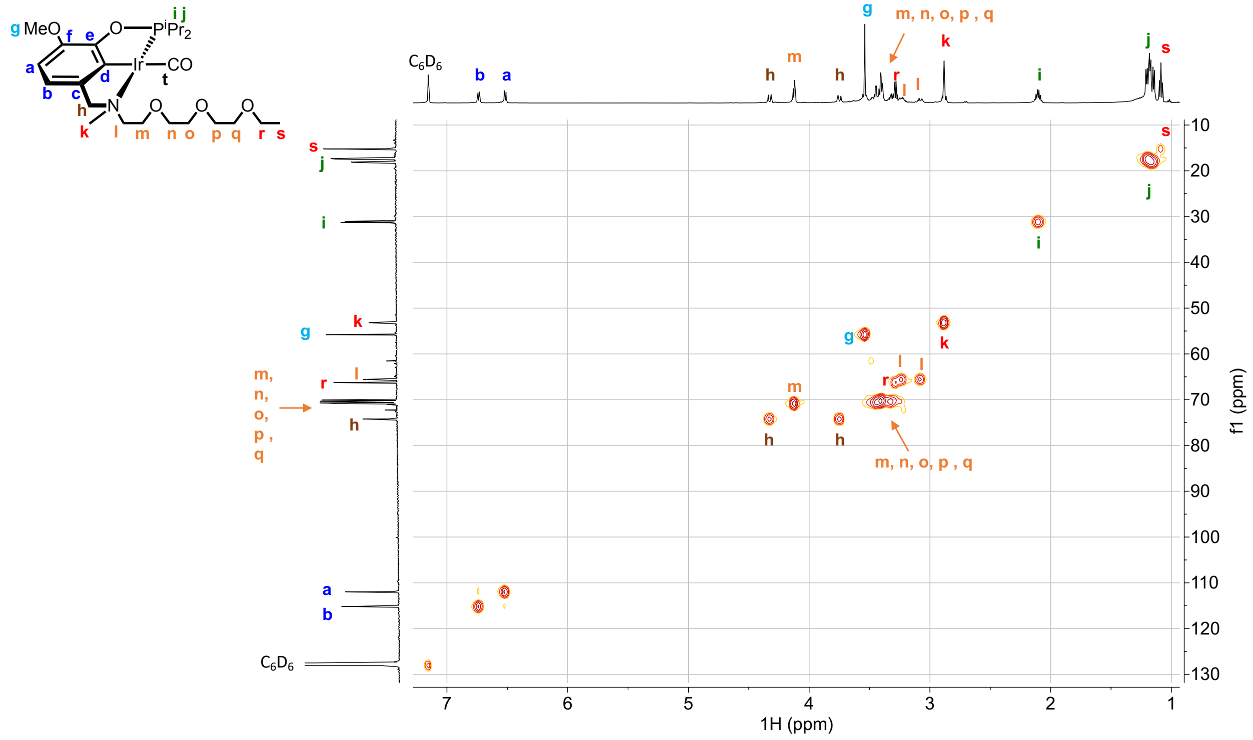

Figure S45. ^{1}H-^{13}C HSQC NMR spectrum of $\mathbf{2}^{15c5}$ (600 MHz, C_6D_6).

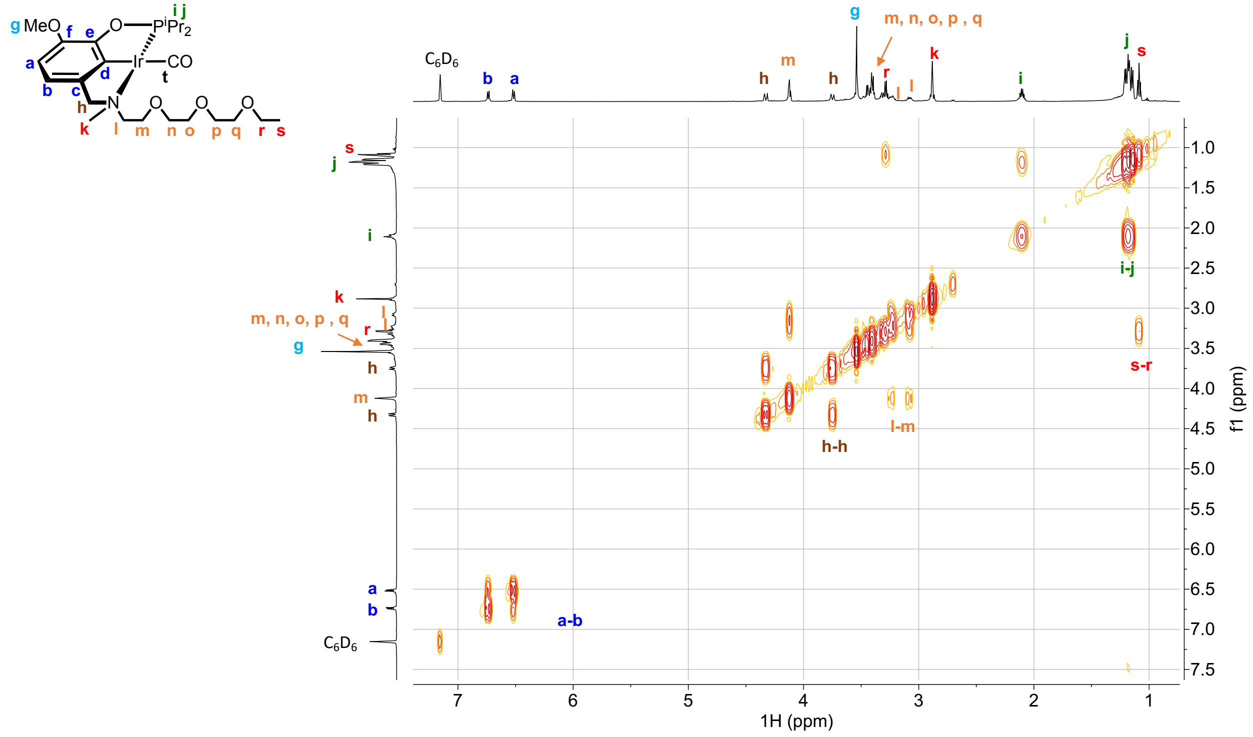

Figure S46. ^{1}H-^{1}H COSY NMR spectrum of $\mathbf{2}^{15c5}$ (600 MHz, C_6D_6).

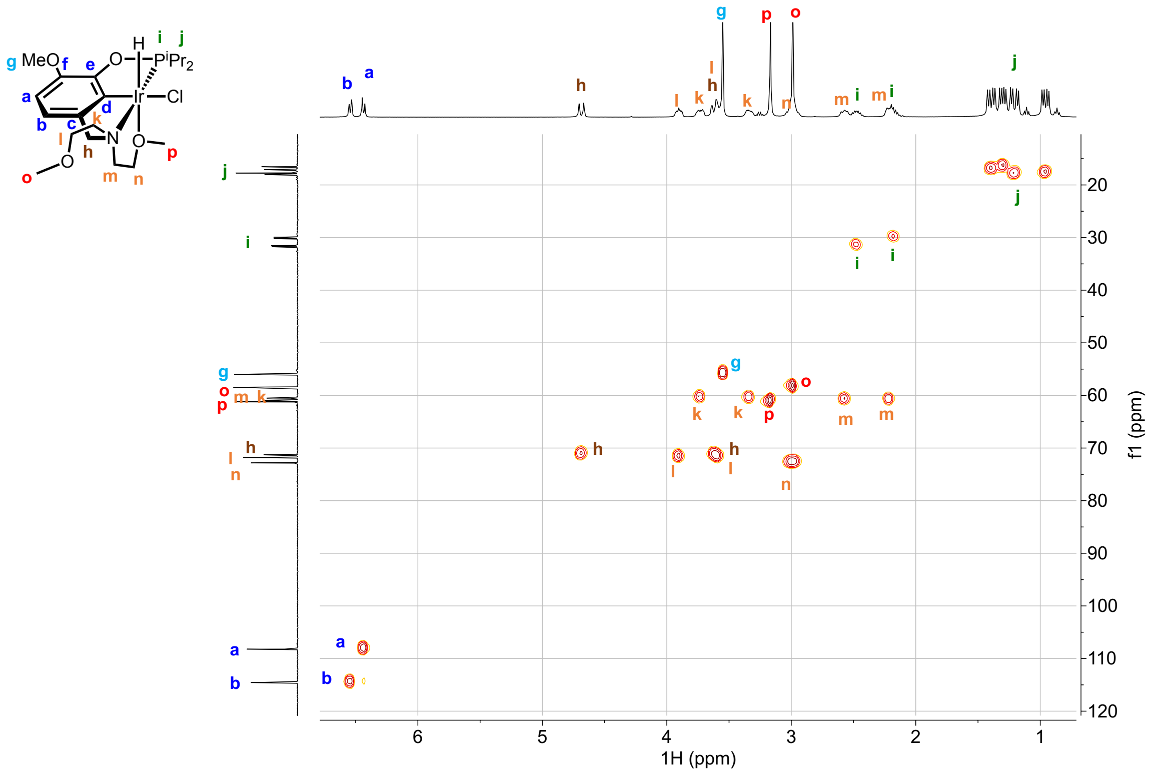

Figure S47. ^{1}H-^{13}C HSQC NMR spectrum of $\mathbf{1^{BME}}$ (600 MHz, C_6D_6).

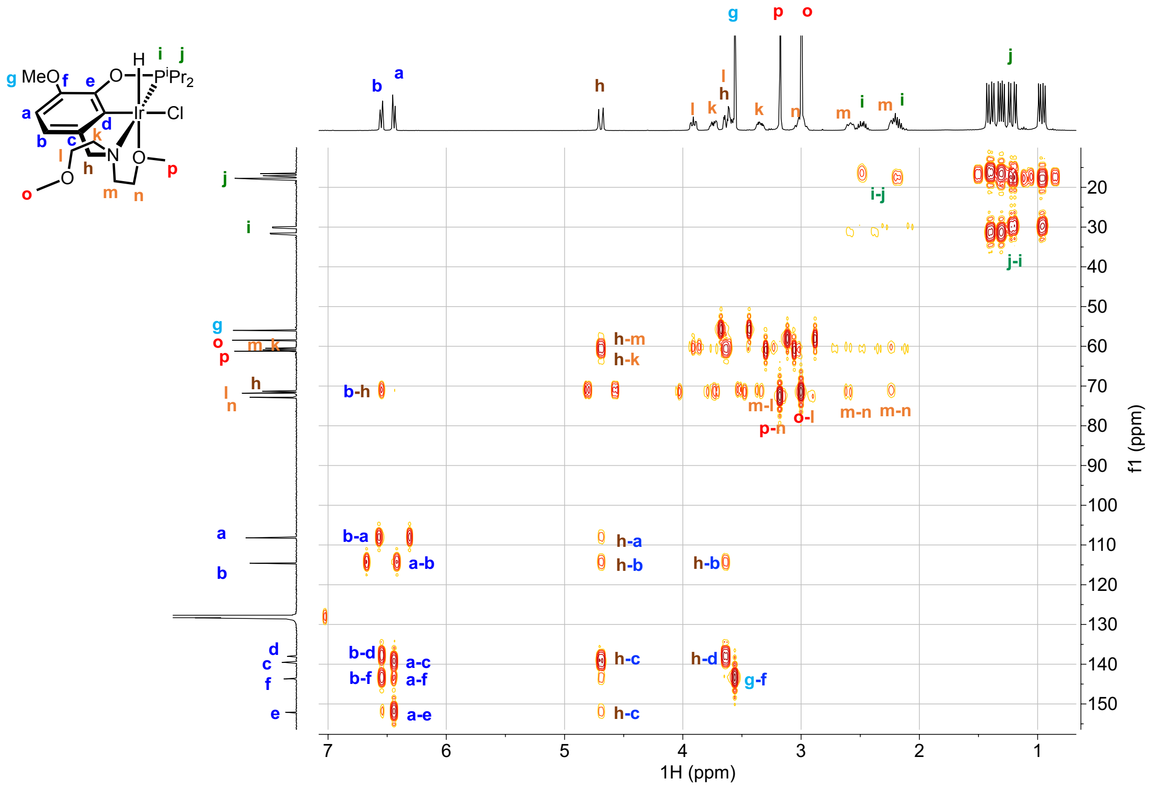

Figure S48. ^{1}H-^{13}C HMBC NMR spectrum of $\mathbf{1^{BME}}$ (600 MHz, C_6D_6).

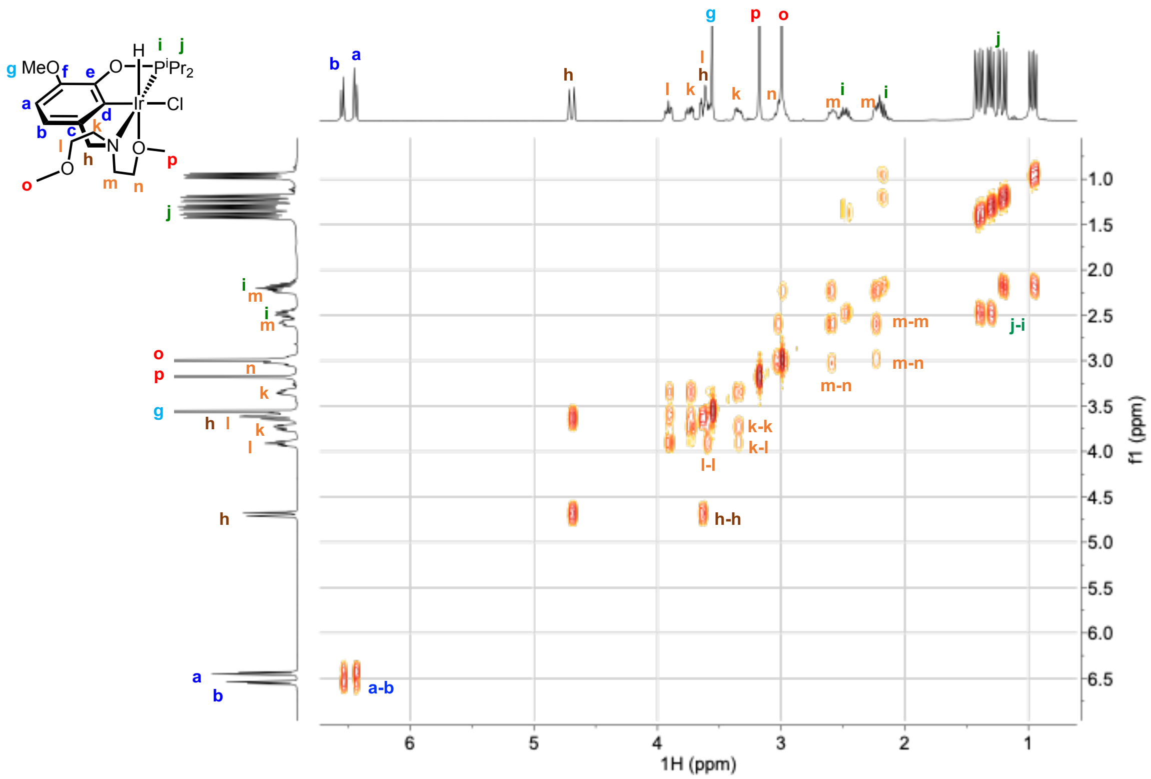

Figure S49. ^{1}H-^{1}H COSY NMR spectrum of $\mathbf{1}^{\mathbf{BME}}$ (600 MHz, C_6D_6).

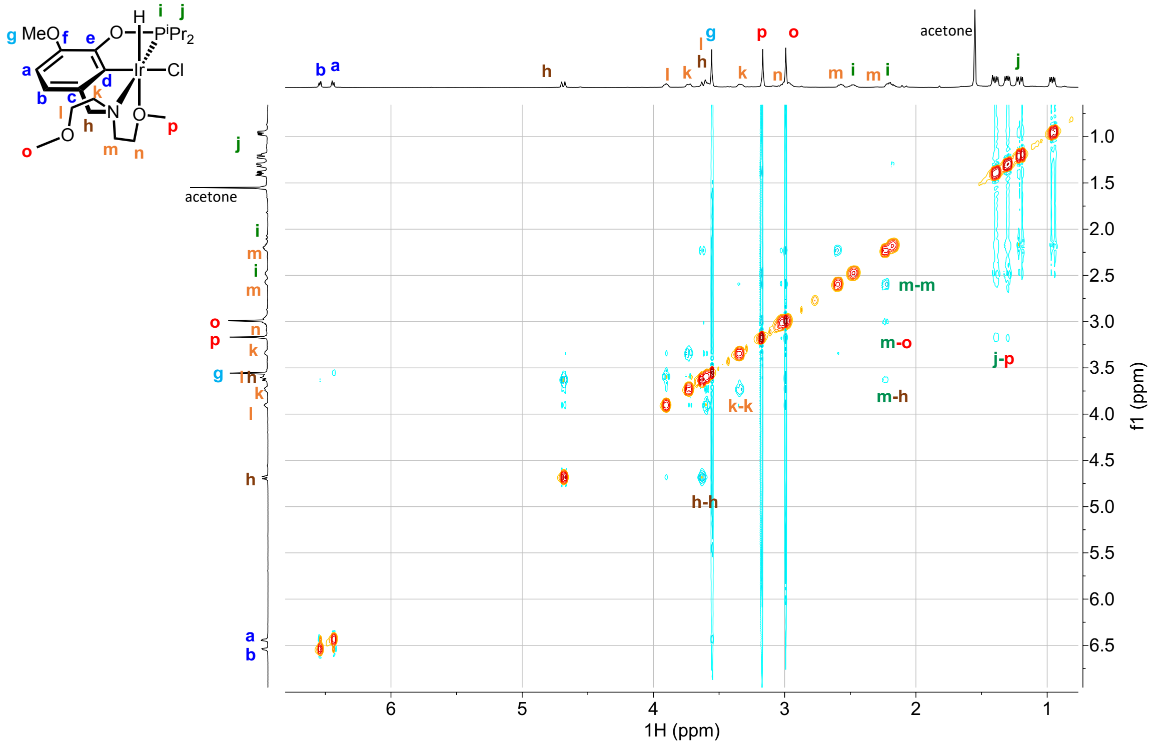

Figure S50. ^{1}H-^{1}H NOESY NMR spectrum of $\mathbf{1}^{\mathbf{BME}}$ (600 MHz, C_6D_6).

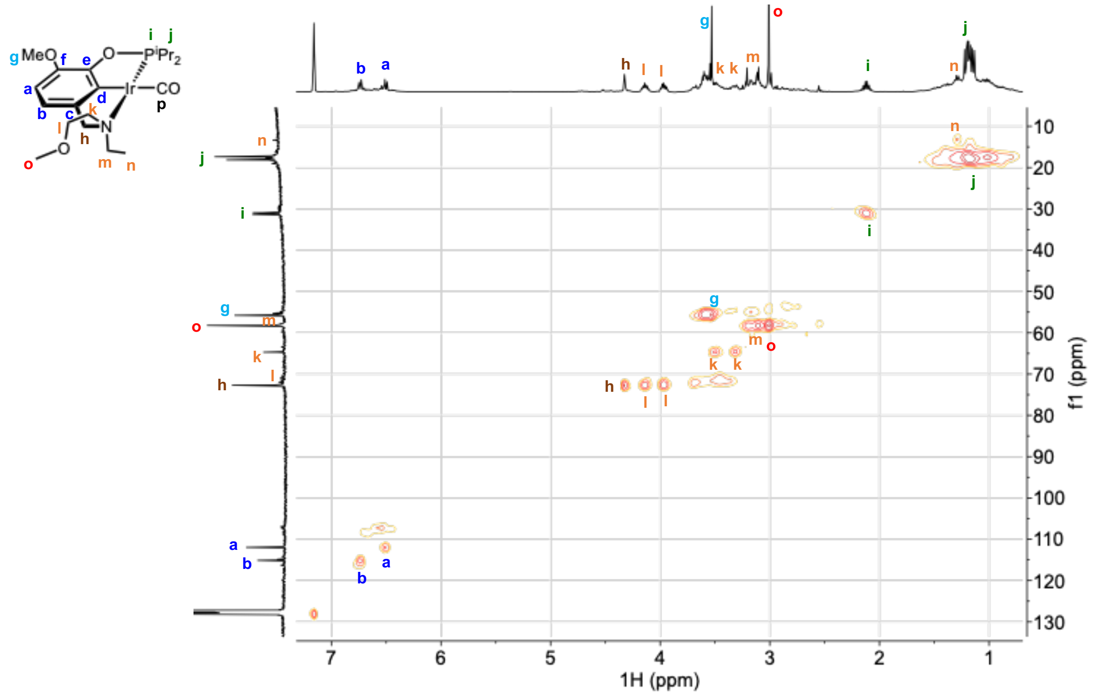

Figure S51. ^{1}H-^{1}H HSQC NMR spectrum of the mixture of **3^{BME}** and **2^{BME}** (400 MHz, C_6D_6). Only the major product **3^{BME}** was assigned.

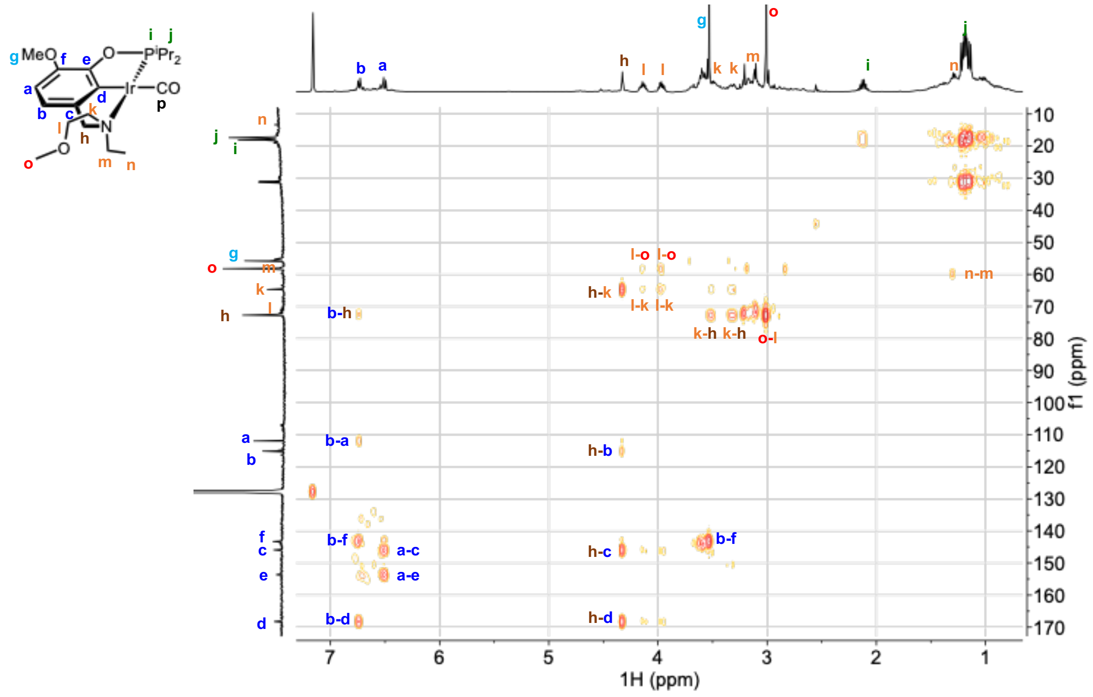

Figure S52. ^{1}H-^{1}H HMBC NMR spectrum of the mixture of **3^{BME}** and **2^{BME}** (400 MHz, C_6D_6). Only the major product **3^{BME}** was assigned.

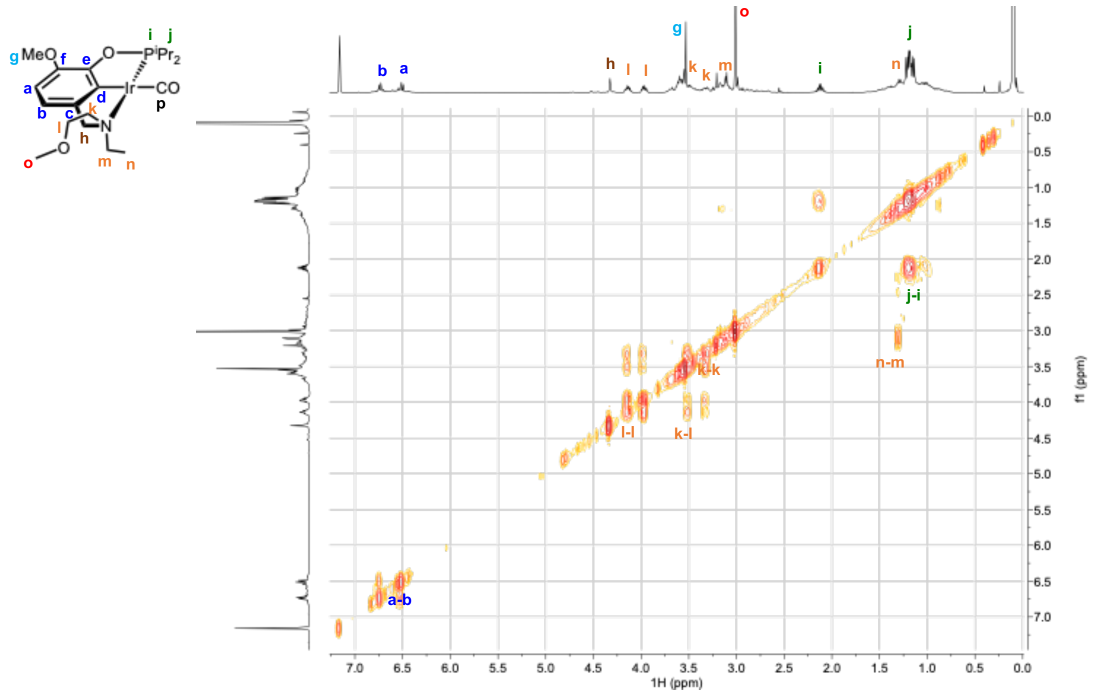

Figure S53. ^1H-^1H COSY NMR spectrum of the mixture of $\mathbf{3^{BME}}$ and $\mathbf{2^{BME}}$ (400 MHz, C_6D_6). Only the major product $\mathbf{3^{BME}}$ was assigned.

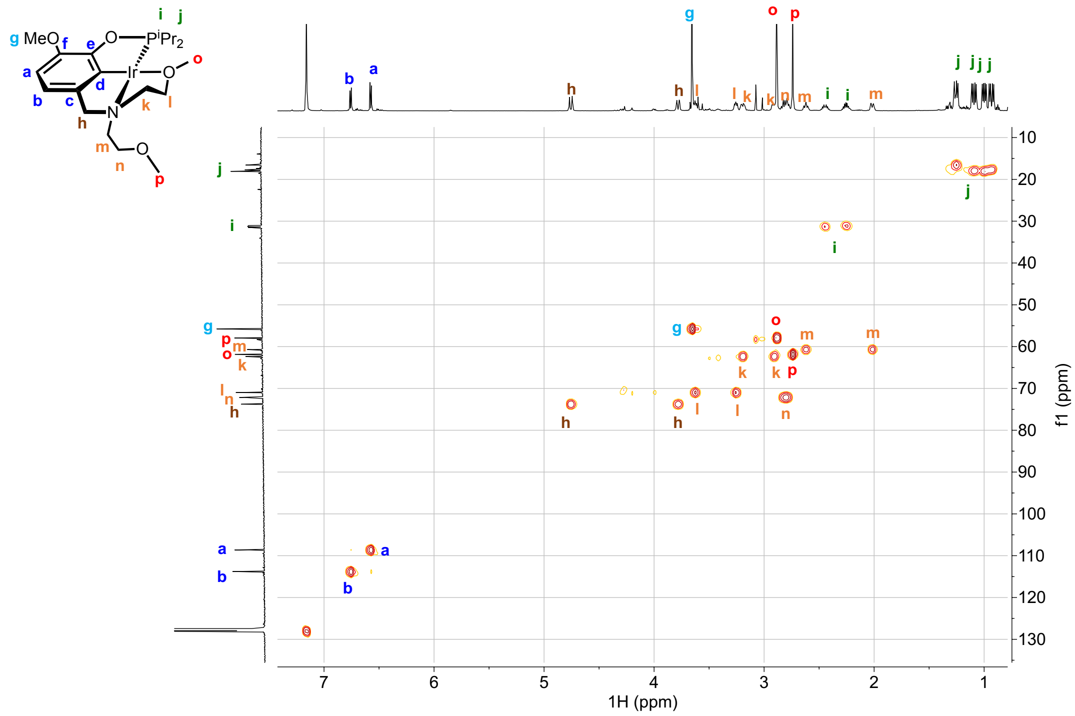

Figure S54. ^{1}H-^{13}C HSQC NMR spectrum of (κ^{4}-$^{MeO\text{-}BME}$NCOP)Ir (600 MHz, C_6D_6).

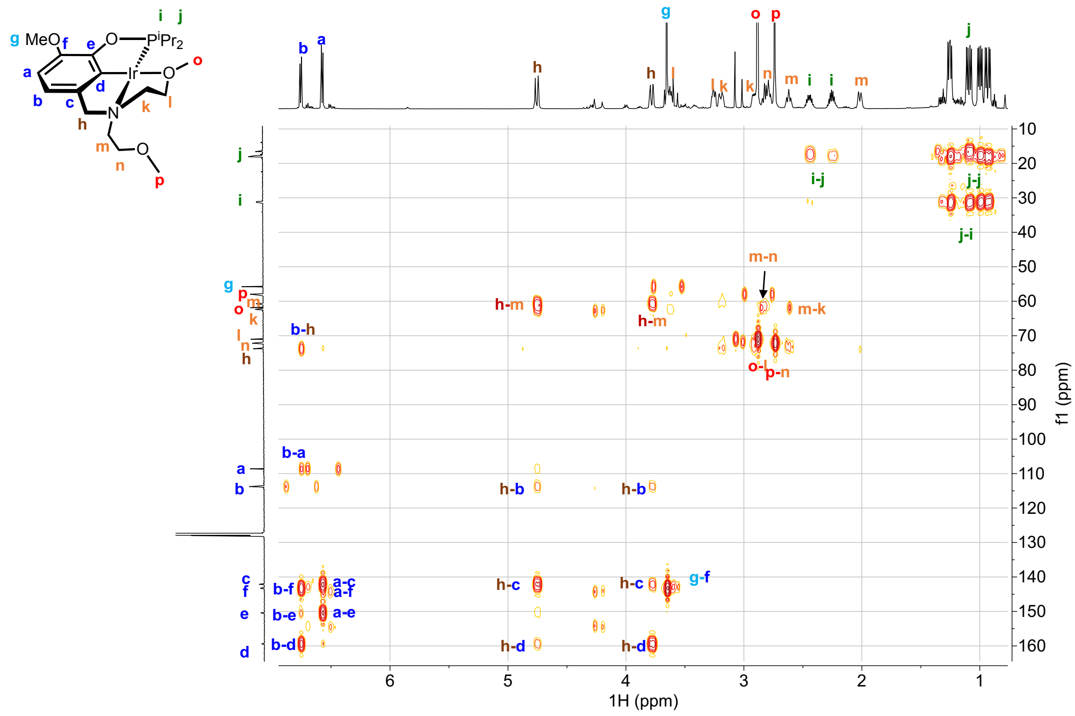

Figure S55. ^{1}H-^{13}C HMBC NMR spectrum of (κ^{4}-$^{MeO\text{-}BME}$NCOP)Ir (600 MHz, C_6D_6).

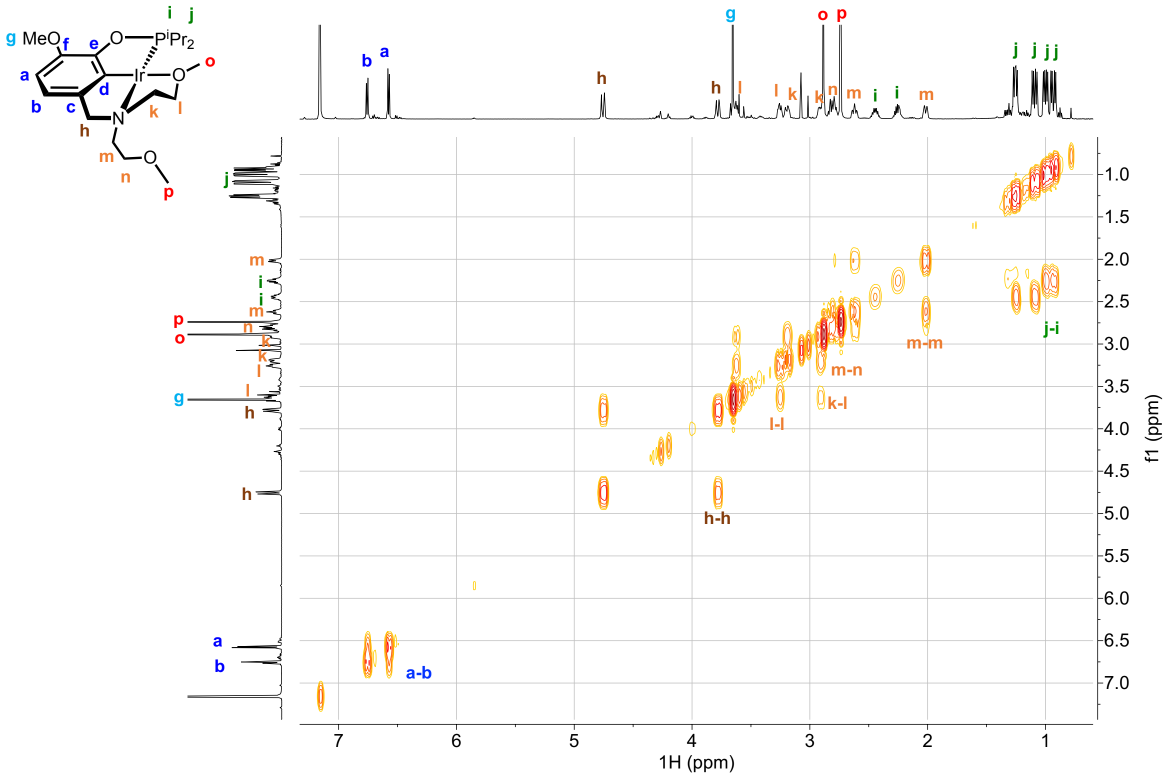

Figure S56. ^{1}H-^{1}H COSY NMR spectrum of (κ^{4}-$^{MeO-BME}$NCOP)Ir (600 MHz, C_6D_6).

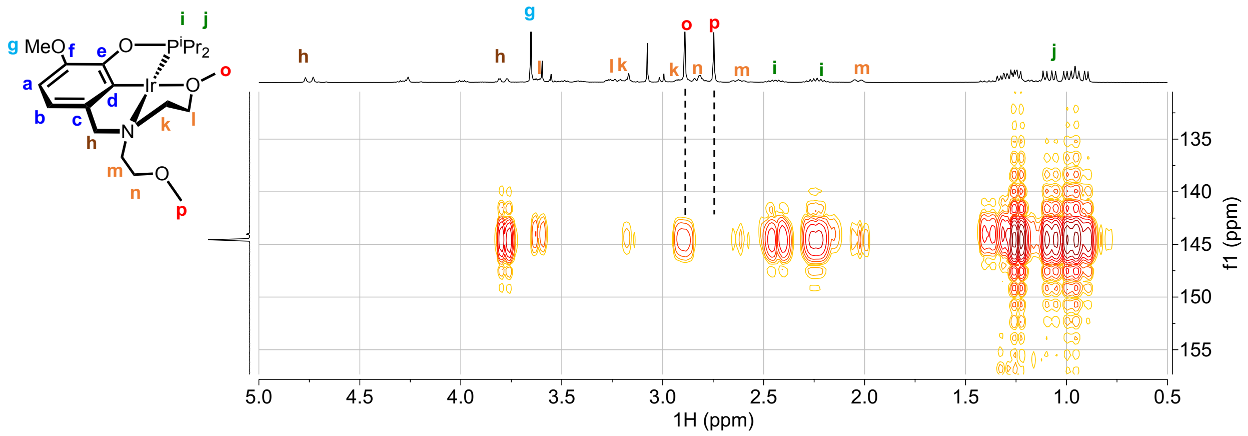

Figure S57. ^{1}H-^{31}P HMBC NMR spectrum of (κ^{4}-$^{MeO-BME}$NCOP)Ir (600 MHz, C_6D_6).

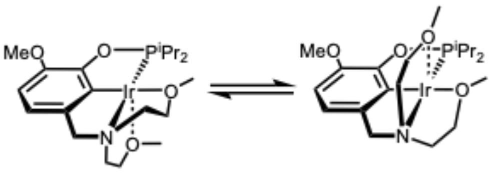

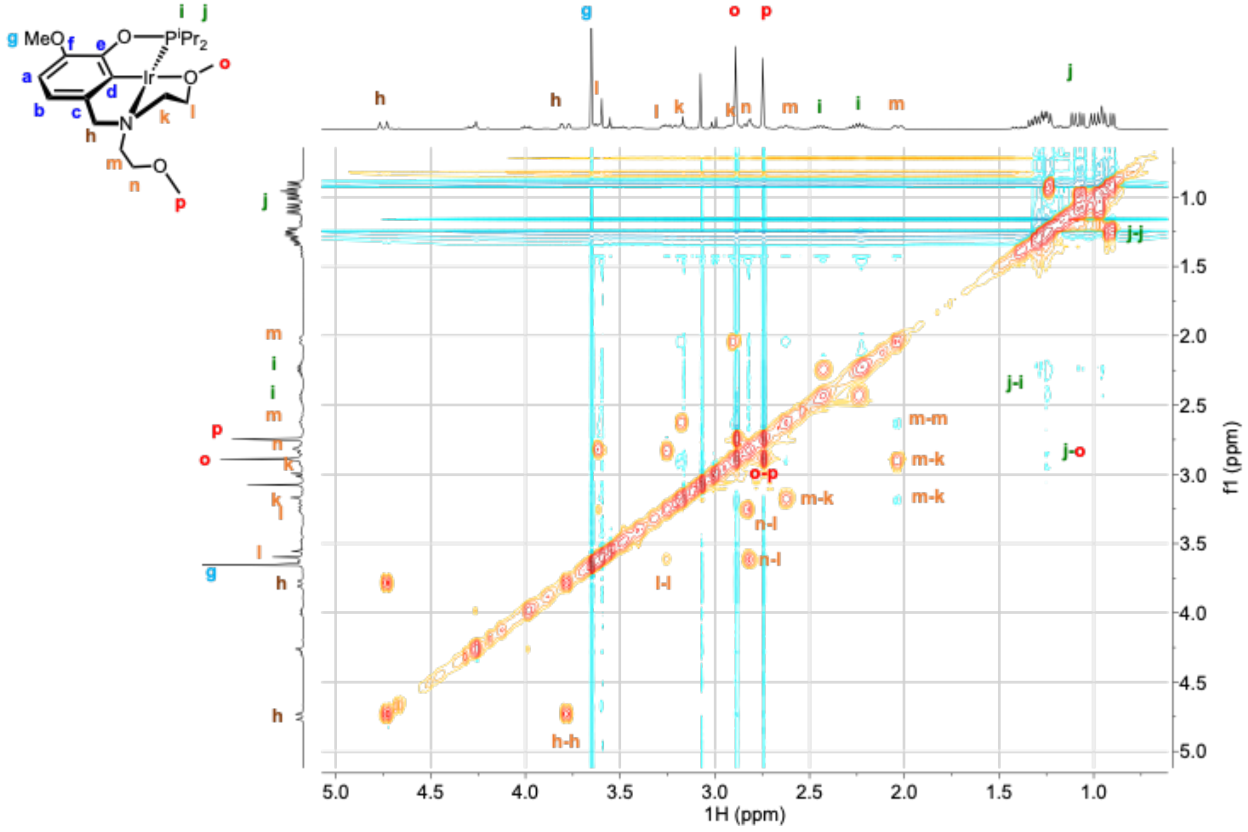

Figure S58. ^1H-^1H NOESY NMR spectrum of $(\kappa^{4}\text{-}^{MeO\text{-}BME}NCOP)Ir$ (600 MHz, C_6D_6).

IV. General Procedure for Yield Determination for Intermolecular Decarbonylation

In a Teflon-sealed NMR tube, ca 20 mg iridium complex was dissolved in 0.5 mL C_6D_6. The desired ether was added and the reaction mixture was heated at 80 °C for 24 h. After the reaction mixture was cooled to room temperature, triphenyl phosphate solution in C_6D_6 was added as an internal integration standard. The yield of the **6** was obtained from integration of inverse-gated decoupled ^{31}P NMR (single pulse). The yield of the di- and tetra(ethylene glycol) ethyl methyl ether from crown ether activation was obtained from integration of inverse-gated decoupled ^{13}C NMR using anisole as an internal standard (delay time of 5 seconds). Yield of isobutane from MeO^tBu activation was obtained from relative integration of ^1H NMR signal (single pulse) compared to **6** in the reaction mixture. Identity of dimethyl ether and methane is confirmed by ^1H NMR and gas chromatography.

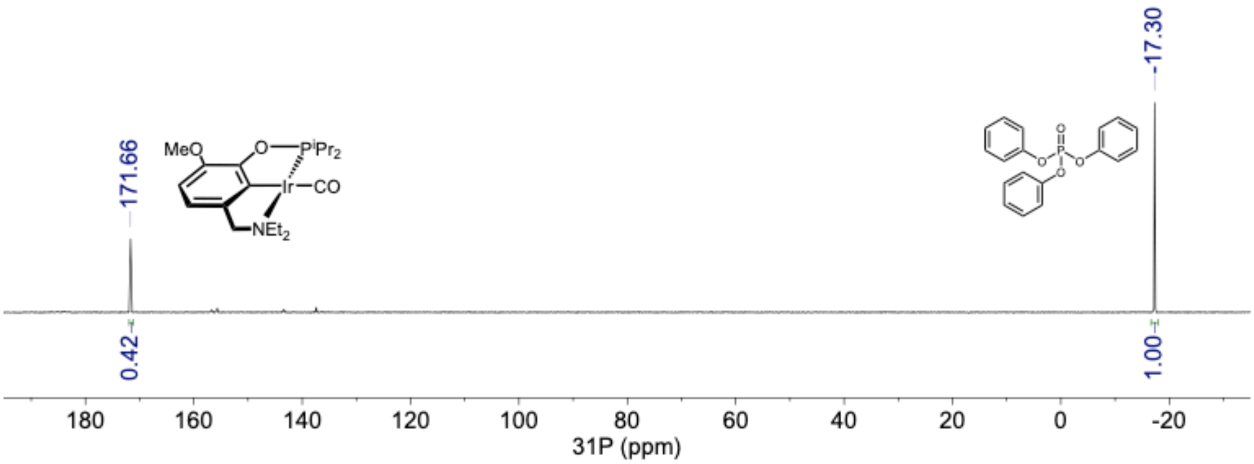

Figure S59. Example inverse-gated decoupled ^{31}P NMR spectrum following intermolecular ether decarbonylation. Relative integration against triphenyl phosphate internal standard is used to calculate the mmol of products.

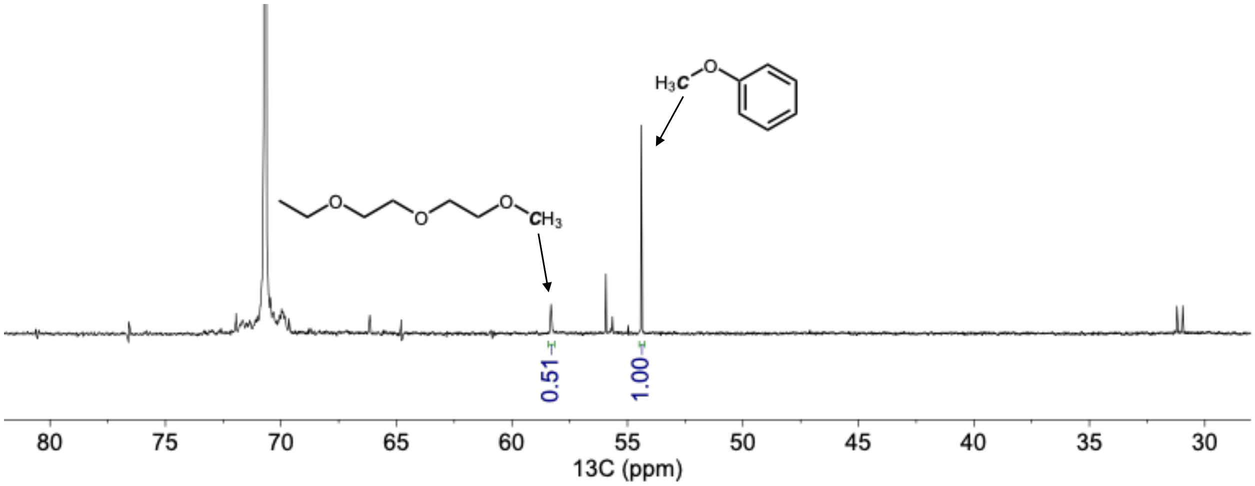

Figure S60. Example inverse-gated decoupled ^{13}C NMR spectrum following intermolecular decarbonylation of 12-crown-4 ether. Relative integration of the $\mathit{C}H_3O$ peak against that of anisole internal standard is used to calculate the mmol of products.

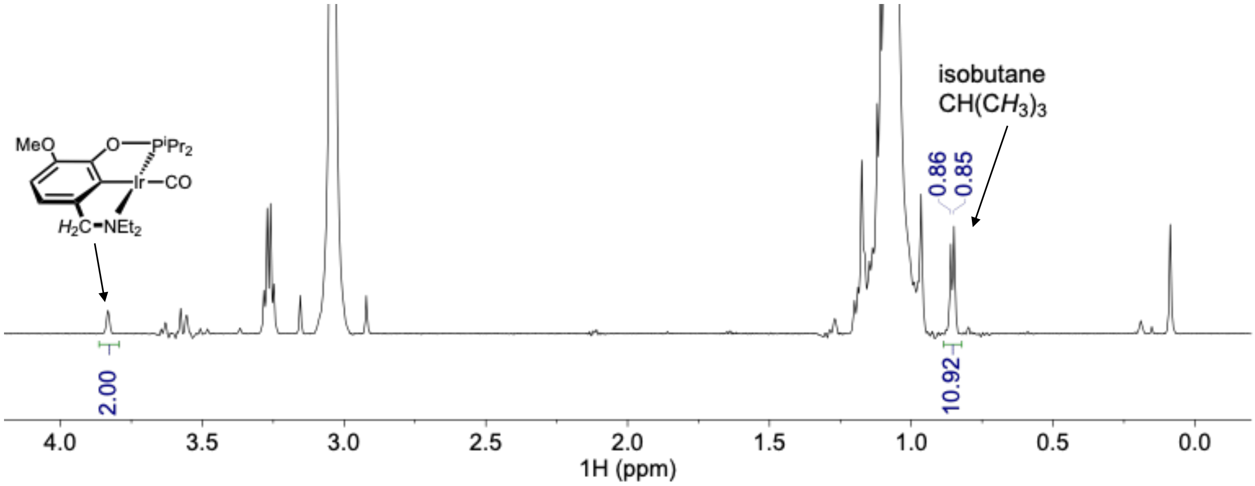

Figure S61. Example ^{1}H NMR spectrum following intermolecular decarbonylation of MeO^tBu. Relative integration of isobutane CH_3 peak against **6** is used to calculate the mmol of product.

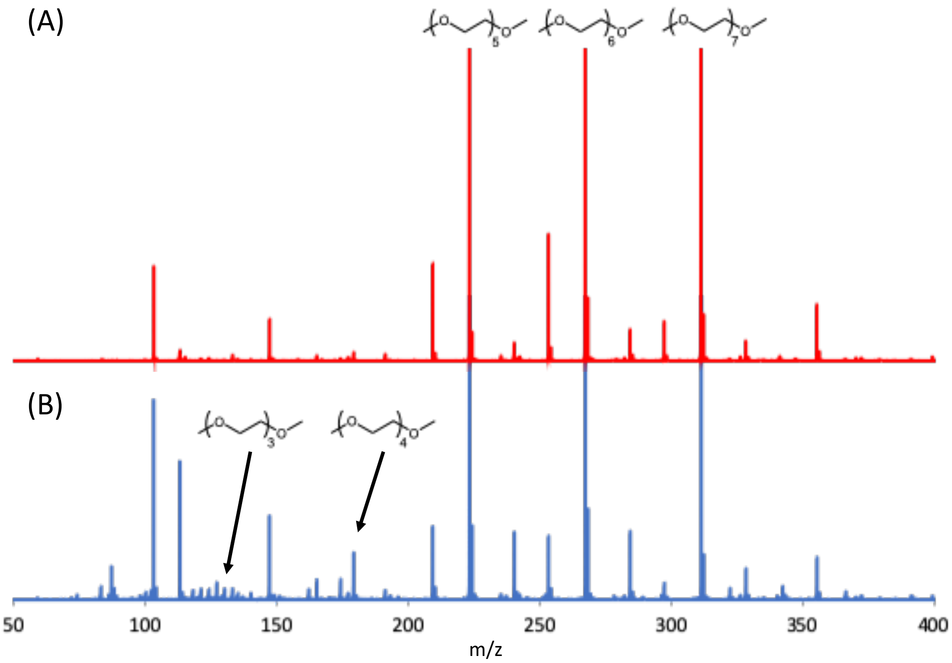

Figure S62. Atmospheric pressure chemical ionization (**APCI**) mass spectrometry of (a) poly(ethylene glycol) dimethyl ether (M_n ~ 250 Da) and (B) the reaction mixture following decarbonylation procedure.

V. Infrared and Resonance Raman Spectra

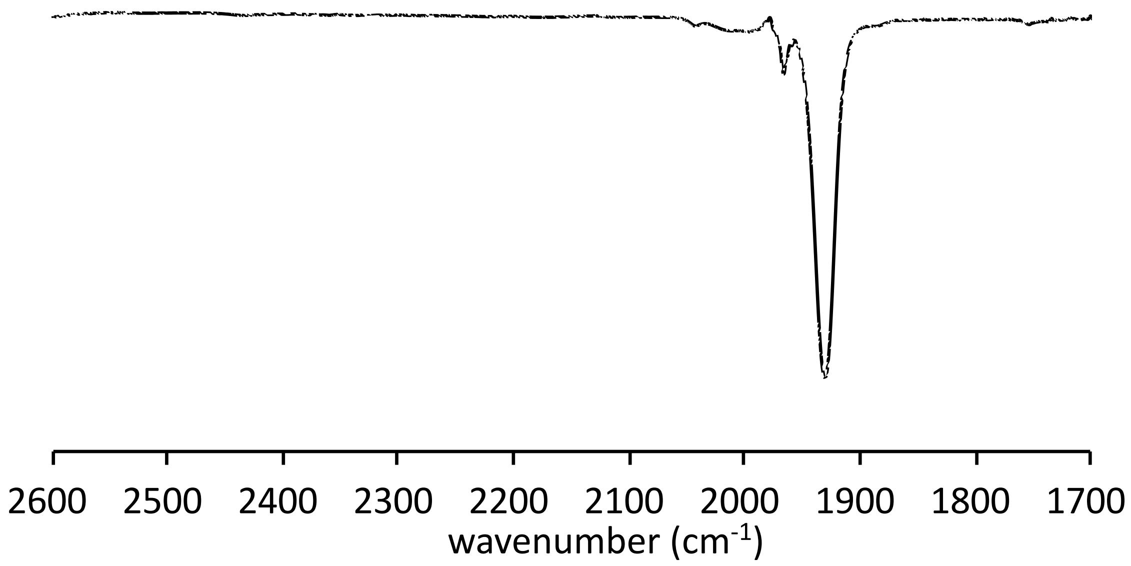

Figure S63. FTIR spectrum of **2**^{18c6} in THF.

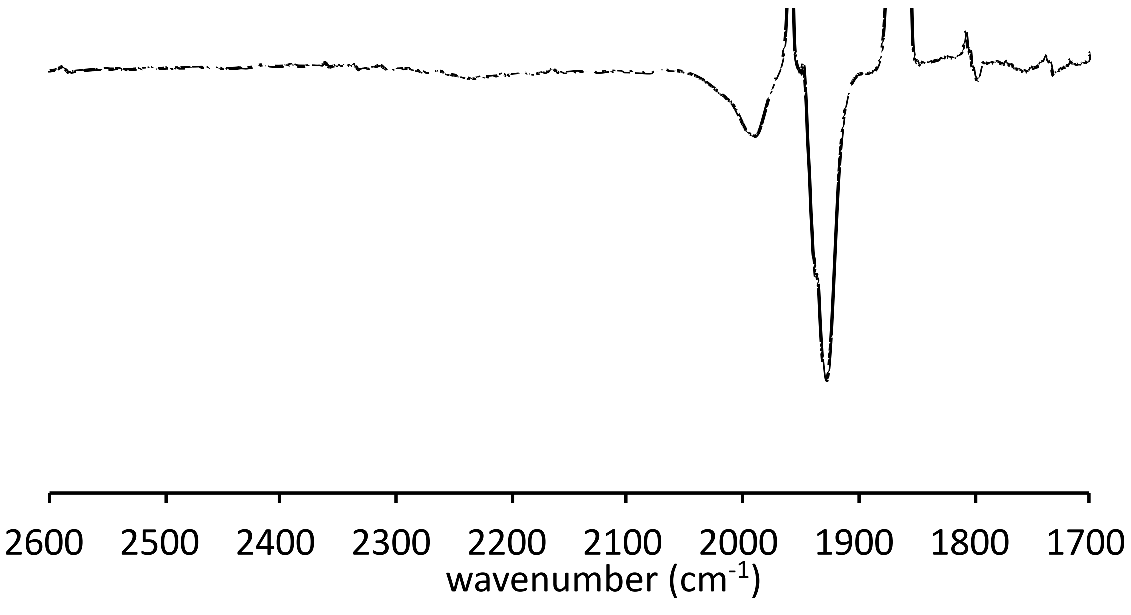

Figure S64. FTIR spectrum of **2**^{15c5} in THF.

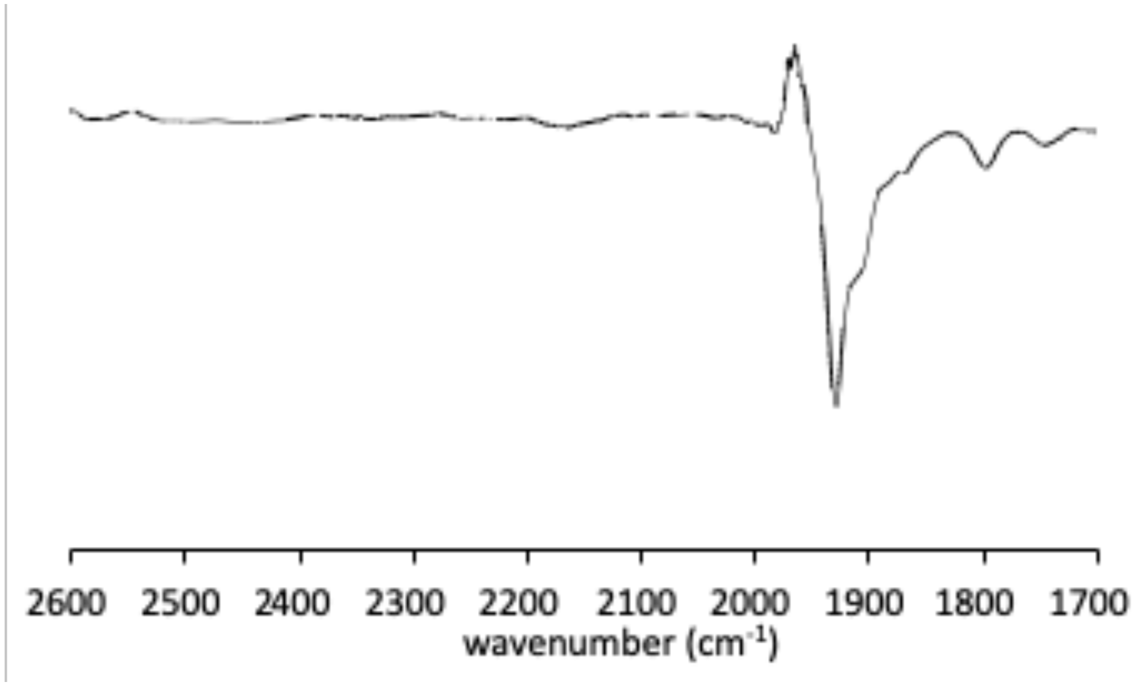

Figure S65. FTIR spectrum of $\mathbf{3}^{\mathbf{BME}}$ and $\mathbf{2}^{\mathbf{BME}}$ in THF.

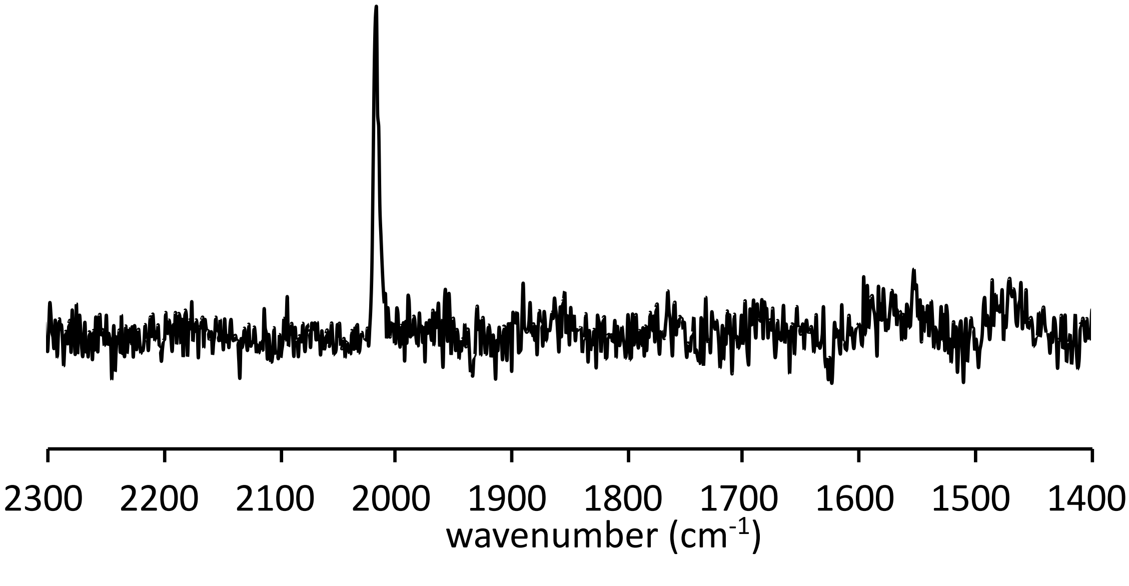

Figure S66. rRaman spectrum of **4** (solid).

VI. Thermodynamics of Ether Decarbonylation

To probe the feasibility of catalysis, the thermodynamic parameters associated with the decarbonylation of diethyl ether and methyl *tert*-butyl ether were assessed. Enthalpy of formation (ΔH^{o}_{f}) and standard entropy (S^{o}) values each species in its standard phase were obtained from the literature cited in the corresponding tables and combined to obtain the desired thermodynamic parameters.

Table S1. Thermodynamics of Et_2O decarbonylation[6]

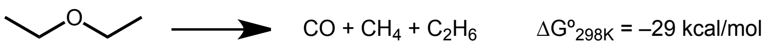

	$Et_2O(l)$	$CO(g)$	$CH_4(g)$	$C_2H_6(g)$
ΔH^{o}_{f} (kJ/mol)	–279.5	–110.5	–74.6	–84
S^{o} (J/mol·K)	172.4	197.7	186.3	229.6

ΔH^{o}_{rxn} = 10.4 kJ/mol

ΔS^{o}_{rxn} = 441.2 J/mol

$\Delta G^{o}_{rxn,298K}$ = –121 kJ/mol = **–28.9 kcal/mol**

Table S2. Thermodynamics of MeO^{t}Bu decarbonylation[6,7]

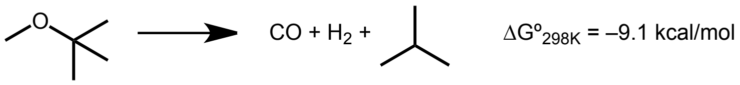

	MeO^{t}Bu(l)	$CO(g)$	$H_2(g)$	isobutane(g)
ΔH^{o}_{f} (kJ/mol)	–313.6	–110.5	0	–134.2
S^{o} (J/mol·K)	265.3	197.7	130.7	295.39

ΔH^{o}_{rxn} = 68.9 kJ/mol

ΔS^{o}_{rxn} = 358.5 J/mol

$\Delta G^{o}_{rxn,298K}$ = –38.0 kJ/mol = **–9.07 kcal/mol**

VII. Crystallographic Details

Data collection. Single-crystal X-ray diffraction data were collected in the University of North Carolina at Chapel Hill Department of Chemistry X-ray Core Laboratory on a Bruker APEX-II CCD diffractometer at 100 ± 2 K or 150 ± 2 K with Cu Kα radiation (λ = 1.54175 Å). Diffraction profiles were integrated using the SAINT software program.[8] Absorption corrections were applied using SADABS.[9]

Structure solution and refinement of $\mathbf{1^{18c6}}$. The structure was solved using direct methods and refined using SHELXTL refinement package[10] via least squares. All non-hydrogen atoms were refined with anisotropic displacement parameters. The hydride was obtained from the difference map and its position was refined. The rest of the hydrogen atoms were placed in ideal positions and refined as riding atoms.

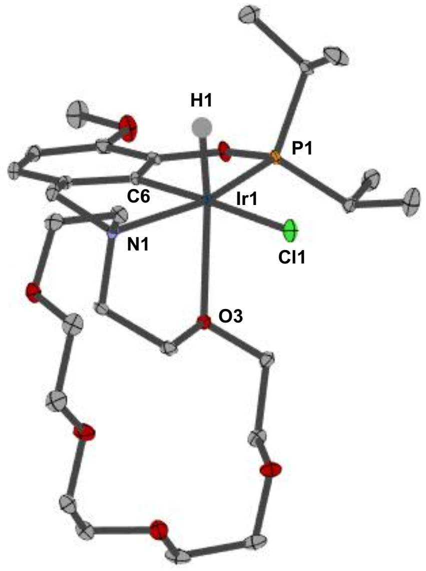

Figure S67. Structural representation of $\mathbf{1^{18c6}}$ with ellipsoids drawn at 50% probability level. The asymmetric unit cell contains two independent molecules of $\mathbf{1^{18c6}}$. One of the molecules is presented. A co-crystallized benzene molecule and hydrogen atoms on the ligand are omitted for clarity.

Table S3. Crystal data and structure refinement for $\mathbf{1^{18c6}}$.

Identification code	x1801001
Empirical formula	$C_{32}H_{52}NO_7PClIr$
Formula weight	821.36
Temperature/K	100(2)

Crystal system	orthorhombic
Space group	Pbca
a/Å	21.5143(8)
b/Å	11.8302(4)
c/Å	26.8208(11)
α/°	90
β/°	90
γ/°	90
Volume/Å^3	6826.4(4)
Z	8
ρ_{calc}g/cm^3	1.598
μ/mm^{-1}	9.110
F(000)	3328.0
Crystal size/mm^3	0.254 × 0.218 × 0.141
Radiation	CuKα (λ = 1.54178)
2Θ range for data collection/°	8.22 to 145.23
Index ranges	-26 ≤ h ≤ 26, -14 ≤ k ≤ 14, -30 ≤ l ≤ 25
Reflections collected	73715
Independent reflections	6616 [R_{int} = 0.0281, R_{sigma} = 0.0125]
Data/restraints/parameters	6616/0/398
Goodness-of-fit on F^2	1.246
Final R indexes [I>=2σ (I)]	R_1 = 0.0241, wR_2 = 0.0542
Final R indexes [all data]	R_1 = 0.0243, wR_2 = 0.0543
Largest diff. peak/hole / e Å^{-3}	0.78/-1.16

Table S4. Bond Lengths for **1^{18c6}**.

**Atom**	**Atom**	**Length/Å**	**Atom**	**Atom**	**Length/Å**
Ir1	C6	1.973(3)	C12	C13	1.535(4)
Ir1	P1	2.1935(6)	C15	C16	1.502(4)
Ir1	N1	2.215(2)	C16	O3	1.450(3)
Ir1	O3	2.3618(18)	O3	C17	1.446(3)
Ir1	Cl1	2.4531(6)	C17	C18	1.516(4)
P1	O1	1.6720(19)	C18	O4	1.428(3)
P1	C12	1.836(3)	O4	C19	1.424(3)
P1	C9	1.839(3)	C19	C20	1.510(4)
N1	C26	1.491(3)	C20	O5	1.423(3)
N1	C15	1.501(3)	O5	C21	1.424(3)
N1	C7	1.509(3)	C21	C22	1.495(4)
C1	C6	1.379(4)	C22	O6	1.417(3)
C1	O1	1.394(3)	O6	C23	1.421(4)
C1	C2	1.401(4)	C23	C24	1.498(4)
C2	O2	1.365(3)	C24	O7	1.424(3)

C2	C3	1.391(4)	O7	C25	1.423(3)
C3	C4	1.402(4)	C25	C26	1.528(4)
C4	C5	1.394(4)	C27	C28	1.369(8)
C5	C6	1.401(4)	C27	C32	1.370(8)
C5	C7	1.511(4)	C28	C29	1.400(7)
O2	C8	1.430(3)	C29	C30	1.376(7)
C9	C10	1.529(4)	C30	C31	1.370(7)
C9	C11	1.538(4)	C31	C32	1.375(7)
C12	C14	1.529(4)			

Table S5. Bond Angles for $\mathbf{1^{18c6}}$.

**Atom**	**Atom**	**Atom**	**Angle/°**	**Atom**	**Atom**	**Atom**	**Angle/°**
C6	Ir1	P1	81.41(8)	C5	C6	Ir1	119.2(2)
C6	Ir1	N1	79.93(10)	N1	C7	C5	110.0(2)
P1	Ir1	N1	161.20(6)	C1	O1	P1	112.95(16)
C6	Ir1	O3	94.52(9)	C2	O2	C8	117.4(2)
P1	Ir1	O3	105.96(5)	C10	C9	C11	110.0(2)
N1	Ir1	O3	77.85(7)	C10	C9	P1	111.20(19)
C6	Ir1	Cl1	176.01(8)	C11	C9	P1	110.88(19)
P1	Ir1	Cl1	102.58(2)	C14	C12	C13	111.3(2)
N1	Ir1	Cl1	96.09(6)	C14	C12	P1	113.86(19)
O3	Ir1	Cl1	84.39(5)	C13	C12	P1	110.0(2)
O1	P1	C12	99.98(11)	N1	C15	C16	112.4(2)
O1	P1	C9	102.85(11)	O3	C16	C15	105.4(2)
C12	P1	C9	104.47(12)	C17	O3	C16	114.01(19)
O1	P1	Ir1	104.81(7)	C17	O3	Ir1	120.11(15)
C12	P1	Ir1	123.28(9)	C16	O3	Ir1	102.86(15)
C9	P1	Ir1	118.04(9)	O3	C17	C18	114.3(2)
C26	N1	C15	112.4(2)	O4	C18	C17	108.3(2)
C26	N1	C7	110.6(2)	C19	O4	C18	113.7(2)
C15	N1	C7	108.1(2)	O4	C19	C20	112.9(2)
C26	N1	Ir1	110.99(15)	O5	C20	C19	108.5(2)
C15	N1	Ir1	107.70(15)	C20	O5	C21	111.9(2)
C7	N1	Ir1	106.81(15)	O5	C21	C22	109.5(2)
C6	C1	O1	118.1(2)	O6	C22	C21	109.3(2)
C6	C1	C2	121.6(2)	C22	O6	C23	111.7(2)
O1	C1	C2	120.4(2)	O6	C23	C24	108.4(2)
O2	C2	C3	125.4(2)	O7	C24	C23	108.6(2)
O2	C2	C1	116.5(2)	C25	O7	C24	112.1(2)
C3	C2	C1	118.1(3)	O7	C25	C26	114.2(2)

C2	C3	C4	121.0(3)	N1	C26	C25	117.5(2)
C5	C4	C3	120.0(2)	C28	C27	C32	121.1(5)
C4	C5	C6	119.2(2)	C27	C28	C29	119.0(5)
C4	C5	C7	126.2(2)	C30	C29	C28	119.8(5)
C6	C5	C7	114.4(2)	C31	C30	C29	119.9(4)
C1	C6	C5	120.0(2)	C30	C31	C32	120.7(5)
C1	C6	Ir1	120.72(19)	C27	C32	C31	119.5(5)

Structure solution and refinement of 1^{BME}. The structure was solved using Superflip[11] and refined (full-matrix-least squares) using the Oxford University Crystals for Windows system.[12] The charge-flipping solution provided most non-hydrogen atoms from the E-map. Full-matrix least squares / difference Fourier cycles were performed, which located the remaining non-hydrogen atoms. All non-hydrogen atoms were refined with anisotropic displacement parameters. The hydride was obtained from the difference map and its position was refined. The rest of the hydrogen atoms were placed in ideal positions and refined as riding atoms.

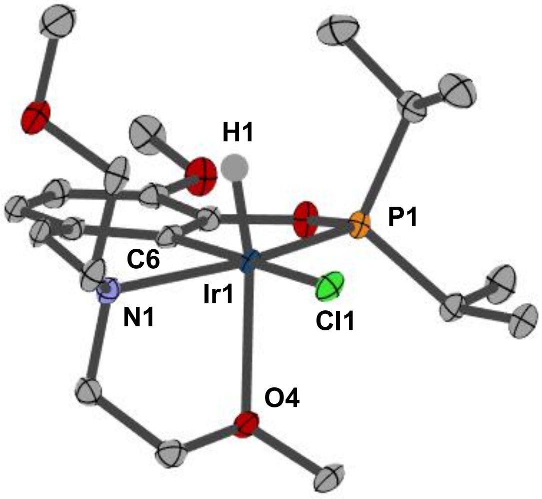

Figure S68. Structural representation of 1^{BME} with ellipsoids drawn at 50% probability level. Hydrogen atoms on the ligand are omitted for clarity.

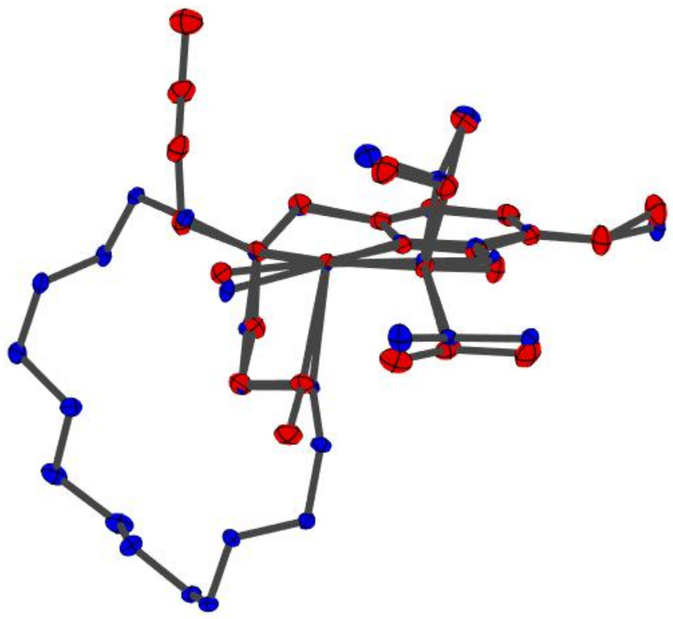

Figure S69. Overlay of structural representations of $\mathbf{1}^{\mathbf{18c6}}$ (1, blue) and $\mathbf{1}^{\mathbf{BME}}$ (red) derived from X- ray diffraction analysis. Hydrogen atoms and counterions omitted for clarity.

Table S6. Crystal data and structure refinement for $\mathbf{1}^{\mathbf{BME}}$.

Identification code	19078_0ma
Empirical formula	$C_{37}H_{66}N_2O_4P_2Cl_4Ir_2$
Formula weight	1191.05
Temperature/K	150(2)
Crystal system	triclinic
Space group	P-1
a/Å	11.7045(5)
b/Å	14.2405(6)
c/Å	14.5938(6)
α/°	72.905(2)
β/°	75.656(2)
γ/°	88.106(2)
Volume/Å^3	2250.44(17)
Z	2
ρ_{calc}g/cm^3	1.758
μ/mm^{-1}	14.437
F(000)	1172.0
Crystal size/mm^3	? × ? × ?
Radiation	CuKα (λ = 1.54178)
2Θ range for data collection/°	6.5 to 133.518
Index ranges	$-13 \le h \le 13$, $-16 \le k \le 14$, $-17 \le l \le 17$

Reflections collected	43101
Independent reflections	7788 [R_{int} = 0.0644, R_{sigma} = 0.0519]
Data/restraints/parameters	7788/0/482
Goodness-of-fit on F^2	1.110
Final R indexes [I>=2σ (I)]	R_1 = 0.0451, wR_2 = 0.1245
Final R indexes [all data]	R_1 = 0.0500, wR_2 = 0.1282
Largest diff. peak/hole / e Å^{-3}	2.92/-1.26

Table S7. Bond Lengths for $\mathbf{1^{BME}}$.

Atom	Atom	Length/Å	Atom	Atom	Length/Å
Ir1	C6	1.988(7)	Ir2	P2	2.1982(19)
Ir1	P1	2.2008(17)	Ir2	N2	2.279(6)
Ir1	N1	2.289(6)	Ir2	Cl2	2.5574(16)
Ir1	Cl1	2.5519(15)	Ir2	Cl2^{2}	2.6091(17)
Ir1	Cl1^{1}	2.6017(16)	Cl2	Ir2^{2}	2.6090(17)
Cl1	Ir1^{1}	2.6017(16)	N2	C33	1.493(9)
N1	C17	1.496(8)	N2	C25	1.492(10)
N1	C7	1.497(9)	N2	C35	1.508(9)
N1	C15	1.511(10)	P2	O3	1.676(5)
P1	O1	1.650(5)	P2	C27	1.826(9)
P1	C12	1.825(8)	P2	C30	1.844(9)
P1	C9	1.827(9)	C19	C24	1.380(11)
C1	C6	1.384(10)	C19	O3	1.382(10)
C1	O1	1.390(10)	C19	C20	1.401(11)
C1	C2	1.397(10)	C20	C21	1.363(13)
C2	O2	1.378(10)	C20	O4	1.378(10)
C2	C3	1.381(13)	C21	C22	1.409(12)
C3	C4	1.378(12)	C22	C23	1.390(10)
C4	C5	1.394(10)	C23	C24	1.399(10)
C5	C6	1.394(10)	C23	C25	1.499(11)
C5	C7	1.496(10)	O4	C26	1.444(11)
O2	C8	1.436(11)	C27	C29	1.533(13)
C9	C11	1.538(17)	C27	C28	1.535(14)
C9	C10	1.541(16)	C30	C32	1.509(14)
C12	C13	1.530(13)	C30	C31	1.546(14)
C12	C14	1.536(11)	C33	C34	1.540(10)
C15	C16	1.495(12)	C35	C36	1.500(13)
C17	C18	1.531(10)	C37	Cl3	1.69(3)
Ir2	C24	1.984(7)	C37	Cl4	1.73(3)

^{1}1-X,1-Y,-Z; ^{2}-X,2-Y,-Z

Table S8. Bond Angles for **1**$^{\mathbf{BME}}$.

Atom	Atom	Atom	Angle/°	Atom	Atom	Atom	Angle/°
C6	Ir1	P1	80.3(2)	C24	Ir2	N2	76.8(3)
C6	Ir1	N1	76.9(2)	P2	Ir2	N2	153.50(16)
P1	Ir1	N1	153.65(15)	C24	Ir2	Cl2	172.5(2)
C6	Ir1	Cl1	172.6(2)	P2	Ir2	Cl2	107.19(6)
P1	Ir1	Cl1	106.95(6)	N2	Ir2	Cl2	95.80(16)
N1	Ir1	Cl1	95.75(14)	C24	Ir2	$Cl2^{2}$	103.8(2)
C6	Ir1	$Cl1^{1}$	102.7(2)	P2	Ir2	$Cl2^{2}$	108.21(6)
P1	Ir1	$Cl1^{1}$	108.00(6)	N2	Ir2	$Cl2^{2}$	89.56(15)
N1	Ir1	$Cl1^{1}$	89.85(16)	Cl2	Ir2	$Cl2^{2}$	76.51(6)
Cl1	Ir1	$Cl1^{1}$	76.70(6)	Ir2	Cl2	$Ir2^{2}$	103.49(6)
Ir1	Cl1	$Ir1^{1}$	103.31(6)	C33	N2	C25	109.5(6)
C17	N1	C7	109.0(6)	C33	N2	C35	110.9(6)
C17	N1	C15	111.5(6)	C25	N2	C35	108.0(6)
C7	N1	C15	108.0(6)	C33	N2	Ir2	114.5(4)
C17	N1	Ir1	115.7(4)	C25	N2	Ir2	103.4(4)
C7	N1	Ir1	103.4(4)	C35	N2	Ir2	110.1(5)
C15	N1	Ir1	108.7(5)	O3	P2	C27	97.8(4)
O1	P1	C12	97.3(3)	O3	P2	C30	100.1(4)
O1	P1	C9	101.3(4)	C27	P2	C30	105.2(4)
C12	P1	C9	105.2(4)	O3	P2	Ir2	104.8(2)
O1	P1	Ir1	105.2(2)	C27	P2	Ir2	124.6(3)
C12	P1	Ir1	125.0(3)	C30	P2	Ir2	119.2(3)
C9	P1	Ir1	118.2(3)	C24	C19	O3	117.0(7)
C6	C1	O1	116.7(6)	C24	C19	C20	121.7(8)
C6	C1	C2	122.1(8)	O3	C19	C20	121.3(7)
O1	C1	C2	121.1(7)	C21	C20	O4	126.5(7)
O2	C2	C3	126.9(7)	C21	C20	C19	118.9(8)
O2	C2	C1	115.3(8)	O4	C20	C19	114.6(8)
C3	C2	C1	117.8(7)	C20	C21	C22	120.7(7)
C4	C3	C2	121.0(7)	C23	C22	C21	119.7(8)
C3	C4	C5	120.7(8)	C22	C23	C24	119.8(7)
C6	C5	C4	119.2(7)	C22	C23	C25	125.8(7)
C6	C5	C7	114.3(6)	C24	C23	C25	114.3(6)
C4	C5	C7	126.4(7)	C19	C24	C23	119.0(7)
C1	C6	C5	118.9(6)	C19	C24	Ir2	121.8(6)
C1	C6	Ir1	121.3(6)	C23	C24	Ir2	118.9(6)
C5	C6	Ir1	119.4(5)	C19	O3	P2	112.8(5)
C5	C7	N1	108.1(6)	C20	O4	C26	116.6(9)
C1	O1	P1	113.8(4)	N2	C25	C23	107.8(6)
C2	O2	C8	115.9(8)	C29	C27	C28	109.4(8)

C11	C9	C10	114.7(11)	C29	C27	P2	115.7(7)
C11	C9	P1	110.2(7)	C28	C27	P2	109.3(6)
C10	C9	P1	111.7(8)	C32	C30	C31	111.0(9)
C13	C12	C14	109.3(8)	C32	C30	P2	112.7(7)
C13	C12	P1	110.1(6)	C31	C30	P2	110.4(6)
C14	C12	P1	114.5(6)	N2	C33	C34	114.7(6)
C16	C15	N1	115.4(7)	C36	C35	N2	114.4(7)
N1	C17	C18	114.6(6)	Cl3	C37	Cl4	113.5(18)
C24	Ir2	P2	79.9(2)				

[1]1-X,1-Y,-Z; [2]-X,2-Y,-Z

Structure solution and refinement of 1^{Et}. The structure was solved using direct methods and refined using SHELXTL refinement package[10] via least squares. All non-hydrogen atoms were refined with anisotropic displacement parameters. The hydride was obtained from the difference map and its position was refined. The rest of the hydrogen atoms were placed in ideal positions and refined as riding atoms.

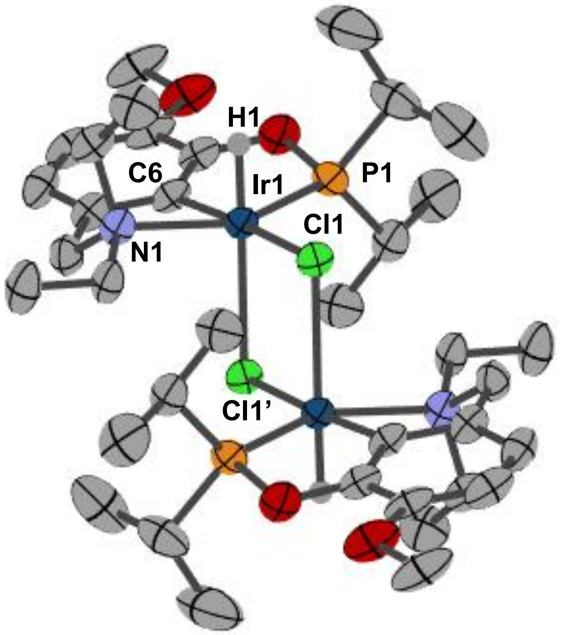

Figure S70. Structural representation of $\mathbf{1^{Et}}$ with ellipsoids drawn at 50% probability level. The asymmetric unit cell contains two independent molecules of 1^{Et}. One of the molecules is presented. A co-crystallized dichloromethane molecule and hydrogen atoms on the ligand are omitted for clarity.

Table S9. Crystal data and structure refinement for **1^{Et}**.

Identification code	19078_0ma
Empirical formula	$C_{37}H_{66}N_2O_4P_2Cl_4Ir_2$
Formula weight	1191.05
Temperature/K	150(2)
Crystal system	triclinic
Space group	P-1
a/Å	11.7045(5)
b/Å	14.2405(6)
c/Å	14.5938(6)
α/°	72.905(2)
β/°	75.656(2)
γ/°	88.106(2)
Volume/Å^3	2250.44(17)
Z	2
ρ_{calc}g/cm^3	1.758
μ/mm^{-1}	14.437
F(000)	1172.0
Crystal size/mm^3	? × ? × ?
Radiation	CuKα (λ = 1.54178)
2Θ range for data collection/°	6.5 to 133.518
Index ranges	-13 ≤ h ≤ 13, -16 ≤ k ≤ 14, -17 ≤ l ≤ 17
Reflections collected	43101
Independent reflections	7788 [R_{int} = 0.0644, R_{sigma} = 0.0519]
Data/restraints/parameters	7788/0/482
Goodness-of-fit on F^2	1.110
Final R indexes [I>=2σ (I)]	R_1 = 0.0451, wR_2 = 0.1245
Final R indexes [all data]	R_1 = 0.0500, wR_2 = 0.1282
Largest diff. peak/hole / e Å^{-3}	2.92/-1.26

Table S10. Bond Lengths for **1^{Et}**.

**Atom**	**Atom**	**Length/Å**	**Atom**	**Atom**	**Length/Å**
Ir1	C6	1.988(7)	Ir2	P2	2.1982(19)
Ir1	P1	2.2008(17)	Ir2	N2	2.279(6)
Ir1	N1	2.289(6)	Ir2	Cl2	2.5574(16)
Ir1	Cl1	2.5519(15)	Ir2	Cl2^2	2.6091(17)
Ir1	Cl1^1	2.6017(16)	Cl2	Ir2^2	2.6090(17)
Cl1	Ir1^1	2.6017(16)	N2	C33	1.493(9)
N1	C17	1.496(8)	N2	C25	1.492(10)
N1	C7	1.497(9)	N2	C35	1.508(9)
N1	C15	1.511(10)	P2	O3	1.676(5)

P1	O1	1.650(5)	P2	C27	1.826(9)
P1	C12	1.825(8)	P2	C30	1.844(9)
P1	C9	1.827(9)	C19	C24	1.380(11)
C1	C6	1.384(10)	C19	O3	1.382(10)
C1	O1	1.390(10)	C19	C20	1.401(11)
C1	C2	1.397(10)	C20	C21	1.363(13)
C2	O2	1.378(10)	C20	O4	1.378(10)
C2	C3	1.381(13)	C21	C22	1.409(12)
C3	C4	1.378(12)	C22	C23	1.390(10)
C4	C5	1.394(10)	C23	C24	1.399(10)
C5	C6	1.394(10)	C23	C25	1.499(11)
C5	C7	1.496(10)	O4	C26	1.444(11)
O2	C8	1.436(11)	C27	C29	1.533(13)
C9	C11	1.538(17)	C27	C28	1.535(14)
C9	C10	1.541(16)	C30	C32	1.509(14)
C12	C13	1.530(13)	C30	C31	1.546(14)
C12	C14	1.536(11)	C33	C34	1.540(10)
C15	C16	1.495(12)	C35	C36	1.500(13)
C17	C18	1.531(10)	C37	Cl3	1.69(3)
Ir2	C24	1.984(7)	C37	Cl4	1.73(3)

[1]1-X,1-Y,-Z; [2]-X,2-Y,-Z

Table S11. Bond Angles for $\mathbf{1^{Et}}$.

Atom	Atom	Atom	Angle/°	Atom	Atom	Atom	Angle/°
C6	Ir1	P1	80.3(2)	C24	Ir2	N2	76.8(3)
C6	Ir1	N1	76.9(2)	P2	Ir2	N2	153.50(16)
P1	Ir1	N1	153.65(15)	C24	Ir2	Cl2	172.5(2)
C6	Ir1	Cl1	172.6(2)	P2	Ir2	Cl2	107.19(6)
P1	Ir1	Cl1	106.95(6)	N2	Ir2	Cl2	95.80(16)
N1	Ir1	Cl1	95.75(14)	C24	Ir2	$Cl2^2$	103.8(2)
C6	Ir1	$Cl1^1$	102.7(2)	P2	Ir2	$Cl2^2$	108.21(6)
P1	Ir1	$Cl1^1$	108.00(6)	N2	Ir2	$Cl2^2$	89.56(15)
N1	Ir1	$Cl1^1$	89.85(16)	Cl2	Ir2	$Cl2^2$	76.51(6)
Cl1	Ir1	$Cl1^1$	76.70(6)	Ir2	Cl2	$Ir2^2$	103.49(6)
Ir1	Cl1	$Ir1^1$	103.31(6)	C33	N2	C25	109.5(6)
C17	N1	C7	109.0(6)	C33	N2	C35	110.9(6)
C17	N1	C15	111.5(6)	C25	N2	C35	108.0(6)
C7	N1	C15	108.0(6)	C33	N2	Ir2	114.5(4)
C17	N1	Ir1	115.7(4)	C25	N2	Ir2	103.4(4)
C7	N1	Ir1	103.4(4)	C35	N2	Ir2	110.1(5)
C15	N1	Ir1	108.7(5)	O3	P2	C27	97.8(4)

O1	P1	C12	97.3(3)	O3	P2	C30	100.1(4)
O1	P1	C9	101.3(4)	C27	P2	C30	105.2(4)
C12	P1	C9	105.2(4)	O3	P2	Ir2	104.8(2)
O1	P1	Ir1	105.2(2)	C27	P2	Ir2	124.6(3)
C12	P1	Ir1	125.0(3)	C30	P2	Ir2	119.2(3)
C9	P1	Ir1	118.2(3)	C24	C19	O3	117.0(7)
C6	C1	O1	116.7(6)	C24	C19	C20	121.7(8)
C6	C1	C2	122.1(8)	O3	C19	C20	121.3(7)
O1	C1	C2	121.1(7)	C21	C20	O4	126.5(7)
O2	C2	C3	126.9(7)	C21	C20	C19	118.9(8)
O2	C2	C1	115.3(8)	O4	C20	C19	114.6(8)
C3	C2	C1	117.8(7)	C20	C21	C22	120.7(7)
C4	C3	C2	121.0(7)	C23	C22	C21	119.7(8)
C3	C4	C5	120.7(8)	C22	C23	C24	119.8(7)
C6	C5	C4	119.2(7)	C22	C23	C25	125.8(7)
C6	C5	C7	114.3(6)	C24	C23	C25	114.3(6)
C4	C5	C7	126.4(7)	C19	C24	C23	119.0(7)
C1	C6	C5	118.9(6)	C19	C24	Ir2	121.8(6)
C1	C6	Ir1	121.3(6)	C23	C24	Ir2	118.9(6)
C5	C6	Ir1	119.4(5)	C19	O3	P2	112.8(5)
C5	C7	N1	108.1(6)	C20	O4	C26	116.6(9)
C1	O1	P1	113.8(4)	N2	C25	C23	107.8(6)
C2	O2	C8	115.9(8)	C29	C27	C28	109.4(8)
C11	C9	C10	114.7(11)	C29	C27	P2	115.7(7)
C11	C9	P1	110.2(7)	C28	C27	P2	109.3(6)
C10	C9	P1	111.7(8)	C32	C30	C31	111.0(9)
C13	C12	C14	109.3(8)	C32	C30	P2	112.7(7)
C13	C12	P1	110.1(6)	C31	C30	P2	110.4(6)
C14	C12	P1	114.5(6)	N2	C33	C34	114.7(6)
C16	C15	N1	115.4(7)	C36	C35	N2	114.4(7)
N1	C17	C18	114.6(6)	Cl3	C37	Cl4	113.5(18)
C24	Ir2	P2	79.9(2)				

[1]1-X,1-Y,-Z; [2]-X,2-Y,-Z

Structure solution and refinement of 5. The structure was solved using direct methods and refined using SHELXTL refinement package[10] via least squares. All non-hydrogen atoms were refined with anisotropic displacement parameters. The hydride was obtained from the difference map and its position was refined. The rest of the hydrogen atoms were placed in ideal positions and refined as riding atoms.

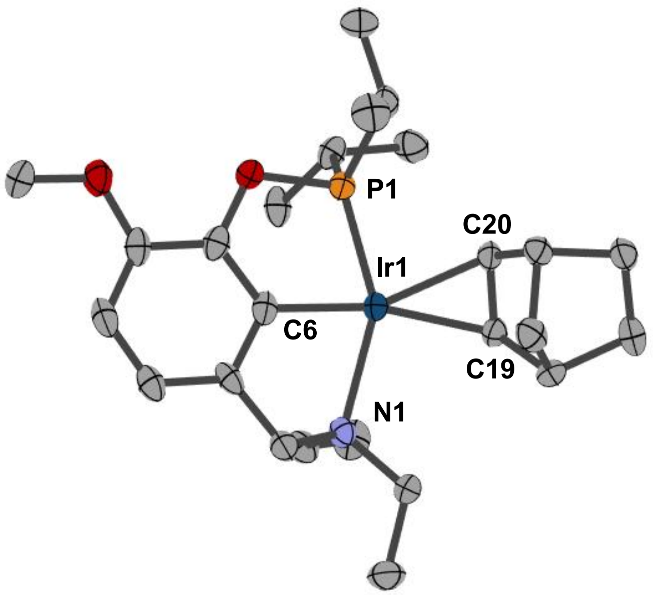

Figure S71. Structural representation of **5** with ellipsoids drawn at 50% probability level. Hydrogen atoms are omitted for clarity.

Table S12. Crystal data and structure refinement for 5.

Identification code	20032_0m
Empirical formula	$C_{25}H_{41}IrNO_2P$
Formula weight	610.76
Temperature/K	150(2)
Crystal system	monoclinic
Space group	$P2_1/n$
a/Å	9.6922(9)
b/Å	38.687(4)
c/Å	13.5664(17)
α/°	90
β/°	104.749(9)
γ/°	90
Volume/Å^3	4919.3(9)
Z	8
ρ_{calc}g/cm^3	1.649

μ/mm^{-1}	11.276
F(000)	2448.0
Crystal size/mm^3	0.15 × 0.05 × 0.05
Radiation	CuKα (λ = 1.54178)
2Θ range for data collection/°	4.568 to 140.302
Index ranges	-11 ≤ h ≤ 11, -44 ≤ k ≤ 47, -16 ≤ l ≤ 11
Reflections collected	27058
Independent reflections	8891 [R_{int} = 0.0886, R_{sigma} = 0.1177]
Data/restraints/parameters	8891/0/555
Goodness-of-fit on F^2	1.024
Final R indexes [I>=2σ (I)]	R_1 = 0.0511, wR_2 = 0.1242
Final R indexes [all data]	R_1 = 0.0662, wR_2 = 0.1318
Largest diff. peak/hole / e Å^{-3}	1.20/-1.14

Table S13. Bond Lengths for 5.

Atom	Atom	Length/Å	Atom	Atom	Length/Å
C1	O1	1.381(11)	C27	C28	1.384(12)
C1	C2	1.397(12)	C27	O4	1.392(9)
C1	C6	1.402(11)	C28	C29	1.393(13)
C2	O2	1.380(11)	C29	C30	1.394(12)
C2	C3	1.397(13)	C30	C31	1.397(11)
C3	C4	1.388(13)	C30	C32	1.502(12)
C4	C5	1.403(11)	C31	Ir2	1.999(8)
C5	C6	1.407(12)	C32	N2	1.505(11)
C5	C7	1.492(11)	C33	O4	1.420(11)
C6	Ir1	2.006(8)	C34	C36	1.527(11)
C7	N1	1.494(11)	C34	C35	1.527(13)
C8	O2	1.405(11)	C34	P2	1.847(9)
C9	C10	1.507(13)	C37	C39	1.525(9)
C9	C11	1.543(14)	C37	C38	1.530(12)
C9	P1	1.863(9)	C37	P2	1.826(8)
C12	C14	1.508(11)	C40	C41	1.516(14)
C12	C13	1.535(12)	C40	N2	1.517(12)
C12	P1	1.850(9)	C42	C43	1.509(14)
C15	N1	1.499(10)	C42	N2	1.519(12)
C15	C16	1.539(12)	C44	C45	1.419(12)
C17	N1	1.494(10)	C44	C49	1.531(12)
C17	C18	1.501(14)	C44	Ir2	2.194(8)
C19	C20	1.406(11)	C45	C46	1.522(13)
C19	C24	1.545(11)	C45	Ir2	2.178(8)
C19	Ir1	2.175(7)	C46	C50	1.546(13)

C20	C21	1.516(11)	C46	C47	1.568(13)
C20	Ir1	2.155(8)	C47	C48	1.508(14)
C21	C22	1.558(11)	C48	C49	1.564(11)
C21	C25	1.559(12)	C49	C50	1.553(11)
C22	C23	1.528(13)	Ir1	P1	2.184(2)
C23	C24	1.567(12)	Ir1	N1	2.252(7)
C24	C25	1.531(12)	Ir2	P2	2.1814(18)
C26	O3	1.393(9)	Ir2	N2	2.248(7)
C26	C27	1.395(11)	O1	P1	1.672(6)
C26	C31	1.417(10)	O3	P2	1.662(6)

Table S14. Bond Angles for 5.

Atom	Atom	Atom	Angle/°	Atom	Atom	Atom	Angle/°
O1	C1	C2	120.6(7)	C45	C44	Ir2	70.4(5)
O1	C1	C6	116.6(8)	C49	C44	Ir2	120.3(6)
C2	C1	C6	122.8(9)	C44	C45	C46	105.3(8)
O2	C2	C1	115.0(8)	C44	C45	Ir2	71.7(5)
O2	C2	C3	125.5(8)	C46	C45	Ir2	124.0(6)
C1	C2	C3	119.5(8)	C45	C46	C50	101.2(6)
C4	C3	C2	119.6(8)	C45	C46	C47	105.1(7)
C3	C4	C5	120.1(8)	C50	C46	C47	100.9(7)
C4	C5	C6	122.0(8)	C48	C47	C46	103.5(7)
C4	C5	C7	124.3(8)	C47	C48	C49	104.7(7)
C6	C5	C7	113.8(7)	C44	C49	C50	101.2(7)
C1	C6	C5	116.1(8)	C44	C49	C48	103.0(7)
C1	C6	Ir1	123.6(7)	C50	C49	C48	99.5(6)
C5	C6	Ir1	120.1(6)	C46	C50	C49	94.3(7)
C5	C7	N1	108.0(7)	C6	Ir1	C20	152.8(3)
C10	C9	C11	111.8(8)	C6	Ir1	C19	167.3(3)
C10	C9	P1	108.5(7)	C20	Ir1	C19	37.9(3)
C11	C9	P1	114.4(6)	C6	Ir1	P1	78.3(3)
C14	C12	C13	112.6(7)	C20	Ir1	P1	81.3(2)
C14	C12	P1	109.0(6)	C19	Ir1	P1	114.4(2)
C13	C12	P1	111.4(6)	C6	Ir1	N1	76.1(3)
N1	C15	C16	117.0(7)	C20	Ir1	N1	128.5(3)
N1	C17	C18	116.1(8)	C19	Ir1	N1	91.7(3)
C20	C19	C24	104.6(7)	P1	Ir1	N1	148.25(18)
C20	C19	Ir1	70.3(4)	C31	Ir2	C45	166.1(3)
C24	C19	Ir1	121.7(6)	C31	Ir2	P2	79.1(2)
C19	C20	C21	107.9(7)	C45	Ir2	P2	113.1(2)

C19	C20	Ir1	71.8(5)	C31	Ir2	C44	156.0(3)
C21	C20	Ir1	119.3(6)	C45	Ir2	C44	37.9(3)
C20	C21	C22	104.8(7)	P2	Ir2	C44	79.0(2)
C20	C21	C25	101.7(6)	C31	Ir2	N2	77.2(3)
C22	C21	C25	98.7(7)	C45	Ir2	N2	89.9(3)
C23	C22	C21	104.1(7)	P2	Ir2	N2	155.66(19)
C22	C23	C24	103.1(7)	C44	Ir2	N2	125.3(3)
C25	C24	C19	101.8(6)	C17	N1	C7	106.8(6)
C25	C24	C23	100.3(7)	C17	N1	C15	109.7(6)
C19	C24	C23	105.5(7)	C7	N1	C15	108.8(6)
C24	C25	C21	94.2(7)	C17	N1	Ir1	108.8(5)
O3	C26	C27	119.5(7)	C7	N1	Ir1	106.7(5)
O3	C26	C31	117.5(7)	C15	N1	Ir1	115.7(5)
C27	C26	C31	122.9(7)	C32	N2	C40	108.1(7)
C28	C27	O4	125.3(8)	C32	N2	C42	109.3(7)
C28	C27	C26	119.4(7)	C40	N2	C42	105.2(6)
O4	C27	C26	115.3(7)	C32	N2	Ir2	110.9(5)
C27	C28	C29	119.2(7)	C40	N2	Ir2	112.8(5)
C28	C29	C30	120.8(8)	C42	N2	Ir2	110.4(5)
C29	C30	C31	121.9(8)	C1	O1	P1	111.9(5)
C29	C30	C32	123.5(8)	C2	O2	C8	115.8(7)
C31	C30	C32	114.3(7)	C26	O3	P2	111.2(5)
C30	C31	C26	115.7(7)	C27	O4	C33	116.1(7)
C30	C31	Ir2	122.5(6)	O1	P1	C12	98.2(3)
C26	C31	Ir2	121.8(6)	O1	P1	C9	98.9(3)
C30	C32	N2	110.5(7)	C12	P1	C9	105.1(4)
C36	C34	C35	111.7(8)	O1	P1	Ir1	109.5(2)
C36	C34	P2	111.6(6)	C12	P1	Ir1	115.8(3)
C35	C34	P2	109.3(6)	C9	P1	Ir1	124.8(3)
C39	C37	C38	108.9(7)	O3	P2	C37	98.0(3)
C39	C37	P2	114.5(6)	O3	P2	C34	99.7(4)
C38	C37	P2	108.3(6)	C37	P2	C34	106.3(4)
C41	C40	N2	112.7(7)	O3	P2	Ir2	109.4(2)
C43	C42	N2	115.0(7)	C37	P2	Ir2	122.8(3)
C45	C44	C49	107.0(7)	C34	P2	Ir2	116.6(3)

VIII. References

1 R. K. Harris, E. D. Becker, S. M. Cabral De Menezes, R. Goodfellow and P. Granger, *Pure Appl. Chem.*, 2001, **73**, 1795–1818.

2 L. C. Gregor, J. Grajeda, P. S. White, A. J. Vetter and A. J. M. M. Miller, *Catal. Sci. Technol.*, 2018, **8**, 3133–3143.

3 L. C. Gregor, J. Grajeda, M. R. Kita, P. S. White, A. J. Vetter, A. J. M. Miller, *Organometallics*, 2016, **35**, 3074–3086.

4 J. L. Herde, J. C. Lambert, C. V. Senoff and M. A. Cushing, in *Inorganic Syntheses*, 2007, vol. 15, pp. 18–20.

5 H. M. Dodge, M. R. Kita and A. J. M. Miller, *Identification and Strategic Evasion of an Off-Cycle Iridium Species in Allylbenzene Isomerization*, .

6 David R. Lide, Ed., *CRC handbook of chemistry and physics*, CRC Press, Florida, 84th Ed., 2003.

7 B. A. Younglove and J. F. Ely, *J. Phys. Chem. Ref. Data*, 1987, **16**, 577–798.

8 SAINT, Bruker Analytical X-Ray Systems, Madison, WI, current version.

9 R. H. Blessing, *Acta Crystallogr. Sect. A Found. Crystallogr.*, 1995, **51**, 33–38.

10 G. M. Sheldrick, SHELXL-2013, Program for the Solution of Crystal Structures, University of Göttingen, Germany, 2013.

11 L. Palatinus and G. Chapuis, *J. Appl. Crystallogr.*, 2007, **40**, 786–790.

12 P. W. Betteridge, J. R. Carruthers, R. I. Cooper, K. Prout and D. J. Watkin, *J. Appl. Crystallogr.*, 2003, **36**, 1487–1487.
